# Targeting PRKCN, an Essential Driver Orchestrating mTOR‐IRF4 Axis Independently of Kinase Activity, in Multiple Myeloma

**DOI:** 10.1002/advs.202518975

**Published:** 2026-02-08

**Authors:** Koukou Tang, Dongpeng Jiang, Peng Ke, Xiaosen Bian, Lili Zhang, Ying Zhang, Tingting Zhu, Weiwei Qu, Wenhui Qi, Yang Xu, Chengcheng Fu, Depei Wu, Jianhong Chu

**Affiliations:** ^1^ Institute of Blood and Marrow Transplantation National Clinical Research Center for Hematologic Diseases Jiangsu Institute of Hematology Collaborative Innovation Center of Hematology The First Affiliated Hospital of Soochow University Soochow University Suzhou Jiangsu China; ^2^ Second Clinical Medical College of Jinan University First Affiliated Hospital of Southern University of Science and Technology Shenzhen People's Hospital Shenzhen China

**Keywords:** IRF4, mTOR signaling, multiple myeloma, NF‐κB signaling, protein kinase

## Abstract

Multiple myeloma (MM) remains incurable, necessitating development of novel therapeutic targets. Deregulated PRKCN is implicated in solid tumors, while its role in MM remains elusive. Here, PRKCN is identified as a super‐enhancer‐driven gene associated with adverse prognosis in MM. PRKCN is transactivated by NF‐κB signaling intrinsically existing or exogenously provoked. Constitutive or inducible knockdown of PRKCN significantly impairs cell growth and tumorigenicity, while overcoming drug resistance. PRKCN harnesses IRF4 to exert its effect, and in turn, IRF4 directly induces PRKCN transcription, establishing a feed‐forward IRF4‐PRKCN circuit. Furthermore, PRKCN fosters IRF4 expression by activating mTORC1/C2 signaling pathways via physical interaction with mTOR. Surprisingly, PRKCN modulates mTOR‐IRF4 axis and cell growth independently of its acknowledged kinase activity yet requiring activation loop phosphorylation. Intriguingly, PRKCN silencing evokes interferon signaling and confers increased sensitivity to interferon. Finally, targeting PRKCN with an orally bioavailable inhibitor suppresses MM cell growth and overcomes drug resistance in vitro, and elicits robust efficacy in cell line‐derived xenografts and a patient‐derived xenograft, which is connected with the mitigated PRKCN expression and activation loop phosphorylation as well as blunted mTOR‐IRF4 axis. Collectively, our study delineates PRKCN function that links aberrant NF‐κB signaling and mTOR‐IRF4 axis, supporting clinically targeting PRKCN in MM.

## Graphical abstract

Constitutive PRKCN expression is driven by super‐enhancers and modulated by NF‐κB signaling in multiple myeloma (MM). PRKCN activates mTORC1/2‐IRF4 signaling axis and favors tumor cell growth independently of its kinase activity. IRF4 reciprocally promotes PRKCN transcription, creating a feed‐forward loop. CRT0066101 diminishes PRKCN expression and activation loop phosphorylation, and disturbs the mTORC1/C2‐IRF4 axis, thereby exhibiting robust anti‐MM efficacy.

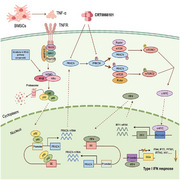

## 1 Introduction

Multiple myeloma (MM) is a common hematological malignancy featured by clonal expansion of malignant plasma cells in bone marrow. Although the overall patient survival has remarkably improved due to the application of highly active agents such as lenalidomide and bortezomib, MM remains largely incurable with acquired drug resistance [1, 2, 3]. Therefore, there is an unmet need to decipher the pathogenesis to identify novel and therapeutic targets for resolving this dilemma.

PRKCN belongs to the multigene protein kinase D family of serine/threonine kinases. It is involved in various biological processes including cell survival [4], proliferation [5], protein transport [6], angiogenesis [7], glucose and tyrosine metabolism [8], cholesterol and triglyceride synthesis [9]. Recent studies have implicated PRKCN in the development and progression of multiple solid tumors [4, 10]. However, its expression pattern and functionality in hematological malignancies including MM remains enigmatic.

Super‐enhancers (SEs) are large clusters of active typical enhancers densely occupied by master transcription factors and mediator complexes that evoke robust transcriptional activation [11]. SEs have been demonstrated to activate transcription of crucial genes such as IRF4 and c‐MYC in MM, supporting that SEs‐driven genes may be involved in myeloma disease progression and drug resistance [12, 13, 14]. Interestingly, by analyzing the publicly‐available chromatin immunoprecipitation sequencing (ChIP‐seq) datasets from previous studies [13, 15], PRKCN appears to be a potential SE‐associated gene in MM, yet whether it is really controlled by SE remains unknown.

In this study, we identify PRKCN as a SE‐driven gene abundantly expressed in MM and associated with poor prognosis. PRKCN is directly modulated by NF‐κB signaling intrinsically existing or exogenously provoked via SE binding. PRKCN promotes cell growth and confers resistance to standard therapeutic agents by orchestrating mTOR‐IRF4 signaling. Unexpectedly, PRKCN relies on activation loop phosphorylation rather than intrinsic kinase activity to exert its impact. Moreover, PRKCN disruption triggers activation of interferon (IFN) signaling partly through IRF4 downregulation and increases the susceptibility to IFN treatment. Intriguingly, IRF4 reciprocally induces PRKCN transcription. Lastly, an orally bioavailable PRKCN inhibitor CRT0066101 hinders PRKCN expression and activation loop phosphorylation, impairs mTOR‐IRF4 signaling and exhibits potent anti‐MM efficacy both in vitro and in vivo. Our study provides a rationale for further development and translation of therapeutic strategies targeting PRKCN in MM.

## 2 Results

### 2.1 PRKCN is Aberrantly Expressed in MM and Driven by a Super‐Enhancer (SE)

**FIGURE 1:**
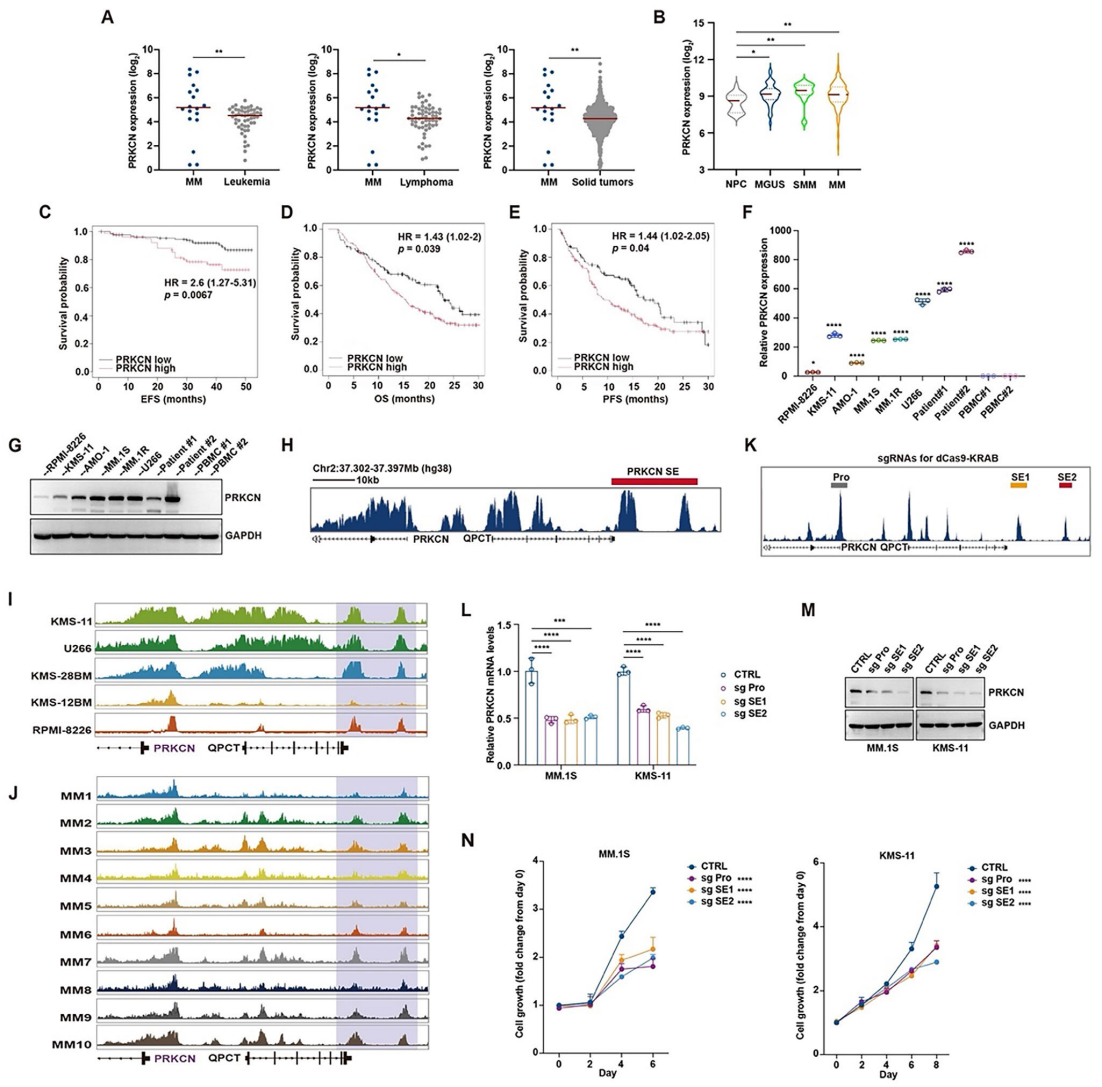
High PRKCN expression is correlated with adverse prognosis and driven by a super‐enhancer (SE) in MM. (A) Dot‐plot showing PRKCN expression in human MM (*n* = 18) vs. leukemia (*n* = 52), lymphoma (*n* = 59) and solid tumor cell lines (*n* = 979) derived from the DepMap portal. The middle line represents the median. Mann–Whitney test was performed. (B) Violin plot comparing PRKCN expressions in normal plasma cells (NPC, *n* = 22), monoclonal gammopathy of undetermined significance (MGUS, *n *= 44), smoldering multiple myeloma (SMM, *n* = 12) and MM patient samples (MM, *n* = 559) using GSE5900 and GSE2658 datasets. The middle line represents the median. Kruskal–Wallis test was performed. (C) Reduced event free survival (EFS) of newly diagnosed MM patients harboring high PRKCN expression defined using optimal cut points from GSE24080 dataset. (D,E) Shortened overall survival (OS) and progression‐free survival (PFS) of relapsed and/or refractory MM patients harboring high PRKCN expression using optimal cut points from APEX dataset (GSE9782). (F,G) PRKCN mRNA and protein levels were detected in a panel of MM cell lines, purified CD138^+^ primary MM cells and normal PBMCs by RT‐qPCR and immunoblot analysis, respectively. For RT‐qPCR, data are presented as mean ± SD. One‐way ANOVA with Dunnett's post hoc test was performed, *n* = 3. (H) The occupancy of H3K27ac at PRKCN‐associated SE region in MM.1S cells was visualized by UCSC Genome Browser. (I,J) The enrichment of H3K27ac at PRKCN‐associated SE region was visualized using UCSC Genome Browser in the additional MM cell lines (I) and patient‐derived MM samples (J) in GSE145938 dataset. (K) Schematic illustration of three sites targeted by dCas9‐KRAB CRISPRi at the open chromatin region of PRKCN SE. (L–N) PRKCN mRNA (L) and protein (M) expression, as well as cell viability (N) were measured in MM.1S and KMS‐11 cells expressing lentiviral sgRNAs with dCas9‐KRAB CRISPRi targeting SE1, SE2 or Pro (promoter) regions of PRKCN SE. For RT‐qPCR, data are presented as mean ± SD, and one‐way ANOVA with Dunnett's post hoc test was performed, *n* = 3. For cell viability assay, data are presented as mean ± SD, and two‐way ANOVA with Tukey's post hoc test was performed, *n* = 3.

We first interrogated the expression pattern of PRKCN using publicly available datasets. PRKCN transcripts were elevated in MM vs. other tumor cell lines including leukemia, lymphoma and solid tumor cells (Figure 1A; Figure S1). Coincidentally, PRKCN exhibited lineage‐specific enrichment in myeloma cells (Figure S2), suggesting a potential selective dependency in this malignancy, despite that a Chronos dependency score of ‐0.2 merely indicates a relatively weak dependency. Moreover, PRKCN mRNA expression levels were increased at the stages of MGUS, SMM and MM as compared to normal plasma cells (Figure 1B; Figure S3A,B). By analyzing patient outcomes, heightened PRKCN expression was associated with inferior EFS in newly diagnosed patients (Figure 1C), and also correlated with shorter OS and PFS in relapsed/refractory patients (Figure 1D,E), while such correlation with poor prognosis did not exist for PRKD1 or PRKD2 (Figure S3C–E). Additionally, we verified that PRKCN mRNA and protein expression were easily detected in multiple human MM cells lines (HMCLs) and primary patient samples yet becoming negligible in normal peripheral blood mononuclear cells (PBMCs) (Figure 1F,G).

SE plays a critical role in the transcriptional reprogramming for MM [12, 13]. By analyzing the ChIP‐seq dataset related to a previous study exploring the potential SE‐associated genes in myeloma cell line MM.1S, PRKCN was among 15 protein kinase‐encoding genes potentially associated with SEs (Figure 1H; Figure S4A) [13]. Moreover, PRKCN‐associated SE was visualized in several other MM cell lines and primary MM samples by analyzing another ChIP‐seq dataset (Figure 1I,J) [15]. To ascertain whether PRKCN transcription was controlled by SE, we examined the effects of JQ1, a small‐molecule inhibitor blocking BRD4 binding to H3K27ac [16], on SE activity and PRKCN expression. Occupancy of BRD4 and MED1 at the SE locus (Figure S4B), as well as PRKCN expression in HMCLs and primary MM cells declined following JQ1 treatment (Figure S4C,D), indicating that PRKCN was susceptible to transcriptional inhibition, a general feature of SE‐driven genes. Then we exploited dCas9‐KRAB‐based CRISPR interference (CRISPRi) technology, in which three single guide RNAs (sgRNA) were designed to specifically target the PRKCN promoter as a positive control and two constituent enhancers (SE1 and SE2) within the PRKCN‐SE (Figure 1K; Figure S4E), to investigate the impact of promoter and SE perturbation on PRKCN transcription and cell growth. CRISPRi perturbation of PRKCN SE significantly down‐regulated PRKCN expression (Figure 1L,M) and diminished cell growth (Figure 1N), reflecting transcriptional dependency of PRKCN driven by SE in MM.

### 2.2 PRKCN is Transactivated by Cell‐Intrinsic and Cell‐Extrinsic NF‐κB Signaling

**FIGURE 2:**
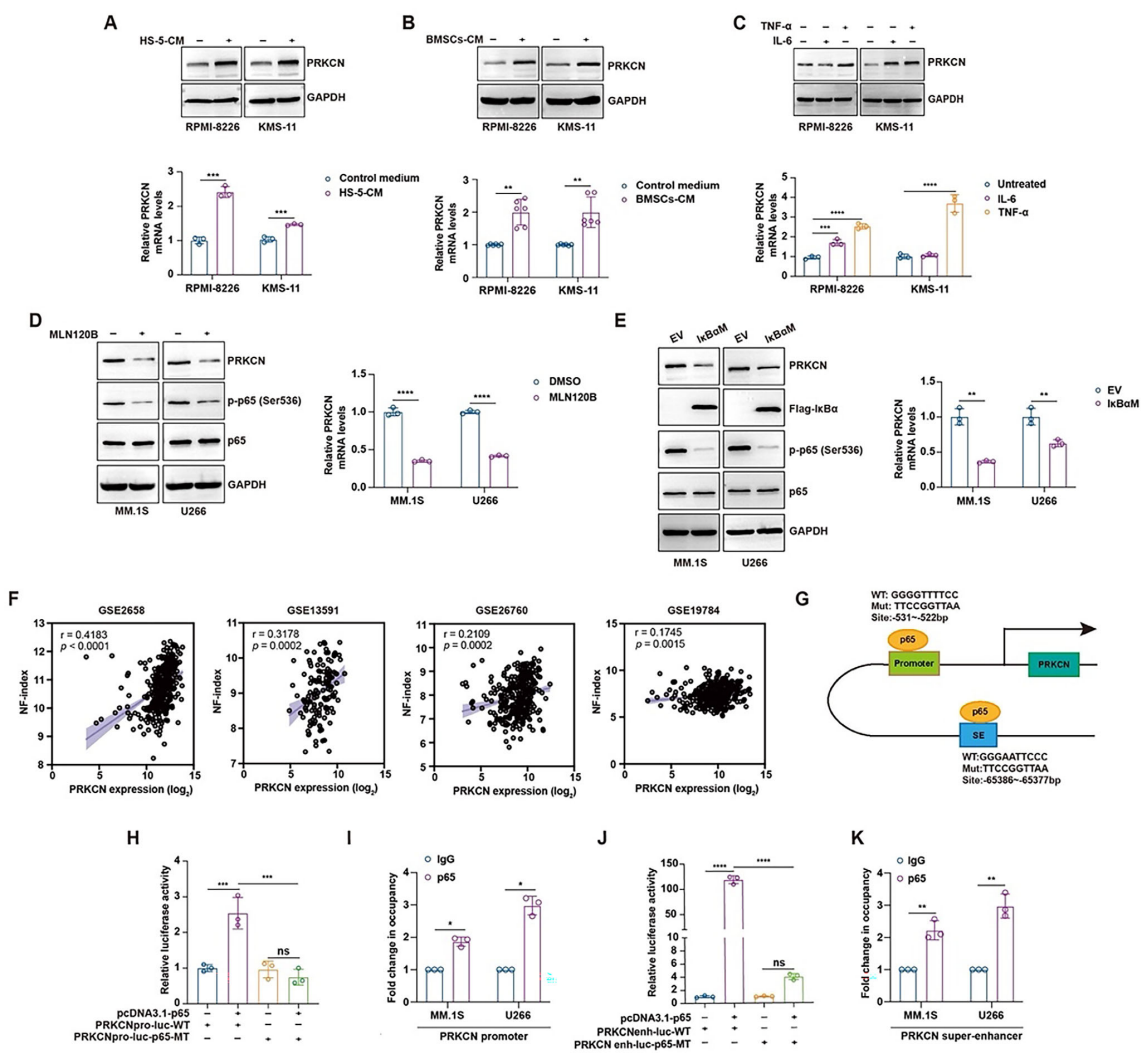
PRKCN is transactivated by NF‐κB signaling pathway existing intrinsically or provoked by exogenous stimuli in MM cells. (A‐B) RPMI‐8226 or KMS‐11 cells were exposed to the conditioned medium from HS‐5 (HS‐5‐CM) or primary BMSCs (BMSCs‐CM), and PRKCN protein and mRNA expression levels were detected by immunoblot and RT‐qPCR analysis, respectively. For RT‐qPCR, data are presented as mean ± SD. Unpaired two‐tailed *t* test (A) or unpaired two‐tailed Welch' *t* test (B) was performed, *n* = 3 or 6. (C) RPMI‐8226 or KMS‐11 cells treated with TNF‐α or IL‐6 were subjected to immunoblot and RT‐qPCR analysis of PRKCN expression. For RT‐qPCR, data are presented as mean ± SD. One‐way ANOVA with Dunnett's post hoc test was performed, *n* = 3. (D) Following MLN120B treatment, MM.1S and U266 cells were analyzed for PRKCN, p65 and p‐p65 expressions by immunoblot analysis, and for PRKCN mRNA by RT‐qPCR analysis. (E) MM.1S and U266 cells were transduced with IKBαM lentiviruses, and immunoblot analysis was applied to detect expression of PRKCN, p65, p‐p65 and IKBα proteins, and RT‐qPCR was used to assess PRKCN mRNA expression. For RT‐qPCR (D, E), data are presented as mean ± SD. Unpaired two‐tailed *t* test or unpaired two‐tailed Welch' *t* test was performed, *n* = 3. (F) Spearman's correlation analysis of the expression of PRKCN mRNA with NF‐κB index in MM patients using multiple GEO datasets. (G) Diagram depicting PRKCN proximal promoter and super‐enhancer with potential p65 binding sites. The corresponding mutants for the binding site were constructed. (H) Cell lysates from HEK293T cells co‐transfected with PRKCNpro‐luc‐WT or PRKCNpro‐luc‐p65‐MT together with pcDNA3.1‐p65 plasmids were prepared for luciferase activity measurement. Data is presented as mean ± SD. One‐way ANOVA with Tukey's post hoc test was performed, *n* = 3. (I) ChIP‐qPCR analysis showing occupancy of p65 at PRKCN proximal promoter in MM.1S and U266 cells. Data are presented as mean ± SD. Unpaired two‐tailed Welch' *t* test was performed, *n* = 3. (J) Cell lysates from HEK293T cells co‐transfected with indicated plasmids were prepared for luciferase activity measurement. Data is presented as mean ± SD. One‐way ANOVA with Tukey's post hoc test was performed, *n* = 3. (K) ChIP‐qPCR analysis showing occupancy of p65 at PRKCN SE region in MM.1S and U266 cells. Data are presented as mean ± SD. Unpaired two‐tailed Welch' *t* test was performed, *n* = 3.

The pro‐inflammatory cytokines secreted by bone marrow stromal cells (BMSCs) in the bone marrow microenvironment, such as IL‐6 and TNF‐α, contributed to the transcriptional upregulation of some oncogenic drivers in MM [17, 18, 19], motivating us to investigate whether the similar phenomenon occurred to PRKCN. Exposure to the conditioned media (CM) from HS‐5 stromal cell line or MM patient‐derived BMSCs triggered PRKCN expression in RPMI‐8226 and KMS‐11 cells (Figure 2A,B). Of note, TNF‐α significantly increased both mRNA and protein levels of PRKCN in HMCLs, while IL‐6 displayed asynchronous or divergent influence on the mRNA and protein expression of PRKCN (Figure 2C), pinpointing that TNF‐α might serve as the major cytokine responsible for transcriptional regulation of PRKCN. Furthermore, PRKCN upregulation by TNF‐α, HS‐5‐CM or BMSCs‐CM exposure was remarkably blunted upon blockade of canonical NF‐κB signaling with IκB kinase (IKK) β inhibitor MLN120B (Figure S5A–C), corroborating involvement of canonical NF‐κB signaling.

We next assessed whether PRKCN was modulated by intrinsic NF‐κB signaling frequently activated in MM due to diverse genetic mutations [20]. Blockade of canonical NF‐κB signaling by different approaches, including MLN120B treatment, ectopic expression of IκBαM as the super‐repressor [21], or alternatively knockout of p65 (RELA) significantly impaired PRKCN expression (Figure 2D,E; Figure S5D). In stark contrast, perturbation of non‐classical NF‐κB signaling with NIK inhibitor B022 failed to influence PRKCN expression (Figure S5E), suggesting that PRKCN was solely influenced by canonical NF‐κB signaling. Parallelly, by analyzing multiple GEO datasets, we noticed the strong positive correlation between PRKCN expression levels and the well‐defined NF‐κB index in primary MM cells (Figure 2F; Figure S6).

Inspection of the proximal promoter revealed existence of a putative NF‐κB/p65‐binding site (Figure 2G). Luciferase reporter assay showed that this site was indispensable for transactivation of PRKCN promoter reporter by p65, and interaction between p65 and PRKCN promoter was validated by ChIP‐qPCR assay (Figure 2H,I). Occupation of proximal PRKCN promoter by p65 also occurred in several other cell types, illustrating a universal phenomenon (Figure S7A). Moreover, analysis of the publicly available ChIP‐seq databases showed that p65 may bind the 800‐bp fragment at PRKCN SE locus (SE2), harboring a potential p65‐binding site (Figure 2G; Figure S7B), which was critical for p65 transactivating a minimal promoter in conjunction with the enhancer locus as determined by luciferase reporter assay (Figure 2J), and interaction of p65 with this locus was verified by ChIP‐qPCR assay in HMCLs (Figure 2K). The above findings suggested that PRKCN was transactivated by NF‐κB signaling through both promoter and SE binding. Then we asked whether PRKCN mediated cell growth under NF‐κB pathway. MLN120B‐induced cell growth inhibition was partially negated by ectopic PRKCN expression (Figure S8), favoring PRKCN as a novel downstream effector of NF‐κB signaling.

### 2.3 PRKCN Accelerates Cellular Growth In Vitro and In Vivo, and Confers Resistance to Standard Therapeutic Agents

**FIGURE 3:**
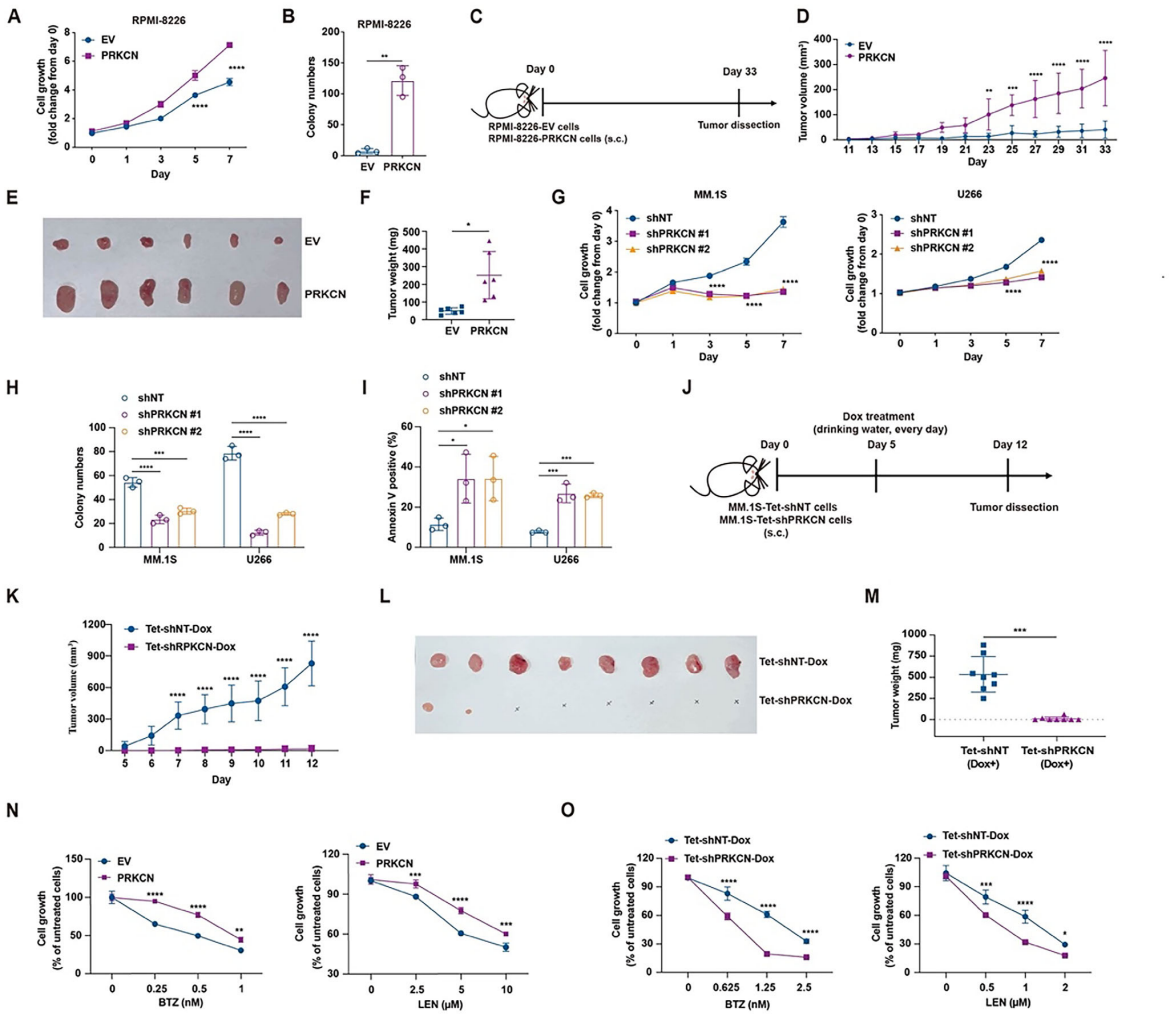
PRKCN augments cell growth and survival in vitro and in vivo and confers resistance to standard therapeutic agents. (A) RPMI‐8226 cells were transduced with PRKCN or Venus lentivirus (EV). After three days of infection, which was designated as day 0, cells were reseeded, and cell viability was detected on days 0, 1, 3, 5, and 7 by CCK‐8 assay. Data is presented as mean ± SD. Two‐way ANOVA with Šídák's post hoc test was performed, *n* = 3. (B) RPMI‐8226 cells transduced with PRKCN or EV were cultured in a medium‐agarose mixture for 21 days for assessing colony formation capacity. Data are presented as mean ± SD. Unpaired two‐tailed *t* test was performed, *n* = 3. (C) Schematic representation of RPMI‐8226 xenograft mouse model with the indicated cells injected subcutaneously into NCG mice. (D) The changes in tumor volume over the time were monitored. Data is presented as mean ± SD. Two‐way ANOVA with Šídák's post hoc test was performed, *n* = 6. (E,F) Tumors were dissected and photographed, and tumor weight was qualified at the end point. Data are presented as mean ± SD (F). Unpaired two‐tailed Welch's *t* test was performed, *n* = 6. (G) MM.1S and U266 cells were transduced with shPRKCN or shscramble lentivirus (negative control, NT). After 3 days of infection, which was designated as day 0, cells were reseeded, and cell viability was detected on indicted time points by the CCK‐8 assay. Data is presented as mean ± SD. Two‐way ANOVA with Tukey's post hoc test was performed, *n* = 3. (H) Infected cells were cultured in the presence of agarose for 21 days, and the number of colonies was quantified. (I) Following infection for 5 days, apoptotic cells were measured by Annexin V staining. Data are presented as mean ± SD (H,I). One‐way ANOVA with Dunnett's post hoc test was performed, *n *= 3. (J) Schematic representation of MM.1S xenograft mouse model with inducible PRKCN knockdown. MM.1S‐Tet‐shNT or MM.1S‐Tet‐shPRKCN cells were injected subcutaneously into NCG mice. 5 days after implantation, the mice received doxycycline (Dox, l mg/ml in 5% sucrose) via drinking water for the duration of study. (K) Tumor volumes from each group were monitored over time. Data are presented as mean ± SD. Two‐way ANOVA with Šídák's post hoc test was performed, *n* = 8. (L,M) Tumors were photographed (L), and tumor weight was quantified at the endpoint (M). Data is presented as mean ± SD (M). Unpaired two‐tailed Welch's *t* test was performed, *n* = 8. (N) Cell viability was measured by CCK8 assay in RPMI‐8226‐EV or RPMI‐8226‐PRKCN cells treated with or without bortezomib (BTZ) or lenalidomide (LEN) for 24 h. (O) MM.1S‐Tet‐shNT and MM.1S‐Tet‐shPRKCN cells were cultured in the presence of Dox (1 µg/mL) for 3 days, followed by treatment with BTZ or LEN for another day, and cell viability was assessed by CCK8 assay. Data is presented as mean ± SD (N,O). Two‐way ANOVA with Šídák's post hoc test was performed, *n* = 3.

To evaluate the phenotypical traits of PRKCN, we genetically manipulated PRKCN expression in HMCLs. PRKCN overexpression augmented cell proliferation and clonogenicity (Figure 3A,B; Figure S9A,B), and accelerated tumorigenicity in immunodeficient mice (Figure 3C–F). Conversely, constitutive PRKCN knockdown caused obvious growth retarding, cell cycle arrest and reduced clonogenicity (Figure 3G,H; Figure S9C–E), and triggered remarkable apoptosis (Figure 3I; Figure S9F). We also generated MM.1S cells harboring the inducible knockdown construct Tet‐shPRKCN in which shPRKCN expression was induced by doxycycline (Dox) treatment. Similar to constitutive knockdown, Dox‐triggered PRKCN knockdown impaired cell growth and elicited apparent apoptosis (Figure S10A–F). Following inoculation into immunodeficient mice, the growth of tumors developed by MM.1S‐Tet‐shPRKCN cells, as well as MM.1S‐Tet‐shNT‐FFL and MM.1S‐Tet‐shPRKCN‐FFL cells was remarkably hampered by Dox treatment (Figure 3J–M; Figure S10G–L).

We then investigated whether PRKCN influenced MM cell sensitivity to the standard therapeutic agents including bortezomib (BTZ) and lenalidomide (LEN). In RPMI‐8226 cells, BTZ or LEN treatment evoked growth inhibition, which could be partly abrogated by PRKCN overexpression (Figure 3N). On the contrary, PRKCN depletion intensified MM.1S cell growth inhibition induced by BTZ or LEN treatment (Figure 3O), suggesting that loss of PRKCN augmented the sensitivity to either treatment. Moreover, the isogenic BTZ‐ or LEN‐ resistant MM.1S cells generated by gradient drug exposure remained vulnerable to PRKCN depletion (Figure S11).

### 2.4 PRKCN Harnesses IRF4 to Promote Cell Growth and is Reciprocally Regulated by IRF4

**FIGURE 4:**
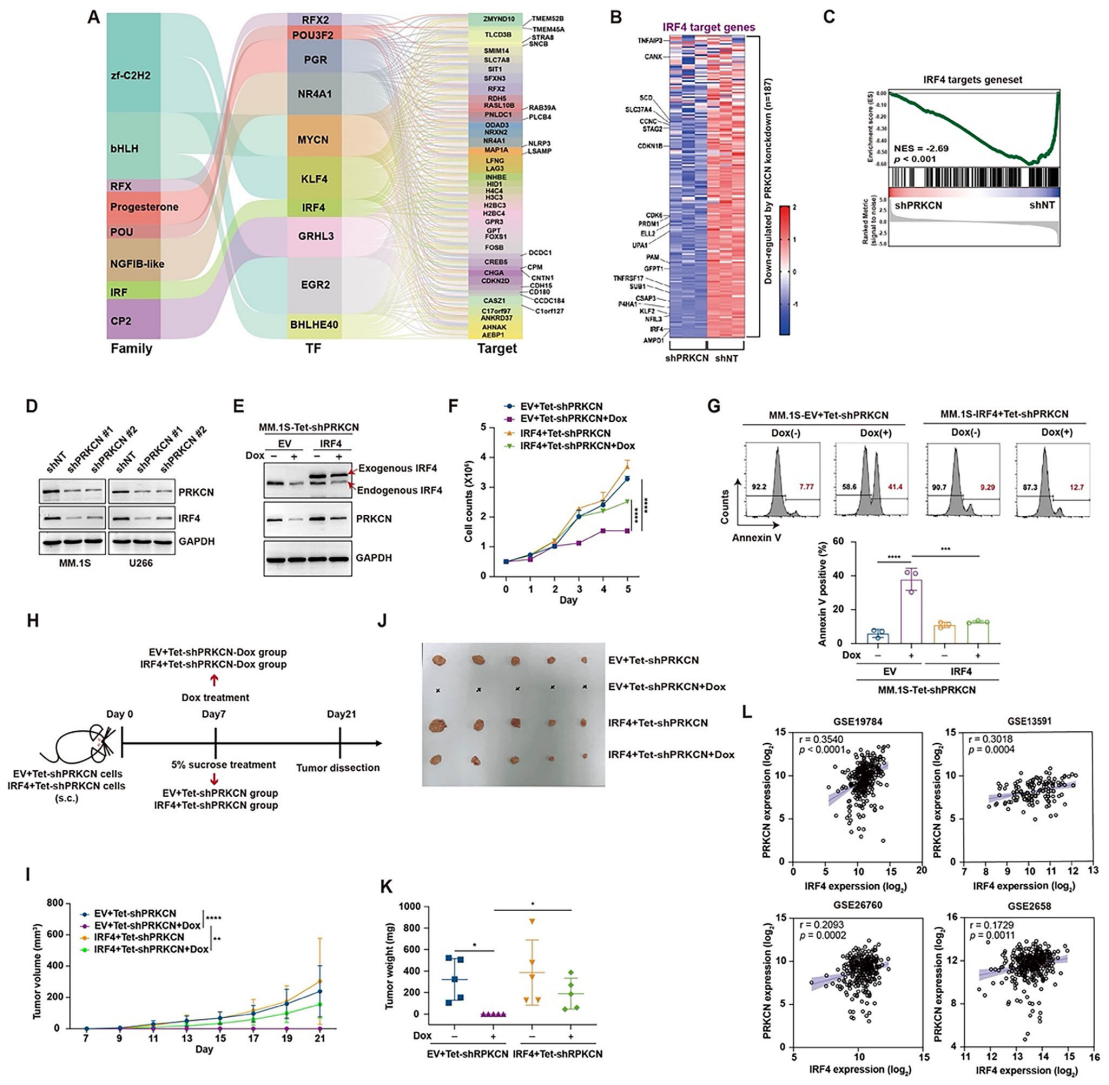
PRKCN harnesses IRF4 to promote cell growth and is reciprocally regulated by IRF4. (A) Sankey diagram indicating the top 10 transcription factors differentially expressed between the shNT group and shPRKCN group with the correspondence between transcription factors and putative targets. (B) Heat‐map view of changes in the expression of IRF4 target genes (genes significantly downregulated by IRF4 knockdown) in shPRKCN group vs. shNT group. (C) Significant correlation between the genes downregulated by PRKCN knockdown and those by IRF4 knockdown. The genes significantly downregulated by IRF4 knockdown were used as a gene set for GSEA. (D) Immunoblot analysis of PRKCN and IRF4 expression in MM.1S and U266 cells subjected to constitutive PRKCN knockdown. (E) Immunoblot analysis of PRKCN and IRF4 expression in MM.1S‐Tet‐shPRKCN cells transduced with Venus or IRF4‐overexpressing lentiviruses in the presence or absence of Dox for 3 days. (F) MM.1S‐Tet‐shPRKCN cells transduced with IRF4 lentiviruses were cultured in the presence or absence of Dox and cell growth was measured. Data is presented as mean ± SD. Two‐way ANOVA with Tukey's post hoc test was performed, *n* = 3. (G) MM.1S‐Tet‐shPRKCN cells transduced with IRF4 lentiviruses were incubated with Dox for 5 days, and apoptosis was assessed by Annexin V staining. Data is presented as mean ± SD. One‐way ANOVA with Tukey's post hoc test was performed, *n* = 3. (H) Schematic representation of the in vivo IRF4 “rescue” experiment. MM.1S‐EV+Tet‐shPRKCN or MM.1S‐IRF4+Tet‐shPRKCN cells were injected subcutaneously into NCG mice. One week after implantation, the mice received Dox (l mg/ml in 5% sucrose) or vehicle (5% sucrose) via drinking water for the duration of the study. (I) Tumor volumes from each group were monitored over time. Data is presented as mean ± SD. One‐way ANOVA with Tukey's post hoc test was performed, *n* = 5. (J,K) Mice were sacrificed after 21 days, and tumors were harvested and photographed (J), and tumor weight was quantified (K). Data are presented as mean ± SD. Unpaired two‐tailed Welch' *t* test Data was performed, *n* = 5. (L) Spearman's correlation analysis of the expression of PRKCN with IRF4 in MM patients using multiple GEO datasets.

To probe the mechanistic role of PRKCN in MM, we conducted RNA‐seq analysis to profile the transcriptomic alterations resulting from depletion of PRKCN in MM.1S cells, and identified up‐regulated 2084 genes and 2783 down‐regulated genes (Figure S12A). Transcription factor‐focused analyses identified the top 10 differentially expressed transcription factors, including up‐regulated GRHL3 and PGR, and down‐regulated RFX2, POU3F2, NR4A1, MYCN, KLF4, IRF4, EGR2, and BHLHE40 (Figure 4A).

We focused on interferon regulatory factor 4 (IRF4) since it was a master regulator of MM cell growth and survival [22, 23, 24]. To explore whether PRKCN regulated a batch of IRF4 target genes identified by Shaffer AL et al. [22], we took these genes as a gene set for gene set enrichment analysis (GSEA). The target gene set of IRF4 was significantly inhibited following PRKCN knockdown in MM.1S cells (Figure 4B,C). We validated that knockdown or knockout of PRKCN reduced expression of IRF4 and its downstream targets, including BCMA (TNFRSF17) and Kruppel‐like factor 2 (KLF2) in HMCLs (Figure 4D; Figure S12B–H) [22, 25], while PRKCN overexpression elicited the opposite effect (Figure S12I–L). Moreover, regulation of IRF4 and its downstream targets by PRKCN was faithfully replicated in drug‐resistant cells (Figure S13) and two xenograft models (Figure S14).

To address whether PRKCN functioned through IRF4, we silenced IRF4 in PRKCN‐overexpressing RPMI‐8226 cells and observed that PRKCN‐driven cell growth was largely abolished (Figure S15A–C). We further performed rescue experiments by ectopically expressing IRF4 in MM.1S‐Tet‐shPRKCN cells. IRF4 overexpression remarkably counteracted down‐regulation of PRKCN, BCMA and KLF2, cell growth suppression and apoptosis triggered by Dox treatment (Figure 4E–G; Figure S15D). Moreover, Dox‐induced PRKCN depletion in the control MM.1S‐EV+Tet‐shPRKCN cells caused complete xenograft regression, while the xenografts developed from MM.1S‐IRF4+Tet‐shPRKCN cells remained largely refractory to Dox treatment (Figure 4H–K). Therefore, PRKCN co‐opted IRF4 to drive MM cell growth. Since IRF4 can form autoregulatory feedback loops with some oncogenic drivers, such as c‐MYC to promote malignant transformation [25, 26], we queried whether this may occur between IRF4 with PRKCN. Indeed, IRF4 knockdown or knockout blunted PRKCN expression (Figure S16A–C). Conversely, IRF4 overexpression was sufficient to augment PRKCN expression, while overexpression of its mutant lacking DNA binding domain failed (Figure S16D–F), prompting us to examine whether IRF4 might transactivate PRKCN via direct promoter or SE binding. Analysis of the publicly available ChIP‐seq dataset SRX327760 indicated that there existed several IRF4 binding peaks in both the proximal promoter and distant SE region of PRKCN in MM.1S cells (Figure S16G). Luciferase reporter assay coupled with ChIP‐qPCR analyses showed that IRF4 induced transactivation of PRKCN predominantly via binding the distant SE locus in MM.1S and U266 cells (Figure S16H–J), raising PRKCN as a direct transcriptional target of IRF4. Therefore, PRKCN coordinated with IRF4 by forming a positive regulatory loop connecting them. In alignment with this, PRKCN expression levels were positively correlated with those of IRF4 in primary samples (Figure 4L), and IRF4 target gene set was also enriched in MM patients with abundant PRKCN expression in multiple datasets (Figure S17).

### 2.5 PRKCN Tunes IRF4 Transcription by Activating mTORC1/C2 Pathways

**FIGURE 5:**
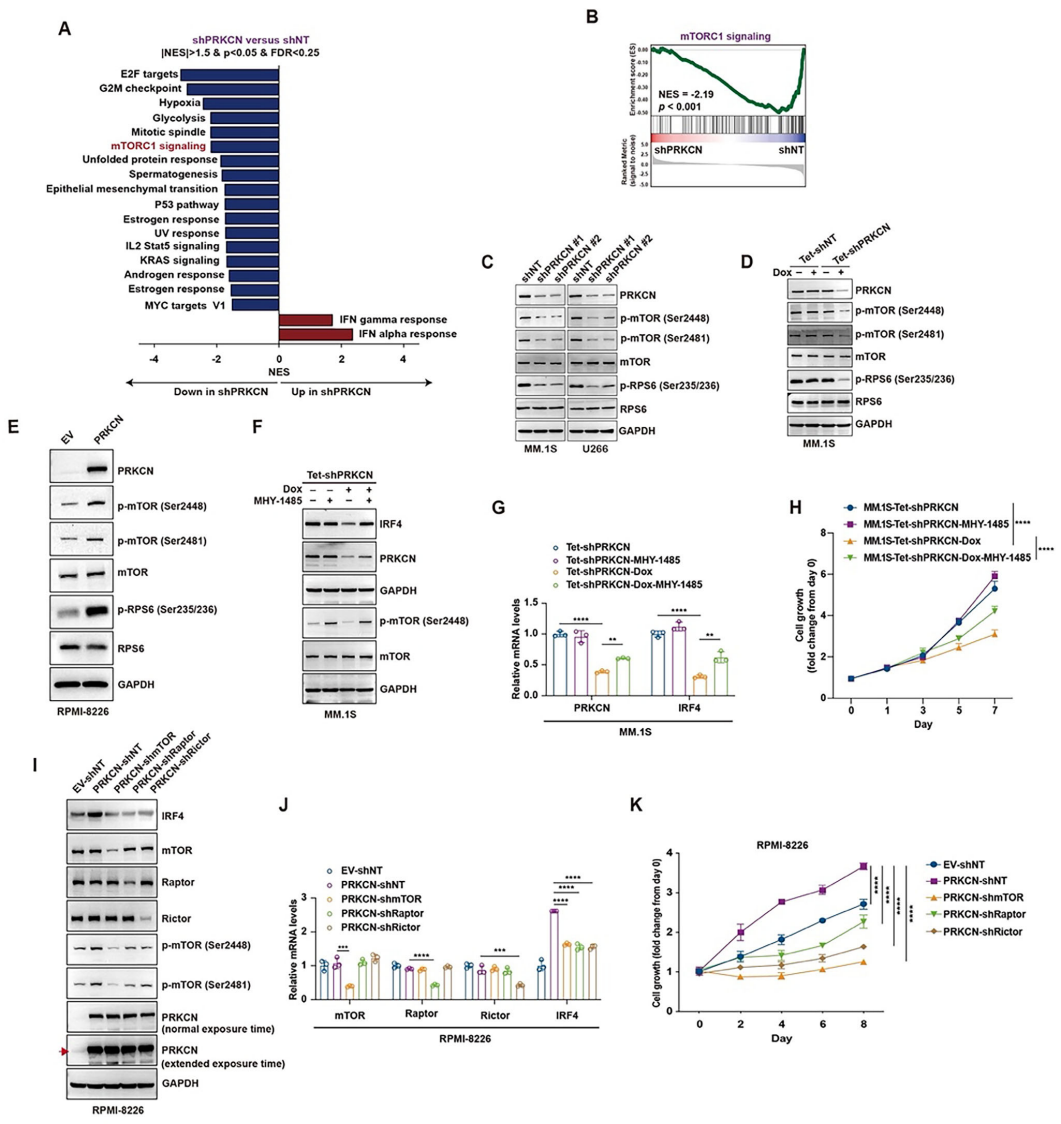
PRKCN modulates IRF4 expressions by activating mTORC1/C2 signaling pathways. (A) GSEA plot of upregulated and downregulated Hallmark pathways in PRKCN knockdown cells (shPRKCN) vs. control cells (shNT) by RNA‐seq as ordered by normalized enrichment score (NES). (B) GSEA plot of Hallmark mTORC1 signaling in shPRKCN vs. shNT cells. (C–E) Immunoblot analysis of PRKCN, mTOR, p‐mTOR, RPS6, p‐RPS6 in MM.1S or U266 cells subjected to constitutive PRKCN knockdown (C), MM.1S‐Tet‐shPRKCN cells treated with Dox (D), or RPMI‐8226 cells transduced with lentiviruses encoding PRKCN (E). (F–H) MM.1S‐Tet‐shPRKCN cells were exposed to Dox in the presence or absence of MHY‐1485, and analyzed for IRF4, PRKCN, mTOR and p‐mTOR protein expression by immunoblot analysis (F), for PRKCN and IRF4 mRNA expression by RT‐qPCR analysis (G), and for cell viability by the CCK‐8 assay (H). (I‐K) RPMI‐8226‐PRKCN cells were transduced with specific shRNAs targeting mTOR, Raptor or Rictor, and analyzed for protein expression of mTOR, p‐mTOR, Raptor, Rictor and IRF4 by immunoblot analysis (I), for mRNA expression of mTOR, Raptor, Rictor and IRF4 by RT‐qPCR analysis (J), and for cell viability by CCK‐8 assay (K). For RT‐qPCR, data are presented as mean ± SD (G,J). One‐way ANOVA with Tukey's post hoc test was performed, *n* = 3. For cell viability, data are presented as mean ± SD (H,K). Two‐way ANOVA with Tukey's post hoc test was performed, *n* = 3.

To elucidate the potential mechanism whereby PRKCN modulated IRF4, we performed gene set enrichment analysis (GSEA) on RNA‐seq data and found 17 hallmark signaling pathways were significantly inhibited following PRKCN depletion, including E2F targets, G2M checkpoint and mTORC1 signaling pathway (Figure 5A,B). Given that aberrant mTOR activation is implicated in MM by promoting cell survival, growth, and chemotherapy resistance [27, 28, 29], and also mTORC1 signaling components were primarily identified as the top‐ranked regulators of IRF4 in myeloma cell lines by CRISPR/Cas9 screening [23], we hypothesized that PRKCN may regulate IRF4 expression primarily by activating mTOR signaling pathway.

PRKCN depletion triggered downregulation of p‐mTOR (Ser2448), p‐RPS6 (Ser235/236) and p‐mTOR (Ser2481) in HMCLs and the BTZ‐ or LEN‐resistant MM.1S cells, while PRKCN overexpression augmented their expression in HMCLs (Figure 5C–E; Figure S13A), corroborating simultaneous activation of mTORC1 and mTORC2 (mTORC1/C2) pathways by PRKCN. Consistently, mTOR signaling is positively enriched in PRKCN‐high MM patients in multiple datasets (Figure S18A). By individually silencing the key components of mTORC1/C2 complexes, including mTOR, Raptor, Rictor and RPS6, we confirmed that mTORC1/C2 signaling modulated IRF4 signaling axis in HMCLs (Figure S18B–I). To interrogate whether activated mTORC1 or mTORC2 signaling was harnessed by PRKCN to sustain IRF4 expression and cell growth, we treated MM.1S‐Tet‐shPRKCN cells with MHY‐1485, the agonist of mTORC1 signaling. IRF4 downregulation and cell growth arrest induced by Dox treatment was partially restored by MHY‐1485 treatment (Figure 5F–H). Meanwhile, shRNA‐mediated disruption of mTORC1/C2 signaling pathways obviously blunted PRKCN‐induced IRF4 upregulation and cell growth enhancement (Figure 5I–K). Taken together, PRKCN boosted IRF4 expression and MM cell growth by orchestrating mTORC1/C2 signaling pathways.

### 2.6 PRKCN Activates mTORC1/C2‐IRF4 Axis Independently of Kinase Activity but Requiring Activation Loop Phosphorylation

**FIGURE 6:**
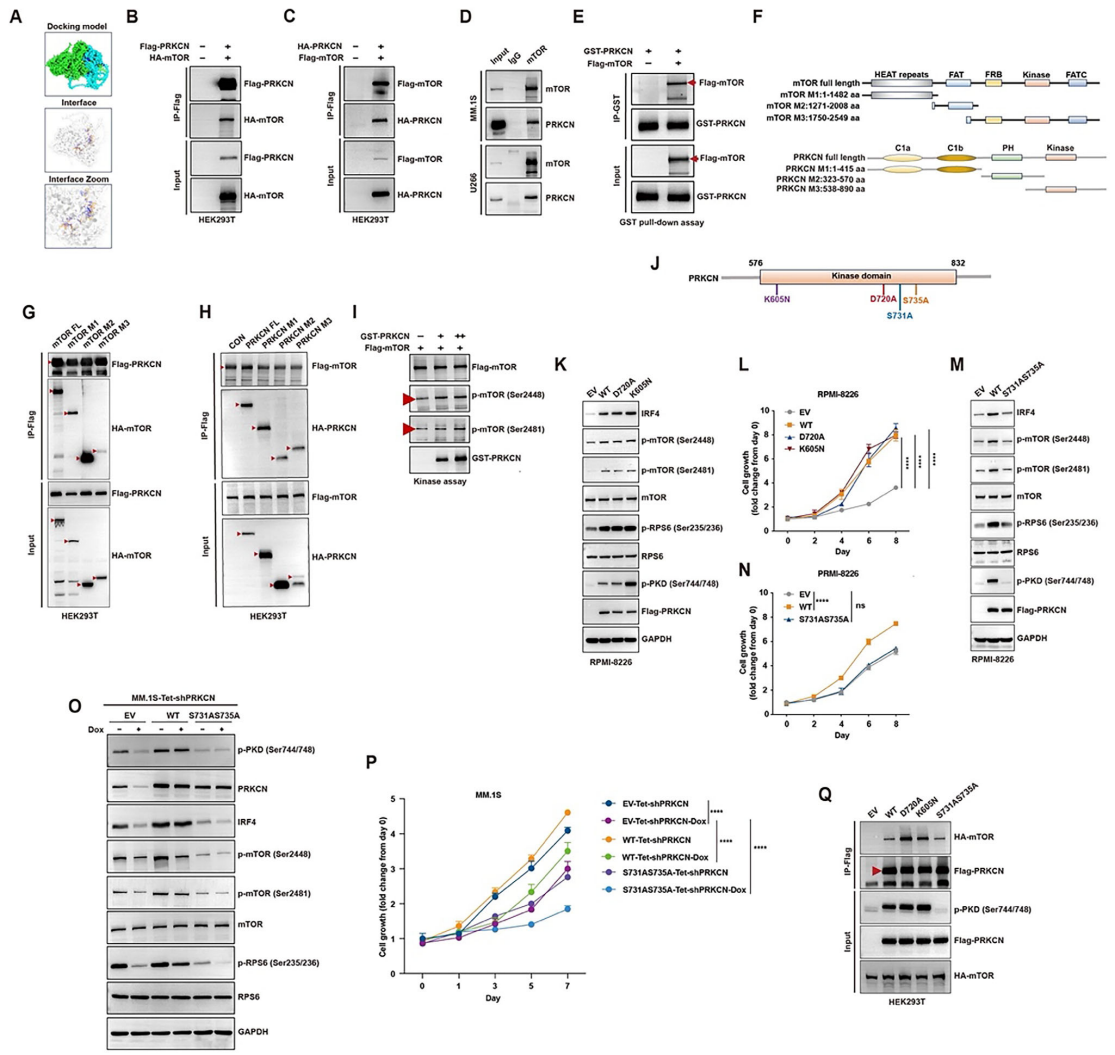
PRKCN interacts with mTOR to activate mTORC1/C2‐IRF4 axis independently of kinase activity but requiring activation loop phosphorylation. (A) Prediction of the interaction between mTOR and PRKCN by molecular docking simulation analysis. (B) Immunoblot analysis showing the presence of mTOR in anti‐Flag immunoprecipitation from HEK293T cells co‐transfected with Flag‐PRKCN and HA‐mTOR. (C) Immunoblot analysis indicating the presence of PRKCN in anti‐Flag immunoprecipitation from HEK293T cells co‐expressing Flag‐mTOR and HA‐ PRKCN. (D) Immunoblot analysis showing the presence of PRKCN in anti‐mTOR immunoprecipitation from MM.1S and U266 cells. (E) Immunoblots showing that recombinant GST‐PRKCN pulled down recombinant Flag‐mTOR in a cell‐free system. (F) Cartoons showing the truncated mutants of mTOR (upper) and PRKCN (lower). (G) Each HA‐mTOR mutant was co‐expressed with Flag‐PRKCN in HEK293T cells and subjected to co‐immunoprecipitation analysis. (H) Each HA‐PRKCN mutant was co‐expressed with Flag‐mTOR in HEK293T cells and subjected to co‐immunoprecipitation analysis. (I) The cell‐free kinase assay was used to evaluate the possibility of PRKCN directly phosphorylating mTOR at Ser2448 and Ser2481 sites. (J) Schematic diagram of wild‐type PRKCN and three different forms of kinase‐inactive mutants including D720A, K605N and S731AS735A. (K‐L) RPMI‐8226 cells transduced with lentiviruses encoding wild‐type PRKCN (WT), D720A or K605N mutant were analyzed for IRF4, mTOR, p‐mTOR, RPS6, p‐RPS6, PRKCN and p‐PKD by immunoblot analysis (K), and for proliferation by CCK‐8 assay (L). (M‐N) RPMI‐8226 cells transduced with empty vector (EV), wild‐type PRKCN (WT) or S731AS735A mutant were analyzed for IRF4, mTOR, p‐mTOR, RPS6, p‐RPS6, PRKCN and p‐PKD by immunoblot analysis (M), and for proliferation by CCK‐8 assay (N). (O,P) MM.1S‐Tet‐shPRKCN cells transduced with lentiviruses encoding shRNA‐resistant wild‐type PRKCN (WT) or S731AS735A mutant in the presence or absence of Dox were analyzed for IRF4, mTOR, p‐mTOR, RPS6, p‐RPS6, PRKCN and p‐PKD by immunoblot analysis (O), and for proliferation by CCK‐8 assay (P). (Q) Immunoblot analysis showing the presence of mTOR in anti‐Flag immunoprecipitation from HEK293T cells co‐transfected with Flag tagged wild‐type PRKCN, D720A, K605N or S731AS735A mutant and HA‐mTOR. Data are presented as mean ± SD (L,N,P). Two‐way ANOVA with Tukey's post hoc test was performed, *n* = 3.

We next sought to dissect the molecular basis for activation of mTORC1/C2 signaling pathways. Since PRKCN didn't promote p‐AKT expression in HMCLs (Figure S19), it cannot augment mTOR signaling through activating PI3K‐AKT axis. Then we interrogated whether PRKCN may function by interacting with the core mTOR protein shared by mTORC1 and mTORC2 complexes. We simulated 3D structure of PRKCN and mTOR, and protein docking showed that PRKCN and mTOR interacted extensively, displaying the characteristics of widespread and multi‐domain binding (Figure 6A; Figure S20). Confocal imaging showed that PRKCN colocalized with mTOR predominantly in the cytoplasm (Figure S21A). Co‐immunoprecipitation (Co‐IP) experiments substantiated the interplay between exogenously expressed PRKCN and mTOR in HEK293T cells (Figure 6B,C). The physical interaction between endogenous PRKCN and mTOR was confirmed in HMCLs (Figure 6D). This association appeared to be direct, as glutathione S‐transferase (GST)‐tagged PRKCN protein precipitated Flag‐tagged mTOR in vitro (Figure 6E). To further elucidate the structural basis of the PRKCN‐mTOR association, we queried the capability of specific mTOR or PRKCN domains (Figure 6F; Figure S21B) to pull down wild‐type PRKCN or mTOR, respectively. The tested individual domains of mTOR interacted with PRKCN (Figure 6G; Figure S21C), and also each examined domain of PRKCN interacted with mTOR (Figure 6H), which was supported by molecular docking (Figure S20). The specific components of mTORC1 and mTORC2, Raptor and Rictor, also coimmunoprecipitated PRKCN (Figure S21D), suggesting that direct interaction with mTOR may render PRKCN association with mTORC1 and mTORC2 complexes. However, PRKCN overexpression failed to affect the amount of Raptor or Rictor coimmunoprecipitated with mTOR (Figure S21E,F), excluding the possibility that PRKCN may activate mTOR signaling through strengthening the interaction between mTOR with Raptor/Rictor. By in vitro kinase assay, Flag‐mTOR was efficiently phosphorylated at both Ser2448 and Ser2481 residues by GST‐PRKCN (Figure 6I), indicating that PRKCN may phosphorylate mTOR by virtue of intrinsic kinase activity.

To further ascertain whether PRKCN relied on its kinase activity to activate mTORC1/C2‐IRF4 axis in MM cells, we introduced RPMI‐8226 cells transfected with two different constructs expressing the catalytically‐inactive PRKCNs mutants, D720A and K605N (Figure 6J) [30, 31]. In accordance with the previous reports [30], both mutants retained intact phosphorylation of Ser731 and Ser735 in the activation loop alike to wild‐type PRKCN as detected by the anti‐p‐PKD (Ser744/748) antibody, which recognizes the phosphorylated state of equivalent serine within three PKD isoforms (Figure 6K). Intriguingly, enforced expression of either mutant efficiently increased expression of p‐mTOR (Ser2448), p‐mTOR (Ser2481), p‐PRS6 (Ser235/236) and IRF4, comparable to that induced by overexpression of wild‐type PRKCN (Figure 6K). Moreover, both kinase‐inactive PRKCNs promoted cell growth resembling wild‐type PRKCN (Figure 6L), hinting kinase activity‐independent action of PRKCN. The S731AS735A mutation at the activation loop of PRKCN can prevent activation loop phosphorylation and therefore abolish its kinase activity [32]. Interestingly, overexpression of this mutant neither enhanced mTOR‐IRF4 signaling, nor promoting cell growth (Figure 6M,N). Consistently, compromised mTORC1/C2‐IRF4 signaling and cell growth secondary to PRKCN knockdown were considerably rescued by add‐back of wild‐type PRKCN rather than S731AS735A mutant (Figure 6O,P). Noteworthily, S731AS735A mutant still maintained the capacity to interact with mTOR, ruling out the possibility that its functional defect may be due to defective mTOR binding (Figure 6Q). Taken together, activation loop phosphorylation rather than kinase activity was indispensable for PRKCN exploiting mTOR‐IRF4 axis to foster uncontrolled cell growth.

### 2.7 PRKCN Silencing Evokes Interferon (IFN) Signaling Pathway Partly Though IRF4 Downregulation and Confers Increased Susceptibility to IFN Treatment

Activation of type I IFN signaling has been recently linked to augmented tumoricidal activity and increased susceptibility to standard therapeutic agents in MM [33, 34]. We noticed that signaling pathways relevant to IFNα and IFNγ were enriched upon PRKCN KD by performing GSEA (Figure 5A; Figure S22A). Moreover, PRKCN silencing triggered transcriptional upregulation of IFNs and a series of IFN‐stimulated genes (ISGs) coupled with elevated protein abundance of p‐Stat1 in both MM.1S and U266 cells (Figure S22B,C). The transcriptional upregulation of IFNs and ISGs, together with increased p‐Stat1 protein expression was further validated in excised MM.1S xenografts with Dox‐induced PRKCN knockdown (Figure S22D,E).

IRF4 acted as a transcriptional repressor of ISGs in B‐cell lymphoma [35, 36], prompting us to explore whether PRKCN may suppress IFN signaling by exploiting IRF4 in MM cells. Since the impact of IRF4 on IFN signaling remains largely unknown in MM cells, we first examined this by utilizing shRNA technology to deplete IRF4 in HMCLs. Upon successful IRF4 knockdown, both MM.1S and U266 cells displayed upregulation of multiple ISGs (Figure S22F,G), supporting that IRF4 indeed negatively regulated transcription of ISGs in MM cells. Meanwhile, IRF4 knockdown closely mimicked the effect of PRKCN knockdown to upregulate IFNα and IFNγ in U266 cells (Figure S22G), hinting that IRF4 may be largely responsible for PRKCN‐mediated downregulation of IFNs and ISG in U266 cells. In contrast, IRF4 knockdown did not influence IFNβ expression in MM.1S cells (Figure S22F), implying that IRF4 suppressed ISGs expression independently of affecting autocrine IFNβ expression. By analyzing the publicly‐available IRF4 ChIP‐Seq dataset, we confirmed binding of IRF4 to the promoter region of representative ISGs (IFI44, IFIT3, IFITM1, IFITM2 and MX1) in MM.1S cells (Figure S22H), corroborating direct transcriptional repression of ISGs. In alignment with this, overexpression of wild‐type IRF4 instead of DNA binding‐defective IRF4 can efficiently repress transcription of ISGs (Figure S22I). To further ascertain whether IRF4 may be involved in ISGs modulation elicited by PRKCN, we performed rescue experiment and found that transcriptional upregulation of several ISGs following Dox‐mediated PRKCN knockdown was considerably blunted by IRF4 overexpression in MM.1S cells (Figure S22J). Then we examined whether PRKCN may affect the response of myeloma cells to IFNβ. IFNβ treatment alone impaired cell viability in both time‐dependent and dose‐dependent manner, and more importantly, an addictive loss of cell viability was noted when IFNβ treatment was combined with Dox‐induced PRKCN disruption, while PRKCN overexpression protected against the growth inhibition induced by IFNβ treatment (Figure S23A–C), suggesting that inactivation of PRKCN not only triggered IFN signaling pathway to suppress cell growth but also conferred increased sensitivity to IFNβ treatment in MM cells.

### 2.8 Pharmacological Suppression of PRKCN by CRT0066101 Impairs Cell Growth, Increases Drug Sensitivity, Overcomes Drug Resistance and Elicits Strong Efficacy in CDX and PDX Models

**FIGURE 7:**
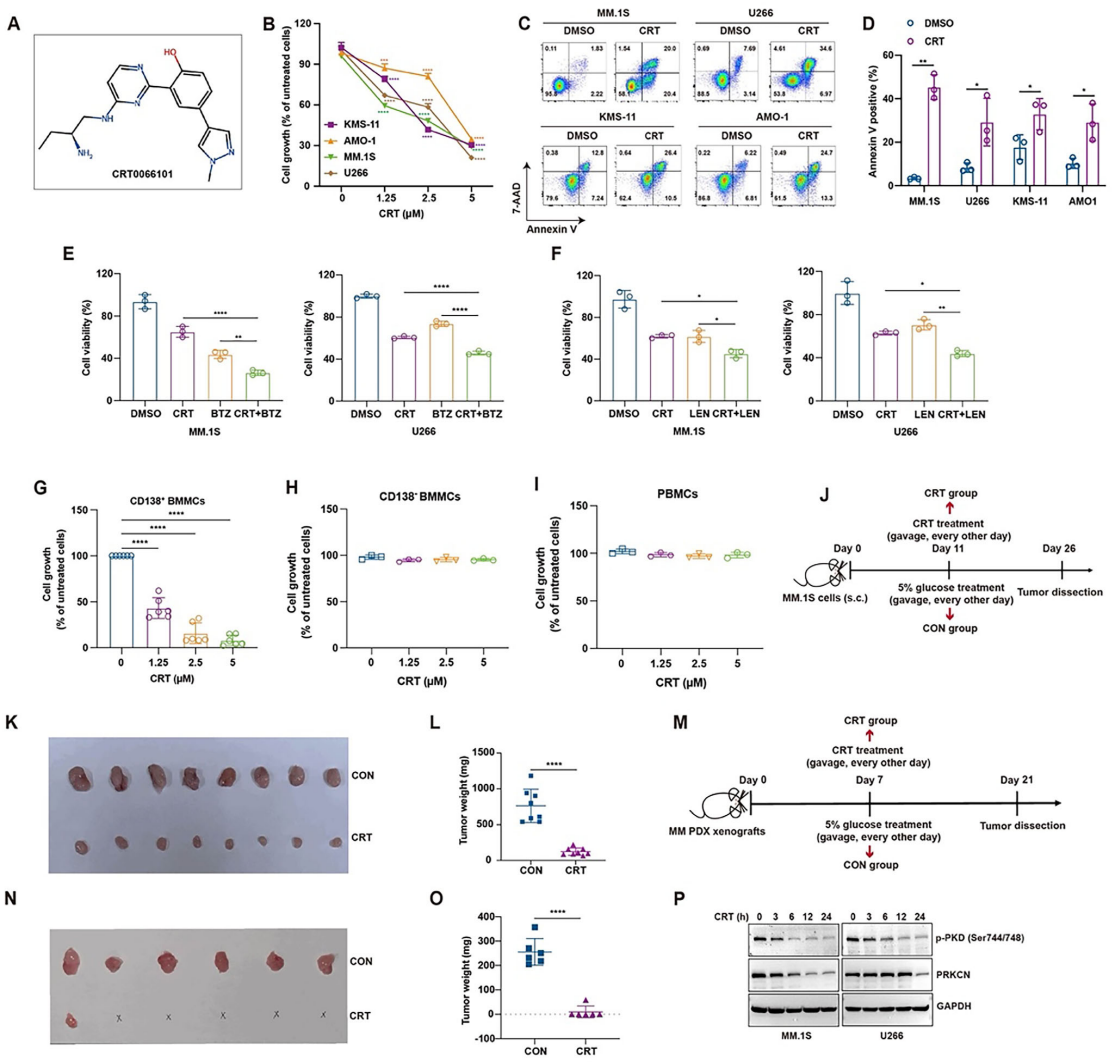
Pharmacological suppression of PRKCN by CRT0066101 displays potent anti‐MM efficacy in vitro and in vivo. (A) The chemical structural formula of CRT0066101 (CRT) (created with ChemDraw). (B) KMS‐11, AMO‐1, MM.1S and U266 cells were incubated with CRT for 48 h, and cell viability was assessed by the CCK‐8 assay. One‐way ANOVA with Dunnett's post hoc test was performed, *n* = 3. (C,D) KMS‐11, AMO‐1, MM.1S (2.5 µm) and U266 (5 µm) cells were exposed to CRT at the indicated concentrations for 48 h, and apoptosis was determined by measuring Annexin V‐positive cells using a flow cytometer. Data are presented as mean ± SD. Unpaired two‐tailed *t* test or an unpaired two‐tailed Welch' *t* test was performed, *n* = 3. (E) Cell viability of MM.1S and U266 cells treated with CRT (0.625 or 1.25 µm) in the presence or absence of BTZ (0.625 nm) was assessed by the CCK‐8 assay. (F) Cell viability of MM.1S and U266 cells incubated with CRT (0.625 or 1.25 µm) with or without LEN (5 µm) was evaluated by CCK‐8 assay. Data are presented as mean ± SD (E, F). One‐way ANOVA with Tukey's post hoc test was performed, *n* = 3. (G‐H) BMMCs isolated from 6 myeloma patients were treated with CRT for 24 h, and the survival rate of CD138^+^ myeloma cells (G) and CD138^−^BMMCs (H) was quantified by absolute counting. (I) PBMCs from 3 healthy donors were treated with CRT for 24 h, with survival rate detected by CCK8 assay. Data are presented as mean ± SD (G–I). One‐way ANOVA with Dunnett's post hoc test was performed, *n* = 3 or 6. (J) MM.1S cells were injected subcutaneously into NCG mice, starting from 11 days post‐inoculation, mice received CRT (80 mg/kg weight) or vehicle (5% glucose in PBS) via gavage every other day. (K,L) Tumors were harvested and photographed (K), and tumor weight was qualified (L) at the endpoint. Data are presented as mean ± SD (L). Unpaired two‐tailed Welch' *t* test was performed, *n* = 8. (M) NCG mice were subcutaneously inoculated with primary myeloma cells, and the cells from the resultant tumors were subsequently implanted in a new batch of NCG mice on day 0. Subsequently, mice were administered with CRT (80 mg/kg weight) or vehicle (5% glucose in PBS) via gavage every other day starting from seventh day. (N,O) Tumors were harvested and photographed (N), and tumor weight was qualified (O) at the endpoint. Data are presented as mean ± SD (O). Unpaired two‐tailed *t* test was performed, *n* = 6. (P) Immunoblot analysis of PRKCN and p‐PKD (Ser744/748) protein levels in MM.1S and U266 cells treated with CRT for indicated time periods.

CRT0066101 (CRT), an orally bioavailable pan‐PKD inhibitor capable of suppressing catalytic activity of PRKCN, significantly abrogated tumor growth in multiple xenograft models without causing apparent systemic toxicity, thereby representing an ideal candidate for clinical development [37]. We evaluated the efficacy of CRT (Figure 7A) on MM. CRT treatment significantly impaired cell viability (Figure 7B), and promoted apoptosis (Figure 7C,D). Combinatory application of CRT with BTZ or LEN exhibited a stronger growth‐inhibitory effect than any monotherapy (Figure 7E,F). Noticeably, CRT treatment significantly impeded the growth of BTZ‐ or LEN‐resistant MM.1S cells (Figure S24A). In addition, co‐treatment with CRT and BTZ conferred augmented growth‐inhibitory effect on BTZ‐resistant cells (Figure S24B). Importantly, CRT treatment efficiently eliminated primary MM cells while largely sparing the patient‐derived CD138^−^ BMMCs or normal PBMCs (Figure 7G–I). As anticipated, CRT treatment apparently slowed down tumor growth rate, and reduced tumor size and weight at the time of sacrifice in the subcutaneous MM.1S CDX (cell line‐derived xenograft) model (Figure 7J–L). Moreover, CRT treatment remarkably refrained tumor dissemination and reduced the overall tumor burden in the systemic MM.1S CDX model (Figure S25A–C). Strikingly, in the PDX (patient‐derived xenograft) model established by subcutaneous inoculation of primary myeloma cells from a refractory patient, five out of six mice achieved complete tumor regression following treatment (Figure 7M–O).

### 2.9 CRT Disturbs PRKCN Expression and Activation Loop Phosphorylation, Represses mTOR‐IRF4 Axis and Activates IFN Signaling Both In Vitro and In Vivo

**FIGURE 8:**
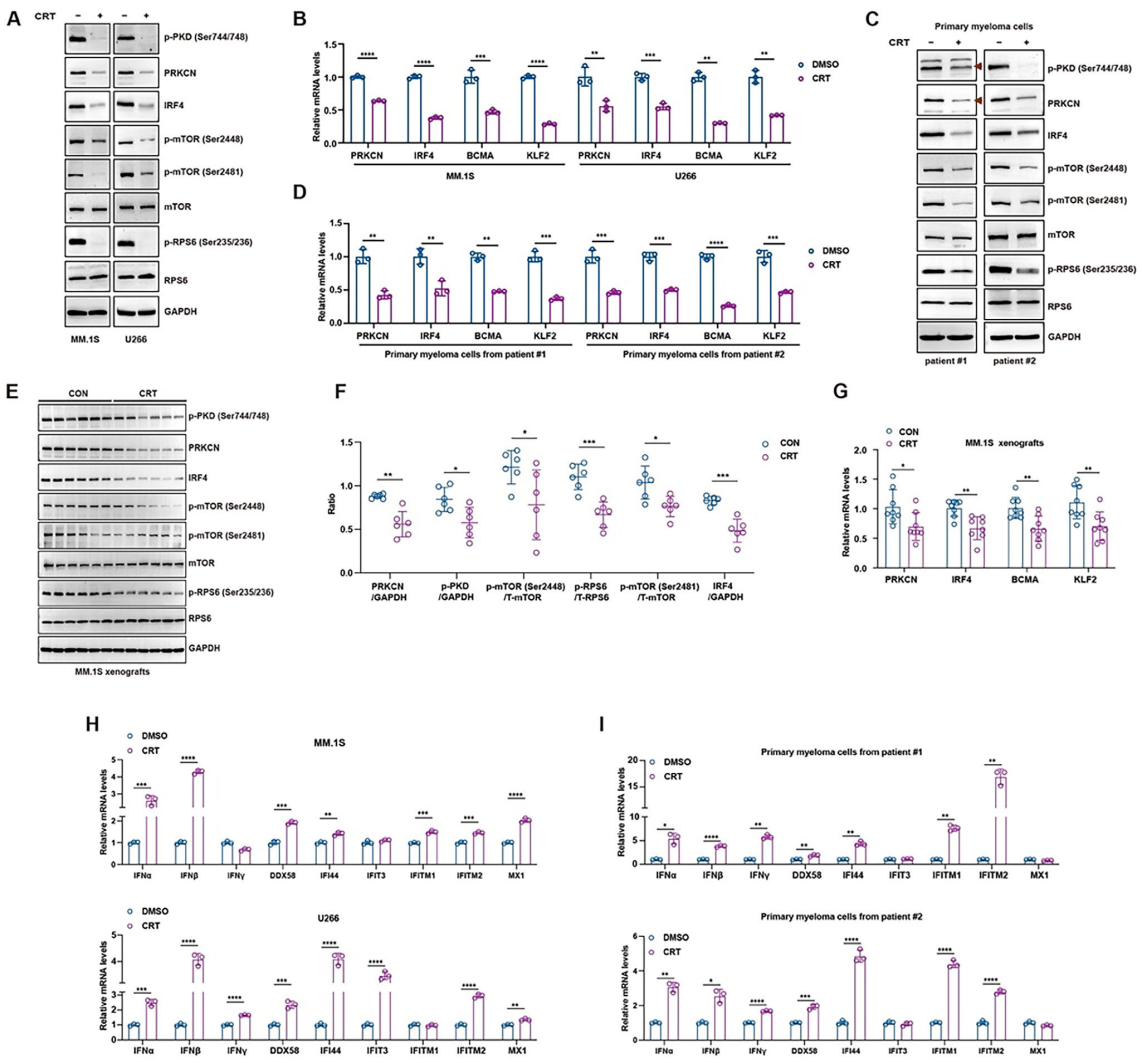
CRT treatment impairs the PRKCN‐mTOR‐IRF4 axis but activates IFN signaling in vitro and in vivo. (A) Immunoblot analysis of PRKCN, p‐PKD, IRF4, mTOR, p‐mTOR, RPS6 and p‐RPS6 in MM.1S or U266 cells subjected to CRT treatment for 24 h. (B) RT‐qPCR analysis of PRKCN, IRF4, BCMA and KLF2 in MM.1S or U266 cells treated with CRT for 24 h. (C,D) Two primary samples were treated with CRT for 12 h, and analyzed for protein levels of PRKCN, p‐PKD, IRF4, mTOR, p‐mTOR, RPS6 and p‐RPS6 by immunoblot analysis, and for mRNA levels of PRKCN, IRF4, BCMA and KLF2 by RT‐qPCR analysis. For RT‐qPCR, data are presented as mean ± SD. Unpaired two‐tailed *t* test was performed, *n* = 3. (E) Immunoblot analysis of PRKCN, p‐PKD, IRF4, mTOR, p‐mTOR, RPS6 and p‐RPS6 in the xenografts from each group. (F) The graphs summarize PRKCN, p‐PKD, IRF4, p‐mTOR, and p‐RPS6 band intensities normalized to GAPDH quantified. Data are presented as mean ± SD. Unpaired two‐tailed *t* test was performed, *n* = 6. (G) RT‐qPCR analysis of PRKCN, IRF4, BCMA and KLF2 mRNA expression in the xenografts from each group. Data are presented as mean ± SD. Unpaired two‐tailed *t* test was performed, *n* = 8. (H,I) The mRNA levels of IFNα, IFNβ, IFNγ, DDX58, IFI44, IFIT3, IFITM1, IFITM2 and MX1 in MM.1S, U266 cells and primary MM samples treated with CRT by RT‐qPCR analysis. Data are presented as mean ± SD. Unpaired two‐tailed *t* test was performed, *n* = 3.

Since PRKCN relied on activation loop phosphorylation to promote cell growth, we examined whether CRT affected activation loop phosphorylation. Indeed, CRT obviously hindered phosphorylation of Ser731 and Ser735 in the activation loop as detected by anti‐p‐PKD (Ser744/748) antibody in both cell lines (Figures 7P and 8A). In U266 cells, the decline in p‐PKD (Ser744/748) levels occurred apparently earlier than the reduction in total PRKCN protein following CRT treatment, corroborating that CRT may specifically disturb the phosphorylation of PRKCN's activation loop in this cell line. In contrast, in MM.1S cells, p‐PKD and total PRKCN protein levels appeared to decrease simultaneously upon CRT treatment, suggesting that the decrease in p‐PKD signal could potentially result from the downregulation of total PRKCN protein, rather than reflecting specific inhibition of its phosphorylation status (Figures 7P and 8A,B). Therefore, CRT may hinder activation loop phosphorylation of PRKCN in different ways depending on the cellular context. CRT blocked mTORC1/C2 signaling pathways, as judged by decreased p‐mTOR (Ser2448), p‐RPS6 (Ser235/236) and p‐mTOR (Ser2481) in both cell lines, which was accompanied by downregulation of IRF4 and its downstream BCMA and KLF2 (Figure 8A,B; Figure S25D). This impact was also recapitulated in CRT‐treated primary myeloma cells (Figure 8C,D) and MM.1S xenograft model (Figure 8E–G). Collectively, PRKCN acts as an essential driver of mTOR‐IRF4 axis in MM and can be therapeutically targeted with CRT. In addition, CRT treatment activated IFN signaling in HMCLs, primary MM cells as well as MM.1S xenograft, as manifested by transcriptional upregulation of IFNs and ISGs (Figure 8H,I; Figure S25E).

## 3 Discussion

PRKCN has been implicated in the initiation and progression of solid tumors by augmenting cellular proliferation, migration, invasion and chemoresistance through activating a series of signaling pathways such as PI3K/p38‐AKT, MEK/ERK and NF‐κB pathways [38, 39], while its specific role in MM remains grossly unexplored. We presently demonstrate that PRKCN is a crucial tumor‐promoting gene driven by transcriptionally activated SEs in MM cells. Consistently, PRKCN is abundantly expressed in MM cell lines and primary samples. Since PRKCN is increased at stages of MGUS and SMM, it may be an early event in the pathogenesis of MM. Meanwhile, high PRKCN expression is correlated with adverse prognosis of both newly diagnosed and refractory/relapsed MM, hinting its involvement in treatment resistance and disease progression as a high‐risk gene.

PRKCN transcription is elicited by activated NF‐κB signaling pathway either existing intrinsically in MM cells or exogenously triggered by TNF‐α derived from BMSCs in the bone marrow milieu. Moreover, PRKCN is directly transactivated by NF‐κB signaling via promoter and SE engagement and also mediates the latter's growth‐promoting role, thus emerging as a novel and essential transcriptional target of NF‐κB signaling. Interestingly, contrary to the stimulatory impact of PRKCN on NF‐κB signaling pathway in other cancers [40, 41], PRKCN was found to negatively modulate NF‐κB signaling in HMCLs (Figure S19), which may represent a unique negative feedback mechanism.

Gain‐of‐function and loss‐of‐function studies unequivocally demonstrated that PRKCN fosters MM cell growth, clonogenicity and tumorigenecity but restraining apoptosis. Moreover, PRKCN thwarts the susceptibility of MM cells to standard therapeutic agents, including BTZ and LEN, and BTZ‐ or LEN‐resistant cells remain sensitive to PRKCN depletion. Therefore, PRKCN disruption has the potential to provide significant clinical benefit either alone or in combination with BTZ or LEN. Since PRKCN is not genetically altered in most MM, we envisage that MM cells generally display non‐oncogenic addiction to PRKCN activity.

Although both mTORC1/C2 signaling pathways and IRF4‐driven transcriptional network play crucial roles in the pathogenesis of MM, facilitating cell growth, survival and drug resistance [42, 43], their relationship has not been clearly defined. The CRISPR/Cas9 library screening by others complemented with our experimental study utilizing shRNA technique evidently support that IRF4 transcription is subject to regulation by mTORC1/C2 signaling pathways [23]. By employing different intervention strategies including genetic manipulation and treatment with specific small‐molecule activators or inhibitors, we demonstrate that PRKCN boosts MM cell growth through activation of the mTORC1/C2‐IRF4 axis. Nevertheless, how PRKCN‐elicited mTORC1/C2 activation tunes IRF4 transcription remains obscure. The oncogene c‐MYC may serve as a potential downstream effector of mTORC1/C2 to induce IRF4 transcription considering that c‐MYC has been reported to drive IRF4 transcription by promoter binding by others [22], and also validated by us in MM.1S cells (Figure S26A–C). This hypothesis is further supported by the observation that specific knockdown of PRKCN, mTOR, Raptor, RPS6 or Rictor in MM.1S cells consistently resulted in diminished c‐MYC expression at post‐transcriptional levels but decreased IRF‐4 expression from transcriptional levels (Figures S12B, S18B–I, and S26D–G). Noticeably, further investigation is required to ascertain whether c‐MYC really mediates the stimulatory effect of PRKCN on IRF4 transcription and MM cell growth. Intriguingly, downregulation of c‐MYC protein levels triggered by PRKCN silencing was partly rescued by IRF4 overexpression (Figure S26H,I), which might be interpreted by the presence of a positive feedback loop between c‐MYC and IRF4 via reciprocally transcriptional regulation [22]. However, PRKCN unlikely harnesses c‐MYC to influence IRF4 in U266 cells, which are known to express L‐MYC rather than c‐MYC [44], and therefore additional study is needed to identify the specific mediators involved. Essentially, IRF4 reciprocally transactivates PRKCN by SE binding, nominating PRKCN as a novel target of IRF4 in MM cells. However, we cannot exclude the possibility that IRF4 may indirectly enhance PRKCN transcription by modulating some intermediate transcription factors, which merits further investigation. Cumulatively, PRKCN coordinates with IRF4 to drive MM cell growth by forming an autoregulatory circuit.

In deciphering the machinery whereby PRKCN co‐opts mTORC1/C2 signaling pathways, we disclose that PRKCN directly and extensively associates with mTOR. In vitro kinase assay suggests that PRKCN may act as a typical kinase to activate mTORC1/C2 signaling pathways through directly phosphorylating mTOR, while further study via the introduction of three functionally defective PRKCN mutants yields some unexpected findings. Specially, the kinase‐inactive K605N and D720A mutants with intact activation loop phosphorylation, which is indeed catalyzed by the PKC isozymes via the so‐called trans‐phosphorylation rather than by PRKCN itself [32], maintain the capacity to augment mTOR‐IRF4 signaling and MM cell growth, while S731AS735A mutant which abolishes activation loop phosphorylation to render PRKCN catalytically inactive appears to be functionally deficient. Based on the aforementioned evidence, it is plausible to postulate that PRKCN predominantly relies on activation loop phosphorylation instead of its acknowledged kinase activity to orchestrate mTOR‐IRF4 signaling in MM cells, defining an unprecedented action mode of PRKCN. Admittedly, one major limitation of this study lies in that the precise mechanism responsible for activation of mTORC1/C2 signaling by PRKCN remains to be decoded. At this stage, it is tempting to speculate that PRKCN with intact activation loop phosphorylation may simultaneously recruit some still unknown partners to facilitate activation of mTORC1/C2 signaling upon interacting with mTOR, which undoubtedly merits deep investigation.

Exploration of potential therapeutic strategies capable of activating type I IFN signaling has grabbed considerable attention for MM treatment in recent years, since it is closely associated with augmented tumoricidal activity and enhanced drug sensitivity. It has been recently reported that single treatment with modakafusp alfa, an anti‐CD38‐targeted attenuated IFNα, displays a response rate of 43% in relapsed or refractory MM patients with manageable toxicity, which was connected with induced myeloma cell death and enhanced immune activation [45]. The immunomodulatory drug LEN was found to activate the MDA5‐mediated double‐stranded RNA (dsRNA)–sensing pathway, leading to IFN‐mediated apoptosis in MM cells, while ADAR1 loss increased LEN sensitivity by triggering dsRNA‐sensing pathways and enhanced IFN responses [46]. In addition, DOTIL inactivation enhanced the anti‐MM efficacy of LEN by activating IFN signaling through suppression of IKZF1/3 and STING in MM cells [34]. We presently disclose that perturbation of PRKCN can activate type I IFN signaling to impair MM cell growth, increase the sensitivity to IFN treatment, and boost the anti‐MM efficacy of LEN, presenting a novel strategy of effectively activating IFN signaling. Noteworthily, as the downstream effector of PRKCN, IRF4 seems to mainly mediate PRKCN‐induced inhibition of IFN signaling by transcriptional repression of ISGs via direct promoter binding, yet it is not responsible for regulation of IFNβ in MM.1S cells. Based on the preliminary observation such as increased protein expression of γH2A.X as a marker of DNA damage (Figure S27A,B), and transcriptional upregulation of DDX58 (RIG‐1) (Figure S22B,D) following perturbation of PRKCN, together with some hints from GSEA on the RNA‐seq data (Figure S27C), we speculate that PRKCN inactivation may induce IFNβ expression through activating a “viral mimicry” state and evoking DNA sensor pathway and/or dsRNA‐sensing pathway, which warrants further investigation.

We uncover that CRT, an orally bioavailable pan‐PKD kinase inhibitor, remarkably impairs the growth of drug‐naive and drug‐resistant cell lines as well as primary MM cells, potentiates growth‐inhibitory effect of BTZ or LEN and displays robust anti‐MM efficacy in two different CDX models and a PDX model, corroborating that CRT may be a promising therapeutic agent for MM. Mechanistically, CRT impairs PRKCN expression and activation loop phosphorylation, leading to dampened mTOR‐IRF4 signaling axis. The latter is expected to further diminish PRKCN expression and activation loop phosphorylation presumably by disturbing IRF4‐PRKCN feedback loop. In addition, analogous to PRKCN knockdown, CRT treatment efficiently activates type I IFN signaling which potentially enhances tumoricidal activity both in vitro and in vivo. Noteworthily, CRT does not launch apparent cytotoxicity toward normal PBMCs and BMMCs, suggesting a potentially favorable therapeutic index. Our findings lay a certain theoretical and experimental foundation for future clinical trials on CRT alone or in combination with standard therapies in refractory MM.

**FIGURE 9:**
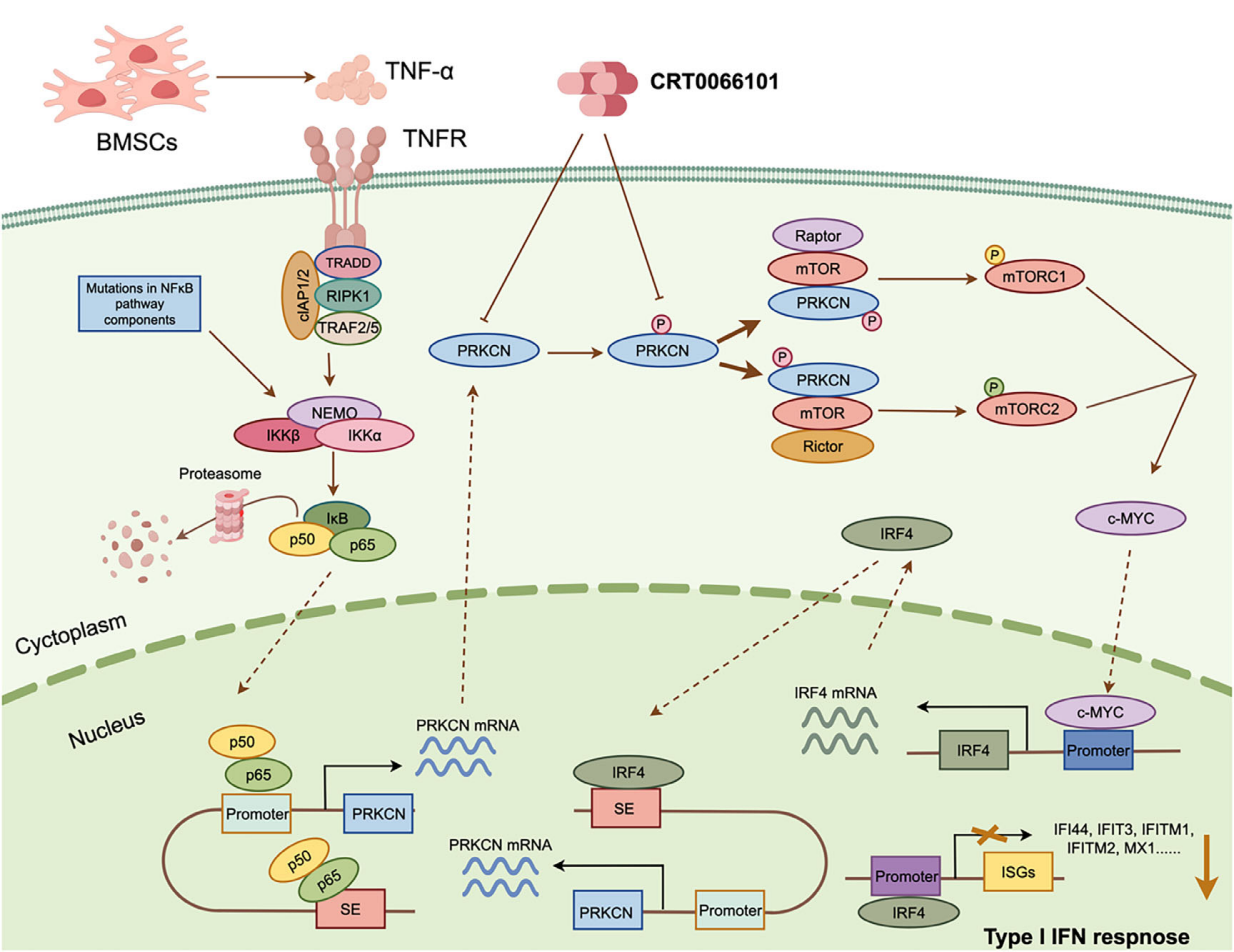
Schematic diagram of this study. The hypothetical molecular mechanism model underlying aberrant expression or activation of PRKCN as well as PRKCN‐triggered mTOR‐IRF4 axis in MM. PRKCN is highly expressed as a SE‐associated gene and is transactivated via SE and promoter binding by overactivated NF‐κB signaling existing intrinsically or exogenously provoked by TNF‐α secreted by BMSCs. Upon trans‐phosphorylation at its activation loop, PRKCN may directly bind mTOR to trigger activation of both mTORC1 and mTORC2 signaling pathways in the kinase activity‐independent but activation loop phosphorylation‐dependent fashion. The activated mTORC1 and mTORC2 signaling pathways subsequently increase the expression of the essential mediators such as c‐MYC at the post‐transcriptionally levels, which in turn transcriptionally induces expression of IRF4 as a master regulator of MM cell growth and survival. In addition, PRKCN may negatively modulate type I IFN signaling partly through IRF4‐mediated transcriptional repression and impair the susceptibility to IFN treatment. Meanwhile, IRF4 reciprocally drives PRKCN transcription via SE binding, forming an auto‐regulatory circuit to further amplify the impact. The orally bioavailable inhibitor CRTCRT0066101 diminishes PRKCN expression, impedes the activation loop phosphorylation of PRKCN and disrupts the mTOR‐IRF4 axis, thereby exerting robust anti‐MM efficacy. (Schematic diagram was created with Figdraw.).

Overall, our study highlights the biological significance and clinical relevance of SE‐driven PRKCN, which especially bridges hyperactivated NF‐κB signaling with aberrant mTOR‐IRF4 signaling (Figure 9), and establishes a strong rationale for developing activation loop phosphorylation‐directed inhibitor of PRKCN for MM therapy.

## 4 Experimental Methods

### 4.1 Chemicals and Reagents

JQ1, doxycycline, B022, bortezomib, lenalidomide, MHY‐1485 and puromycin were purchased from MCE. Human TNF‐α and IL‐6 were from novoprotein. MLN120B and CRT0066101 were from APExBIO and selleckchem, respectively.

### 4.2 Cell Culture

RPMI‐8226 (RRID: CVCL_0014), MM.1S (RRID: CVCL_8792), U266 (RRID: CVCL_0566), HS‐5 (RRID: CVCL_3720) and HEK293T (RRID: CVCL_0063) were originally acquired from ATCC, KMS‐11 (RRID: CVCL_2989) cell line from JCBR, and AMO‐1 (RRID: CVCL_1806) cell line from DSMZ. RPMI‐8226, MM.1S, U266,

KMS‐11 and AMO‐1 cell lines were cultured in RPMI 1640 medium containing 10% FBS, HS‐5 and HEK293T cell lines were maintained in DMEM containing 10% FBS. For generation of bortezomib‐resistant or lenalidomide‐resistant cell lines, MM.1S cells were continuously exposed to escalating doses of individual drugs. All cell lines were tested negative for mycoplasma contamination before use in experiments.

### 4.3 Co‐Immunoprecipitation (Co‐IP) Assay

Cells were lysed in the IP lysis buffer containing protease inhibitor and phosphatase inhibitor at 4°C. For endogenous IP, protein samples were coupled with the protein A/G magnetic beads; while for exogenous IP, protein samples were incubated with anti‐Flag M2 affinity gel on a rotator at 4°C overnight. The protein beads or Flag gel were washed with the IP wash buffer and boiled in SDS loading buffer for SDS‐PAGE and immunoblot analysis.

### 4.4 GST Pull‐Down Assay

Recombinant GST‐tagged PRKCN protein, recombinant Flag‐tagged mTOR protein and anti‐GST Magnetic beads were incubated in 1xIP wash buffer in the presence of protease inhibitor and phosphatase inhibitor on a rotator at 4°C overnight. Then the beads were washed 5 times with IP wash buffer and boiled in laemmli buffer for 10 min for SDS‐PAGE and immunoblot analysis.

### 4.5 Gene Expression and ChIP‐Seq Analyses Using Publicly Available Datasets

The transcription profiles of various cell lines were retrieved from the DepMap portal and the Human Protein Atlas portal. The following datasets deposited in the Gene Expression Ominibus (GEO) with the accession numbers, GSE5900, GSE2658, GSE24080, GSE9782, GSE145938, GSE2912, GSE13591, GSE26760, GSE19784, GSE44931 and GSE4581 were selected for gene expression analysis. The NF‐κB index indicative of NF‐κB signaling activity was based on a previous study [47], and calculated as the average Log_2_ values of 10 genes after normalization. Analyses of overall survival (OS), progression‐free survival (PFS) or event‐free survival (EFS) in association with PRKCN expression for MM patients in GSE24080 (including TT2 and TT3 therapies) and GSE9782 datasets were performed using the Kaplan Meier plotter (www.kmplot.com) with log‐rank tests for significance. The visualization of the ChIP‐seq results output from Sequence Read Archive (SRA) database (SRX4401064, SRX2404142, SRX4947721, SRX378805, SRX1460847, SRX150605, SRX017882, SRX017894, SRX017872, SRX017906, SRX327760, and SRX129116) was performed using IGV software, ChIP‐Atlas portal, UCSC Gene Brower or Cistrome Data Brower.

### 4.6 Human Primary MM cells, BMMCs, PBMCs, BMSCs and Conditioned Medium

Human bone marrow or peripheral blood samples were derived from MM patients or healthy donors after collecting informed consent to participate in research protocols approved by the Faculty Hospital Ethics Committee at the First Affiliated Hospital of Soochow University (Suzhou, China). All samples were from Hematological Biobank, Jiangsu Biobank of Clinical Resources. Human bone marrow mononuclear cells (BMMCs) and peripheral blood mononuclear cells (PBMCs) were isolated using lymphoprep by density gradient centrifugation. Primary MM cells were purified from bone marrow aspirates of patients with CD138 microbeads [48]. Residual CD138^−^ BMMCs were further cultured in DMEM containing 15% FBS for 4–6 weeks to generate long‐term primary bone marrow stromal cells (BMSCs). For preparation of the conditioned medium (CM), primary BMSCs or HS‐5 cells were refed with fresh RPMI1640 medium containing 10% FBS and cultured for 48 or 72 h, after which the culture supernatants were harvested and filtered.

### 4.7 Quantitative Real‐Time‐Polymerase Chain Reaction (RT‐qPCR)

Total RNA was isolated using Trizol reagent according to the manufacturer's instructions. cDNA was synthesized using oligo dT primers and Superscript II reverse transcriptase. Real‐time qPCR was performed with SYBY Green supper Mixture Reagents on a BD LightCycler 96 Real‐Time PCR System. The transcript levels were analyzed by 2^−ΔΔCt^ method with β‐actin mRNA as the internal control. The primer sequences used were detailed in Table S1.

### 4.8 Immunoblot Analysis

Cells were lysed using RIPA buffer, and soluble proteins were resolved by SDS‐PAGE gels and transferred to PVDF membranes. The membranes were probed with primary antibodies followed by incubation with a horseradish peroxidase–conjugated secondary antibodies. Antibody binding was detected using an enhanced chemiluminescence reagent, and imaging was performed by the Image VCD system. The primary antibodies involved anti‐PRKCN, anti‐p‐PKD/PKC𝜇 (Ser731Ser735 for PRKCN), anti‐IRF4, anti‐NF‐κB p65, anti‐p‐NF‐κB p65 (Ser536), anti‐mTOR, anti‐p‐mTOR (Ser2448), anti‐p‐mTOR (Ser2481), anti‐Raptor, anti‐Rictor, anti‐RPS6, anti‐p‐RPS6 (Ser235/236), anti‐DYKDDDDK Tag (Flag), anti‐HA Tag, anti‐GST Tag, anti‐IκBα, anti‐c‐MYC, NF‐κB2 p100/p52, AKT, p‐AKT (Ser473), Stat1, p‐Stat1 (Tyr701), p‐H2A.X (Ser139), anti‐GAPDH. The detailed information of the antibodies was shown in Table S2.

### 4.9 Plasmid Construction

To generate the lentiviral expression constructs for PRKCN, IRF4, IκBαM and firefly luciferase, the corresponding coding domain sequences (CDS) were PCR‐amplified from the cDNA derived from MM.1S cells or our deposited plasmids and then ligated into the lentiviral backbones Venus‐Flag or PCDH.SFFV.IRES.Puro. The generation of expression constructs encoding PRKCN shRNA#1‐resistant mutants with or without one of three different site‐specific mutations including D720A, K605N and S731AS735A within the catalytic domain of PRKCN was carried out by using Q5 Site‐Directed Mutagenesis Kit. The lentiviral construct encoding IRF4 mutant lacking DNA‐binding domain was generated by overlapping PCR using wild‐type IRF4 as the template. In addition, the CDS of RELA (p65), mTOR, PRKCN as well as the truncated mutants of mTOR and PRKCN were PCR‐amplified and inserted into pCDNA3.1, pCDNA3.1‐Flag or pcDNA3.1‐HA backbone to yield the corresponding expression constructs for achieving transient expression.

For achieving constitutive knockdown of PRKCN, IRF4, mTOR, Raptor, RPS6 or Rictor, the annealed shRNA oligonucleotides targeting individual gene of interest were ligated into pLKO‐TRC‐GFP backbone. A scramble shRNA was used as a control. For achieving inducible knockdown of PRKCN, the same annealed shRNA oligonucleotides targeting PRKCN (#1) or scramble shRNA as the control were inserted into Tet‐pLKO‐TRC‐EGFP or Tet‐pLKO‐TRC‐NGFR vector, respectively.

For CRIPSR/Cas9‐mediated knockout of PRKCN, RELA or IRF4, the single guide RNA sequences (sgRNAs) specifically targeting the individual genes were cloned into the modified lentiCRISPR v2 vector with Puro cassette replaced with EGFP. For CRISPRi/dCas9‐KRAB‐mediated suppression of PRKCN super‐enhancer, sgRNAs targeting the proximal promoter or upstream distant enhancer locus of PRKCN were subcloned into Lenti‐(BB)‐EF1α‐KRAB‐dCas9‐P2A‐GFP vector. The sgRNA sequences including non‐targeting control were included for both gene knockout and super‐enhancer suppression.

For generating the reporter promoter construct pGL3‐PRKCNpro‐luc‐WT, the proximal PRKCN promoter region ranging from −1032 bp to +114 bp relative to the transcription start site was amplified from the genomic DNA of MM.1S cells and subcloned into the pGL3‐basic luciferase reporter plasmid. For generation of the enhancer reporter construct PRKCNenh‐luc‐WT, the 800 bp DNA fragment corresponding to the upstream distant enhancer of PRKCN with the potential p65‐ and IRF4‐binding sites were synthesized and inserted into pGL4.23 reporter vector containing the SV40 minimal promoter. Overlapping PCR technique was utilized for mutating the potential p65‐binding sites to generate pGL3‐PRKCNpro‐luc‐p65‐MT and pGL4.23‐PRKCNenh‐luc‐p65‐MT constructs.

All the constructs generated were verified by Sanger sequencing analysis through GENEWIZ from Azenta Life Sciences (Suzhou, China). The primer sequences used for plasmid construction were listed in Table S3.

### 4.10 Lentiviral Packaging and Transduction

The individual lentiviral expression vector was co‐transfected with the packaging plasmids psPAX2 and pMD2G into HEK293T cells [49]. 48 h after transfection, infectious lentiviruses were harvested and filtered through 0.45 µm PVDF filters. For infection, MM cells were incubated in the media containing the viral supernatants for 6–8 h, and the infection was repeated on the next day. Following transduction, GFP‐positive cells or NGFR‐positive cells, following antibody staining were sorted by flow sorter and expanded for subsequent functional assays. For lentivirus‐mediated inducible shRNA knockdown of PRKCN, 1 µg/ml doxycycline was used to treat the cells for inducing expression of shRNA. For the “rescue” experiment, cells were infected with the individual viruses and selected by flow cytometry sorting based on GFP or NGFR expression sequentially. To generate MM.1S cells, MM.1S‐Tet‐shNT cells and MM.1S‐Tet‐shPRKCN cells stably expressing firefly luciferase (FFL), the corresponding cell lines were transduced with lentiviruses encoding FFL and then selected with puromycin.

### 4.11 Cell Growth Assay

Cell proliferation and viability were assessed by a cell counting Kit‐8 (CCK‐8) following the manufacturer's instruction. The absorbance was measured at 450 nm using a microplate reader.

### 4.12 Soft Agar Clonogenicity Assay

MM cells were seeded in RPMI 1640 medium supplemented with 0.33% low‐melt agarose and 10% FBS, with the medium replenished twice weekly. After 2–3 weeks of culture, colonies were stained with 0.01% crystal violet solution and visualized using a light microscope.

### 4.13 Apoptosis and Cell Cycle Analysis

For apoptosis detection, cells were treated as indicated and then stained with Annexin/7‐AAD. For cell cycle analysis, cells were labelled with 5‐bromo‐2'‐deoxyuridine (BrdU) and then subject to fixation and permeabilization. Cells were then treated with DNase and stained with DAPI. The stained cells were analyzed by ACEA Novo cytometer equipped with ACEA NovoExpress Software. All data analysis was performed using Flow Jo v.10.

### 4.14 Primary Cell Viability Analysis

The normal PBMCs or patient BMMCs were treated with CRT0066101 or DMSO as the vehicle for 24 h, and then BMMCs were stained with anti‐CD138 antibody to distinguish CD138^+^ primary MM cells from CD138^−^ BMMCs. Afterward, 123count eBeads and 7‐AAD were added, and the absolute number of viable cells were quantified by flow cytometry analysis according to the manufacture's protocol.

### 4.15 Flow Cytometry Analysis

BMMCs were stained with anti‐CD138 antibody to identify malignant plasma cells. For surface detection of BCMA, MM cells or primary myeloma cells were stained with anti‐BCMA antibody. The antibodies used were listed in Table S4.

### 4.16 Luciferase Reporter Assays

pCDNA‐p65, Venus‐IRF4, Venus‐IRF4‐∆DBD or the corresponding empty vectors were co‐transfected with either pGL3‐PRKCNpro‐luc‐WT/p65‐MT or pGL4.23‐PRKCNenh‐luc‐WT/p65‐MT plasmids into HEK293T cells together with the pRL‐CMV plasmid for normalization of transfection efficacy using Lipofectamine 2000. 24 h post‐transfection, luciferase activity was determined with Dual‐Luciferase Reporter Assay System.

### 4.17 Chromatin Immunoprecipitation (ChIP) Assays

ChIP assays were carried out using a Magna A/G Chromatin Immunoprecipitation Kit (Merck Millipore) according to the manufacturer's instructions as described previously [50]. Briefly, an equal amount of anti‐p65 antibody, anti‐IRF4 antibody, anti‐c‐MYC antibody or normal rabbit IgG as the control was separately used to precipitate the cross‐linked DNA‐protein complexes derived from 2 × 10^7^ MM.1S or U266 cells, respectively. Following reversal of cross‐linking, the DNA immunoprecipitated by the indicated Ab was tested by quantitative PCR (qPCR). The corresponding primer sequences were listed in Table S5.

### 4.18 Human MM Xenograft Models

All animal experiments were approved by the Soochow University Animal Care and Use Committee. Male NCG mice at age of 6–8 weeks were obtained from Gem Pharmatech Co., Ltd (Nanjing, China). For evaluating the effect of PRKCN overexpression on the tumorigenicity, 2 × 10^6^ RPMI‐8226‐EV or RPMI‐8226‐PRKCN cells were inoculated into the flanks of mice subcutaneously. For evaluating the effect of PRKCN depletion in the MM.1S xenograft mouse model by virtue of a Tet‐On inducible knockdown system, 5 × 10^6^ MM.1S cells harboring Tet‐shNT or Tet‐shPRKCN with or without co‐expression of FFL were injected subcutaneously. 5 days or 7 days after implantation, mice were administrated either doxycycline (l mg/ml in 5% sucrose) or vehicle (5% sucrose) via drinking water for the duration of the study. For the subsequent IRF4 “rescue” in the same model, 5 × 10^6^ MM.1S cells stably expressing EV+Tet‐shPRKCN or IRF4+Tet‐shPRKCN were inoculated subcutaneously. One week after implantation, mice were randomly divided and administrated either doxycycline (l mg/ml in 5% sucrose) or vehicle (5% sucrose) via drinking water for the duration of the study.

For assessing the effect of CRT0066101 in the MM.1S xenograft models, NCG mice were subcutaneously injected with 5×10^6 ^MM.1S cells or alternatively intravenously inoculated with 5 × 10^6^ MM.1S‐FFL cells. At the indicated time points, tumor‐bearing mice were randomly divided to orally receive CRT at the dose of 80 mg/kg or the vehicle (5% glucose in PBS) every other day, respectively.

For evaluating the effect of CRT0066101 in a patient‐derived xenograft (PDX) model, fresh myeloma cells were isolated from pleural effusion in a refractory MM patient were mixed with Matrigel and subcutaneously inoculated into NCG mice. Cells isolated from the resultant tumors were subsequently inoculated into a new batch of mice, which were randomly divided to receive CRT or vehicle every other day, respectively.

Mice were weighed and observed daily for any changes in behavior or condition. For monitoring the subcutaneous tumor growth, a caliper was used to measure the sizes of tumors every day and tumor sizes were calculated according to the formula: 0.5 × length × width^2^_._ At the indicated time points, tumor‐bearing mice were euthanized, and tumors were dissected and processed for subsequent application. For monitoring the disseminated tumor growth, multiple rounds of bioluminescence imaging were applied to record the changes in luciferase signal intensity.

### 4.19 RNA‐Seq Analysis

MM.1S cells transduced with PRKCN shRNA or scramble shRNA for 72 h (*n* = 3) were collected in Trizol reagent and sent to oebiotech company (Shanghai, China) for RNA sequencing. Data analysis was performed with DESeq2 R package, and transcripts with *q*‐value <0.05 and |log_2_ FC| >0.58 were considered significant.

### 4.20 Gene Set Enrichment Analysis (GSEA)

GSEA was performed using the GSEA software (https://www.broadinstitute.org/gsea/) from Broad Institute or R package. The predefined hallmark gene sets were downloaded from the Molecular Signatures Database (MSigDB). Transcriptome signature of genes positively regulated by IRF4 in MM cells was retrieved from a previous study [22]. Analysis was run with the defaulted 1000 permutations, and pathways or hallmarks with p <0.05 and false discovery rate (FDR) <0.25 were considered significantly enriched.

### 4.21 In Vitro Kinase Assay

Indicated amounts of recombinant GST‐tagged PRKCN and Flag‐tagged mTOR proteins were incubated in Kinase buffer for 60 min at 37°C in the presence of 20 µm ATP. Then SDS loading buffer was added to stop the reaction. Phosphorylation of mTOR at Ser2448 and Ser2481 was analyzed by immunoblot analysis with the specific antibodies.

### 4.22 Indirect Immunofluorescence Staining

HEK293T cells that were transiently co‐transfected with pCDNA3.1‐HA‐PRKCN and pCDNA3.1‐Flag‐mTOR plasmids were fixed with 4% paraformaldehyde (PFA), permeabilized in 1% Triton‐X100, and sealed in PBS with 2% BSA at room temperature (RT). The samples were incubated with anti‐Flag antibody at 4°C overnight, and then with Alexa Fluor 488‐conjugated secondary antibody at RT for 1 h. Following three washes, the samples were incubated with anti‐HA tag antibody at 4°C overnight and then incubated with Alexa Fluor 568‐conjugated secondary antibody at RT for 1 h. Cells were washed and counterstained with DAPI, and then imaged on a Nikon A1 Ti2 confocal microscope with 63x oil immersion objective.

### 4.23 Statistical Analyses

Graphpad Prism software v10.0 was used for statistical analyses and graph generation. For normally distributed data, statistically significant differences between groups were determined using two‐tailed unpaired Student's t test, one‐way or two‐way ANOVA followed by Dunnett's, Tukey's, Skidak's, Holm‐Skidak's, Bonferroni's post hoc tests as indicated (^*^ *p*  <0.05, ^**^ *p*  <0.01, ^***^ *p*  <0.001, ^****^ *p*  <0.0001). For non‐normal data, the Mann–Whitney test or the Kruskal–Wallis test followed by the Dunn multiple comparison test was used for comparisons. Co‐expression correlation analysis was conducted by Spearman's correlation analysis.

## Author Contributions

K.T., D.J., P.K., and X.B. designed and performed experiments and analyzed data. L.Z. and Y.Z. performed experiments and analyzed data. T.Z., W.Q., and W.Q. contributed to the data analysis and interpretation of the results. J.C., D.W., C.F., and Y.X. jointly supervised the project, designed experiments, analyzed data and wrote the manuscript.

## Funding

This work was supported by the grants from the National Key R&D Program of China (2022YFC2502700 (Y.X.), National Natural Science Foundation of China (81770216 (J.C.), 82370224 (J.C.), 82020108003 (D.W.), 82200204 (D.J.)), Natural Science Foundation of Jiangsu Province ((BK20220248 (D.J.)), Shenzhen Science and Technology Program (JCYJ20250604141706008 (P.K.)), Priority Academic Program Development of Jiangsu Higher Education Institutions (PAPD).

## Conflicts of Interest

The authors declare no conflicts of interest.

## Supporting information




**Supporting File**: advs74248‐sup‐0001‐SuppMat.docx.

## Data Availability

The raw RNA‐seq data generated in this study have been deposited in the Gene Expression Omnibus (GEO) database under accession code GSE305283. All other data supporting the findings of this study are available in the article and its Supplementary Information file.
