# Supplementary material for: Targeting PRKCN, an Essential Driver Orchestrating mTOR‐IRF4 Axis Independently of Kinase Activity, in Multiple Myeloma
Source: Adv Sci (Weinh). 2026 Feb 8;13(21):e18975. doi: 10.1002/advs.202518975 (PMC13073330; doi:10.1002/advs.202518975)
Supplement: Supplementary file 1 — Supporting File: advs74248‐sup‐0001‐SuppMat.docx. [file ADVS-13-e18975-s001.docx]

**Supplementary Tables**

**Supplementary Table S1. List of primers used for RT-qPCR**

| **Primer** | **FW** | **RV** |
| --- | --- | --- |
| β-actin | CCATCATGAAGTGTGACGTGG | GTCCGCCTAGAAGCATTTGCG |
| PRKCN | CTTCCAGATTCGTCCACATACTC | ATCCACAGCCTTCACATTTCAG |
| IRF4 | ATCACAGCTCACGTAGAACCT | TGGATTGCTGATGTGTTCTGG |
| BMCA | GTAATGCAAGTGTGACCAATTCAG | GCTCTTGATGCAGTCTTCAC |
| KLF2 | CAAGAGTTCGCATCTGAAGG | TACCAGTCACAGTTTGGGAG |
| mTOR | TCGCTGAAGTCACACAGACC | CTTTGGCATATGCTCGGCAC |
| Raptor | AATCAACCCAAATCACCCTC | ATCCAGTTCAGTTCACCCAG |
| Rictor | GGAAGCCTGTTGATGGTGAT | GGCAGCCTGTTTTATGGTGT |
| RPS6 | TCAGTGGTGGGAACGACAAACAAG | TACTCAGTAGCAGGCGGACACG |
| IFNα | CTCTCCTTCCTCCTGTCTGATGG | TGGAGCCTTCTGGAACTGGTTG |
| IFNβ | GGACGCCGCATTGACCATCTATG | ACAATAGTCTCATTCCAGCCAGTGC |
| IFNγ | CTGACTTGAATGTCCAACGCAAAGC | CGACCTCGAAACAGCATCTGACTC |
| Stat1 | AATACACCTACGAACATGACCCT | TTTCACCAACAGTCTCAACTTCAC |
| ISG15 | CATCTTTGCCAGTACAGGAG | CCGATCTTCTGGGTGATCTG |
| DDX58 | GAAGACCCTGGACCCTACCT | TCCATTGGGCCCTTGTTGTT |
| IFI44 | ACTTGGATTTGCTCTTTCTGAC | CCCTTAGATTCCCTATTTGCTC |
| IFIT3 | GGGCAGACTCTCAGATGCTC | CATCGCAATTGCCAGTCCAG |
| IFITM1 | GATCAACATCCACAGCGAGAC | CCTGTCCCTAGACTTCACGG |
| IFITM2 | CTGACCATGTGGTCTGGTCC | TCGCCAACCATCTTCCTGTC |
| MX1 | GTTACCAGGACTACGAGATTGAG | GATGAGTGTCTTGATCTTATACCC |
| c-MYC | AATGAAAAGGCCCCCAAGGTAGTTATC | GTCGTTTCCGCAACAAGTCCTCTTC |

**Supplementary Table S2. List of antibodies for immunoblotting or immunofluorescence**

| **Target** | **Supplier** | **Dilution** |
| --- | --- | --- |
| GAPDH | Sungene Biotech (#KM9002T) | 1:5000 |
| PKD3/PKCν | CST (#5655) | 1:1000 |
| p-PKD/PKCμ (Ser744/748) | CST (#2054) | 1:1000 |
| NF-κB p65 | CST (#8242) | 1:1000 |
| p-NF-κB p65 (Ser536) | CST (#3033) | 1:1000 |
| IRF4 | CST (#4964) | 1:1000 |
| IκBα | Proteintech (#10268-1-AP) | 1:2000 |
| mTOR | Proteintech (#66888-1-lg) | 1:1000 |
| p-mTOR (Ser2448) | CST (#5536) | 1:1000 |
| p-mTOR (Ser2481) | CST (#2974) | 1:1000 |
| Raptor | Proteintech (#20984-1-AP) | 1:2000 |
| Rictor | CST (#2114) | 1:1000 |
| RPS6 | Proteintech (#66886-1-lg) | 1:2000 |
| p-RPS6 (Ser235/236) | CST (#4858) | 1:1000 |
| HA-Tag | CST (#3724) | 1:1000 |
| DYKDDDDK Tag | CST (#14793) | 1:1000 |
| GST Tag | CST (#2622) | 1:1000 |
| NF-κB2 p100/p52 | CST (#37359) | 1:1000 |
| AKT | CST (#9272) | 1:1000 |
| p-AKT (Ser473) | CST (#4060) | 1:1000 |
| Stat1 | CST (#14997) | 1:1000 |
| p-Stat1 (Tyr701) | CST (#9167) | 1:1000 |
| p-H2A.X (Ser139) | CST (#9718) | 1:1000 |
| c-MYC | CST (#9402) | 1:1000 |
| Anti-Flag^®^ M2 antibody | Sigma (#F1804) | 1:1000 |
| Normal Rabbit IgG | CST (#2729) | 1:1000 |
| Goat anti-Rabbit IgG (H+L) HRP | Bioworld (#BS13278) | 1:10000 |
| Goat anti-Mouse IgG (H+L) HRP | Bioworld (#BS12478) | 1:10000 |
| Anti-mouse lgG (H+L) (Alexa Fluor^@^ 488 conjugate) | CST (#4408) | 1:500 |
| Anti-rabbit lgG (H+L) (Alexa Fluor^@^ 647 conjugate) | CST (#4410) | 1:500 |

**Supplementary Table S3.** **List of primers used for plasmid construction**

| **Plasmid** | **Primer FV/RV** |
| --- | --- |
| Lenti-BB-SFFV-KRAB-dCas9-CON-P2A-EGFP | sgControl-For:  CACCGAACCTACGGGCTACGATACG  sgControl -Rev:  AAACCGTATCGTAGCCCGTAGGTTC |
| Lenti-BB-SFFV-KRAB-dCas9-SE2KD-P2A-EGFP | PRKCN-SE2-KD-For:  CACCGGTTCTGAAAGTGCTGCCCTC  PRKCN-SE2-KD-Rev:  AAACGAGGGCAGCACTTTCAGAACC |
| Lenti-BB-SFFV-KRAB-dCas9-ProKD-P2A-EGFP | PRKCN-Pro-KD-For:  CACCGTGCACTCCACTTTGGTGGTA  PRKCN-Pro-1KD-Rev:  AAACTACCACCAAAGTGGAGTGCAC |
| Lenti-BB-SFFV-KRAB-dCas9-SE1KD-P2A-EGFP | PRKCN-SE1-KD-For:  CACCGCTGTGCAAGACCTGAATCTG  PRKCN-SE1-KD-Rev:  AAACCAGATTCAGGTCTTGCACAGC |
| pcDNA3.1-p65 | p65-For:  CGGGATCCATGGACGAACTGTTCCCCCTCATC  p65-Rev:  GCTCTAGATTAGGAGCTGATCTGACTCAGCAG |
| pGL3-PRKCNpro-luc-WT | PRKCNp-1032upKpnI:  GGGGTACCCAGCAGCTCCAGGCTGCAGTG  PRKCNp-114dwNheI:  CTAGCTAGCCAGCGTCTGAGTACCGGACGAG |
| pGL3-PRKCNpro-luc-p65-MT | RKCNp-1032upKpnI:  GGGGTACCCAGCAGCTCCAGGCTGCAGTG  p65-PKD-mut1Rev:  GTTCCGGTGACTGCATTAACCGGAACGGTGCTCGGTGTTTCCTCCCCGTC  p65-PKD-mut2For:  TTCCGGTTAATGCAGTCACCGGAACGTCGGGCTTG  PRKCNp-114dwNheI:  CTAGCTAGCCAGCGTCTGAGTACCGGACGAG |
| pGL4.23-PRKCNenh-luc-WT | PCNENH-KpnI-1For:  GGGGTACCCTCAAAATGGGAACATAGAGT  PCNENH-NheI-2Rev:  CTAGCTAGCAGCCAGGAGCCAGATGCTGGG |
| pGL4.23-PRKCNenh-luc-p65-MT | PCNENH-KpnI-1For:  GGGGTACCCTCAAAATGGGAACATAGAGT  PCNENH-mut-1Rev:  TTAACCGGAATAGGTATGCCTCTTGCCTCCT  PCNENH-mut-2For:  GGCATACCTATTCCGGTTAATGGAGTTCAACTGAAACTTG  PCNENH-NheI-2Rev:  CTAGCTAGCAGCCAGGAGCCAGATGCTGGG |
| pLKO.1-shscramble-GFP | shscramble-For:  CCGGCCTAAGGTTAAGTCGCCCTCGCTCGAGCGAGGGCGACTTAACCTTAGGTTTTTG  shscramble-Rev:  AATTCAAAAACCTAAGGTTAAGTCGCCCTCGCTCGAGCGAGGGCGACTTAACCTTAGG |
| pLKO.1-shPRKCN-1-GFP | shPRKCN-1-For:  CCGGCACGACCAACAGATACTATAACTCGAGTTATAGTATCTGTTGGTCGTGTTTTTG  shPRKCN-1-Rev:  AATTCAAAAACACGACCAACAGATACTATAACTCGAGTTATAGTATCTGTTGGTCGTG |
| pLKO.1-shPRKCN-2-GFP | shPRKCN-2-For: CCGGCCGGGCGGGAAACTGAATAATAAGACTCGAGTCTTATTATTCAGTTTCCCGCCCGGTTTTTG  shPRKCN-2-Rev:  AATTCAAAAAGTGGATTCTTTGGCATGTATGCTCGAGCATACATGCCAAAGAATCCAC |
| pLKO.1-shPRKCN-RMut-GFP | PRKCN-sh1RMut-For:  GCAAAAGGATTCCTTCTTGGAGTGGTC  PRKCN-sh1RMut-Rev:  TCGGCTCTTGGTGGACATGTGACTCTTC |
| pLKO.1-shIRF4-1-GFP | shIRF4-1-For: CCGGGTGCCATTTCTCAGGGAAGTACTCGAGTACTTCCCTGAGAAATGGCACTTTTTG  shIRF4-1-Rev:  AATTCAAAAAGTGCCATTTCTCAGGGAAGTACTCGAGTACTTCCCTGAGAAATGGCAC |
| pLKO.1-shIRF4-2-GFP | shIRF4-2-For:  CCGGGCCATTCCTCTATTCAAGAATCTCGAGATTCTTGAATAGAGGAATGGCTTTTTG  shIRF4-2-Rev:  AATTCAAAAAGCCATTCCTCTATTCAAGAATCTCGAGATTCTTGAATAGAGGAATGGC |
| pLKO.1-shmTOR-1-GFP | shmTOR-1-For:  CCGGGCTGTGCTACACTACAAACATCTCGAGATGTTTGTAGTGTAGCACAGCTTTTTG  shmTOR-1-Rev:  AATTCAAAAAGCTGTGCTACACTACAAACATCTCGAGATGTTTGTAGTGTAGCACAGC |
| pLKO.1-shmTOR-2-GFP | shmTOR-2-For:  CCGGCAGGCCTATGGTCGAGATTTACTCGAGTAAATCTCGACCATAGGCCTGTTTTTG  shmTOR-2-Rev:  AATTCAAAAACAGGCCTATGGTCGAGATTTACTCGAGTAAATCTCGACCATAGGCCTG |
| pLKO.1-shRaptor-1-GFP- | shRaptor-1-For:  CCGGGGCTAGTCTGTTTCGAAATTTCTCGAGAAATTTCGAAACAGACTAGCCTTTTTG  shRaptor-1-Rev:  AATTCAAAAAGGCTAGTCTGTTTCGAAATTTCTCGAGAAATTTCGAAACAGACTAGCC |
| pLKO.1-shRaptor-2-GFP- | shRaptor-2-For:  CCGGAGGGCCCTGCTACTCGCTTTTCTCGAGAAAAGCGAGTAGCAGGGCCCTTTTTTG  shRaptor-2-Rev:  AATTCAAAAAAGGGCCCTGCTACTCGCTTTTCTCGAGAAAAGCGAGTAGCAGGGCCCT |
| pLKO.1-shRPS6-1-GFP | shRPS6-1-For:  GCTCTAGAATGAAGCTGAACATCTCCTTC  shRPS6-1-Rev:  GGTTTACCGGTTTATTTCTGACTGGATTCAGAC |
| pLKO.1-shRPS6-2-GFP | shRPS6-2-For:  CCGGGCCCTTAAATAAAGAAGGTAACTCGAGTTACCTTCTTTATTTAAGGGCTTTTTG  shRPS6-2-Rev:  AATTCAAAAAGCCCTTAAATAAAGAAGGTAACTCGAGTTACCTTCTTTATTTAAGGGC |
| pLKO.1-shRictor-1-GFP | shRictor-1-For:  CCGGACTTGTGAAGAATCGTATCTTCTCGAGAAGATACGATTCTTCACAAGTTTTTTG  shRictor-1-Rev:  AATTCAAAAAACTTGTGAAGAATCGTATCTTCTCGAGAAGATACGATTCTTCACAAGT |
| pLKO.1-shRictor-2-GFP | shRictor-2-For:  CCGGCGTCGGAGTAACCAAAGATTACTCGAGTAATCTTTGGTTACTCCGACGTTTTTG  shRictor-2-Rev:  AATTCAAAAACGTCGGAGTAACCAAAGATTACTCGAGTAATCTTTGGTTACTCCGACG |
| pLKO.1-shc-MYC-GFP | shc-MYC-For:  CCGGCCTGAGACAGATCAGCAACAACTCGAGTTGTTGCTGATCTGTCTCAGGTTTTTG  shc-Myc-Rev:  AATTCAAAAACCTGAGACAGATCAGCAACAACTCGAGTTGTTGCTGATCTGTCTCAGG |
| Tet-pLKO.1-U6-shPRKCN-1-NGFR | shPRKCN-1-For:  CCGGCACGACCAACAGATACTATAACTCGAGTTATAGTATCTGTTGGTCGTGTTTTTG  shPRKCN-1-Rev:  AATTCAAAAACACGACCAACAGATACTATAACTCGAGTTATAGTATCTGTTGGTCGTG |
| PU6-sgp65-SFFV-GFP | p65-CR1F:  CACCGGGAAGATCTCATCCCCACCG  p65-CR1R:  AAACCGGTGGGGATGAGATCTTCCC |
| PU6-sgPRKCN-SFFV-GFP | PRKCNCR7-For: CACCGTGAAGGAGACCTAGTGGAAG  PRKCNCR7-Rev: AAACCTTCCACTAGGTCTCCTTCAC |
| PU6-sgIRF4-SFFV-GFP | IRF4-CR1-For: CACCGCGTTCTCCCACACCAGCCCG  IRF4-CR1-Rev: AAACCGGGCTGGTGTGGGAGAACGC |
| Venus-IκBαM | IκBαM-For:  GCTCTAGAATGTTCCAGGCGGCCGAGCGT  IκBαM-Rev:  CCCACCGGTTCATAACGTCAGACGCTGGCC |
| Venus-IRF4 | IRF4-For: GCTCTAGAATGAACCTGGAGGGCGGCGGCCGA  IRF4-Rev:  TTTACCGGTTCATTCTTGAATAGAGGAATGGCGG |
| Venus-IRF4-△DBD | IRF4-△DBD-For: GCTCTAGAATGACCTTGGAGGACCCGCAGATG  IRF4-Rev: TTTACCGGTTCATTCTTGAATAGAGGAATGGCGG |
| Venus-PRKCN | PRKCN-For:  GCTCTAGAATGTCTGCAAATAATTCCCCTCCAT  PRKCN-Rev：  TTTACCGGTTTAAGGATCTTCTTCCATATCATCT |
| Venus-PRKCN K605N | PRKCN-K605N-For:  TGGCTATTAACGTAATTGATAAGATGAG  PRKCN-K605N-Rev:  CATCCCTCCCAGTCTTTC |
| Venus-PRKCN D720A | PRKCN-D720A-For:  GAAGCTGTGTGCCTTTGGATTTG  PRKCN-D720A-Rev:  ACCTGAGGAAATGGC |
| Venus-PRKCN S731AS735A | PRKCN-S731AS735A-For:  GAGAGCTGTGGTAGGAACTCCAGCA  PRKCN-S731AS735A-Rev:  CTGAATGCCTTTTCACCAATGATGCGTG |
| pcDNA3.1-PRKCN-Flag | PRKCN-For:  ATAAGAATGCGGCCGCCATGTCTGCAAATAATTCCCCTCCAT  PRKCN-Rev:  GCTCTAGATTAAGGATCTTCTTCCATATCATCT |
| pcDNA3.1-PRKCN-HA | PRKCN-HA-For:  ATAAGAATGCGGCCGCCATGTCTGCAAATAATTCCCCTCCAT  PRKCN-HA-Rev:  GCTCTAGATTAAGGATCTTCTTCCATATCATCT |
| pcDNA3.1-PRKCN-C1-HA | PRKDCN-HA-For: ATAAGAATGCGGCCGCCATGTCTGCAAATAATTCCCCTCCAT  PRKCN-C1-Rev: GCTCTAGATTAGCTGCTCTTCCTCTTTGTGTG |
| pcDNA3.1-PRKCN-PH-HA | PRKDCN-PH-HA-For: ATAAGAATGCGGCCGCCATGGGAGAGGTTACTTTCAATGGA  PRKDCN-PH-HA-Rev: GCTCTAGATTAGATATCCACATTCTCCTGAAT |
| pcDNA3.1-PRKCN-Kinase-HA | PRKCN-Kinase-HA-For: ATAAGAATGCGGCCGCCATGGCAAGTGTTTGCACTTCTCCA  PRKCN-Rev:  GCTCTAGATTAAGGATCTTCTTCCATATCATCT |
| pcDNA3.1-mTOR-Flag | mTOR-For:  AGCTTTGTTTAAACATGCTTGGAACCGGACCTGCC  mTOR-Rev:  ATAAGAATGCGGCCGCCACCAGAAAGGGCACCAGCCAAT |
| pcDNA3.1-mTOR-HA | mTOR-HA-For:  ATAAGAATGCGGCCGCCATGCTTGGAACCGGACCTGCC  mTOR-HA-Rev:  ATAAGAATGCGGCCGCTTACCAGAAAGGGCACCAGCC |
| pcDNA3.1-mTOR-(1-1482aa)-HA | mTOR-HA-For: ATAAGAATGCGGCCGCCATGCTTGGAACCGGACCTGCC  mTOR-1482aa-Rev: ATAAGAATGCGGCCGCTTAGCGCATGCGGCCCAGCATCAG |
| pcDNA3.1-mTOR-(1271-2008aa)-HA | mTOR-1271aa-For: ATAAGAATGCGGCCGCCATGGCTGCCAGGAGGGTCTCCAAAG  mTOR-2008aa-Rev:  ATAAGAATGCGGCCGCTTACTGCTGGACCAGGGTGTTGCTG |
| pcDNA3.1-mTOR-(1750-2549aa)-HA | mTOR-1750aa-For:  ATAAGAATGCGGCCGCCATGTGCTTCCTGAAACTTGGAGAG  mTOR-Rev: ATAAGAATGCGGCCGCTTACCAGAAAGGGCACCAGCC |
| pcDNA3.1-mTOR-(1241-2549aa)-HA | mTOR-2141aa-For:  ATAAGAATGCGGCCGCCATGCCAGGAACATATGACCCCAAC  mTOR-Rev: ATAAGAATGCGGCCGCTTACCAGAAAGGGCACCAGCC |
| FFL-luc-puro | FFL-For:  TGCTCTAGAATGGAAGACGCCAAAAACATA  FFL-Rev:  ATAAGAATGCGGCCGCTTACACGGCGATCTTTCCGCC |

**Supplementary Table S4. List of antibodies for flow cytometry staining**

| **Target** | **Clone** | **Supplier** | **Dilution** |
| --- | --- | --- | --- |
| Annexin V | / | Biolegend (#640919) | 1:100 |
| 7-AAD | / | BD (#559925) | 1:100 |
| BrdU | Bu20a | Biolegend (#339808) | 1:50 |
| CD271 (NGFR) | ME20.4 | Biolegend (#345107) | 1:100 |
| CD269 (BCMA) | 19F2 | Biolegend (#357505) | 1:100 |
| CD38 | HIT2 | BD (#562444) | 1:100 |
| CD138 | MI1S | BD (#562097) | 1:100 |

**Supplementary Table S5. List of primers used for ChIP-qPCR**

| **Primer** | **FW** | **RV** |
| --- | --- | --- |
| PRKCNpro-p65 | CTGGACTGCACTCCACTTTGG | TCCAGAGCAGAGAGCTGCTTC |
| PRKCNpro-IRF4 | TTTCGGTGTCTGTTCAGCGCT | CTCGTCCGGTACTCAGACGCT |
| PRKCNenh-p65 | GGGAGGCTCTGAGCTTCAGAG | CTCATCTCTCTTCCCTGGGAC |
| PRKCNenh-IRF4 | AGCCAGATGCTGGGTGTAAGC | GGACTTCCTCAAATGTCCTTC |
| IRF4pro-c-MYC-site1 | ACCTCGCACTCTCAGTTTCAC | TCTCTGGTATCAGCCTCACAC |
| IRF4pro-c-MYC-site2 | GGTGCTTCTGAGCAGGAAGGC | AGAAATCACGAGGTTGCCGAG |

**Supplementary Table S6. Clinical paraments of MM patients**

| Patient | Age | Sex | Type of lg | Clinical stage | | | Medical history |
| --- | --- | --- | --- | --- | --- | --- | --- |
|  |  |  |  | D&S | ISS | R-ISS |  |
| P1 | 60 | F | lgG-λ | III | II | III | Newly diagnosed |
| P2 | 58 | M | lgG-λ | III | III | III | Newly diagnosed |
| P3 | 52 | M | lgG-κ | III | I | I | Newly diagnosed |
| P4 | 39 | M | lgG-κ | III | II | II | Newly diagnosed |
| P5 | 74 | M | lgA-λ | III | II | II | Newly diagnosed |
| P6 | 63 | M | lgA-λ | III | II | II | Newly diagnosed |
| P7 | 67 | F | lgA-λ | III | II | II | VTD^a)^, auto-HSCT^b)^, DPD^c)^ |
| P8 | 61 | F | lgD-λ | III | I | I | Newly diagnosed |
| P9 | 70 | F | lgG-κ | III | II | II | VRD^d)^, auto-HSCT |
| P10 | 61 | M | lgM-κ | III | II | II | Newly diagnosed |
| P11 | 64 | M | lgA-λ | III | II | II | Newly diagnosed |
| P12 | 48 | F | lgD-λ | III | II | II | Newly diagnosed |

^a)^VTD; bortezomib, thalidomide, dexamethasone; ^b)^HSCT; hematopoietic stem cell transplantation; ^c)^DPD; daratumumab, pomalidomide, dexamethasone.^d)^VRD; bortezomib, lenalidomide, dexamethasone.

**Supplementary Figures**

**
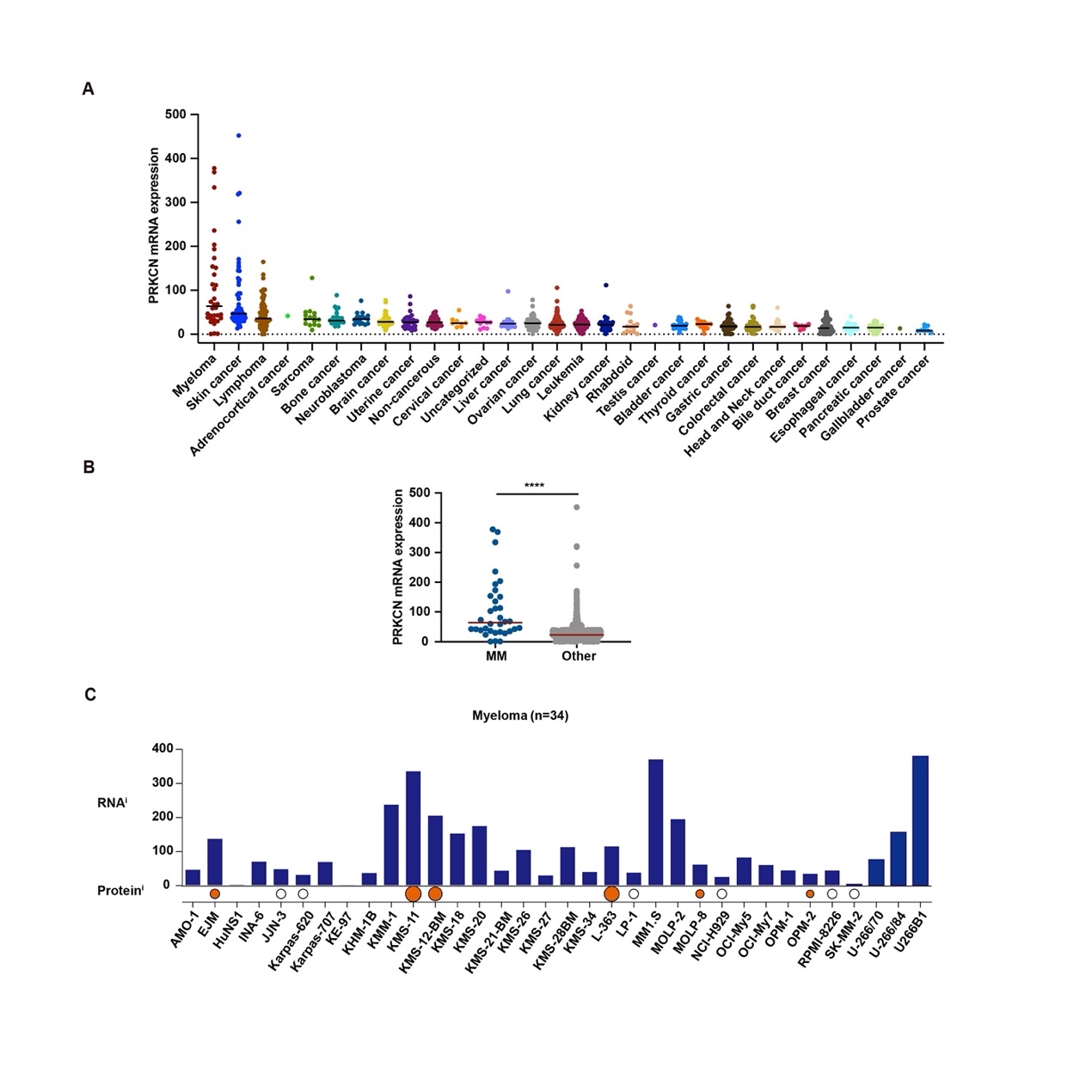
**

**Figure S1. Expression pattern of PRKCN in human tumor cell lines by analyzing Human Protein Atlas database.**

(A) Dotplots illustrating PRKCN mRNA expression in different types of cancer cell lines (unit: nTPM). (B) Dotplots comparing expression of PRKCN transcripts in human MM (n = 34) versus other tumor cell lines (n = 1172) (unit: nTPM). The middle line represents the median value. Mann-Whitney test was performed. (C) Bar plot depicting PRKCN mRNA and protein expression in 34 MM cell lines (unit: nTPM or nRPX). The MS protein expression data is represented by a circle for each cell line group with size corresponding to expression level and color intensity representing the percentage of cell lines in the group having MS data.


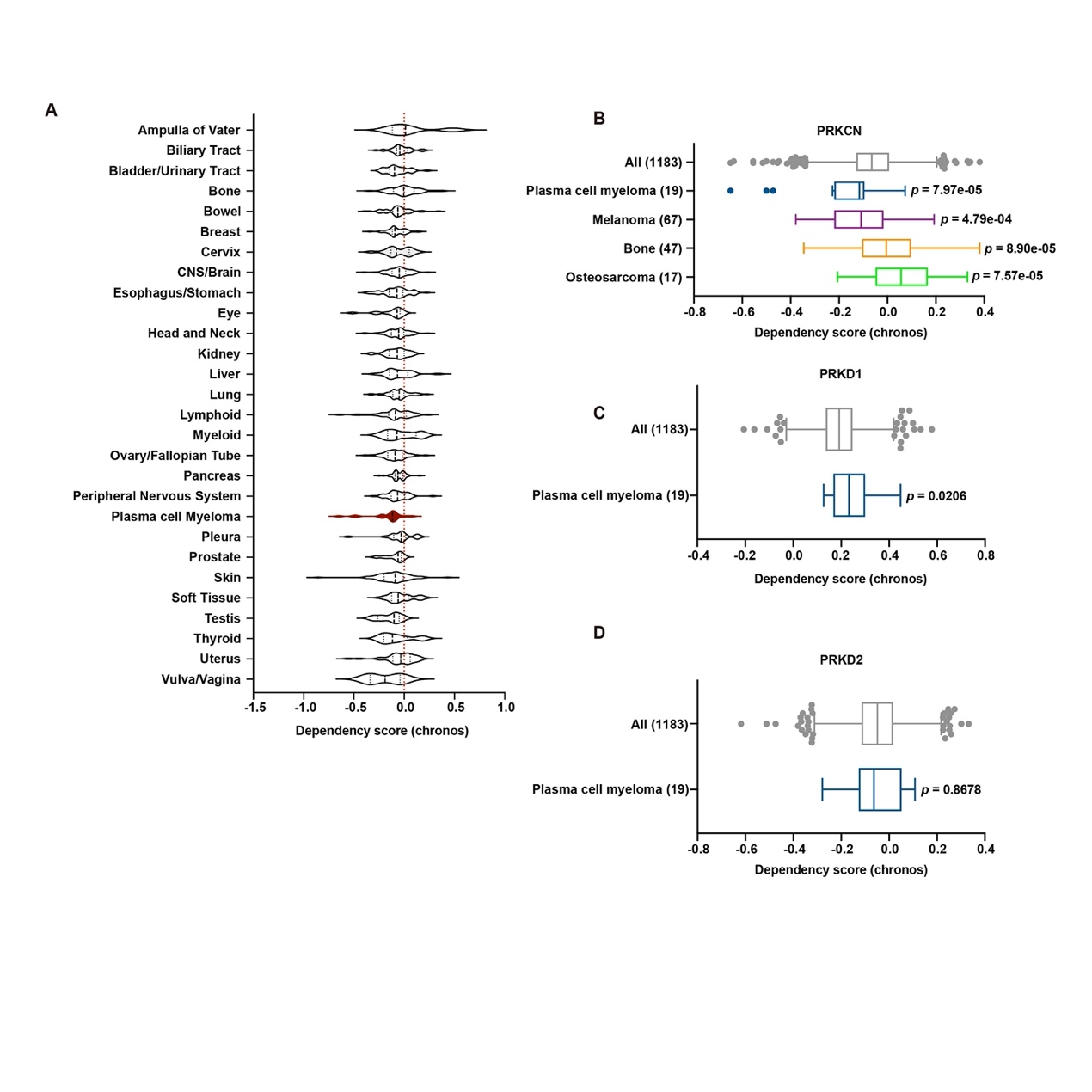


**Figure S2.** **Lineage-dependency analysis of PRKCN and its family members including PRKD1 and PRKD2** **in the DepMap portal.**

(A) Plot of CRISPR dependency scores for PRKCN by lineage using DepMap database (<https://depmap.org/portal/home/>). (B) Enriched lineages analysis depicting highest lineage dependency of PRKCN in plasma cell myeloma and additional three types of cell lines. Plasma cell myeloma (n = 19), Melanoma (n = 67), Bone (n = 47), Osteosarcoma (n = 17). A lower dependency score (chronos) indicates that the target gene of interest is essential in a given cell line. Enriched lineage is defined as *p* < 0.0005 compared with all other cell lines. The ranking is based on effect size in an ascending order. The middle line represents the median. Kruskal-Wallis test was performed. (C-D) Neither PRKD1 nor PRKD2 displays plasma cell myeloma-specific dependency (*p* > 0.0005). Note that all the myeloma cell lines have positive dependency scores for PRKD1. The middle line represents the median. Mann-Whitney test was performed.

**
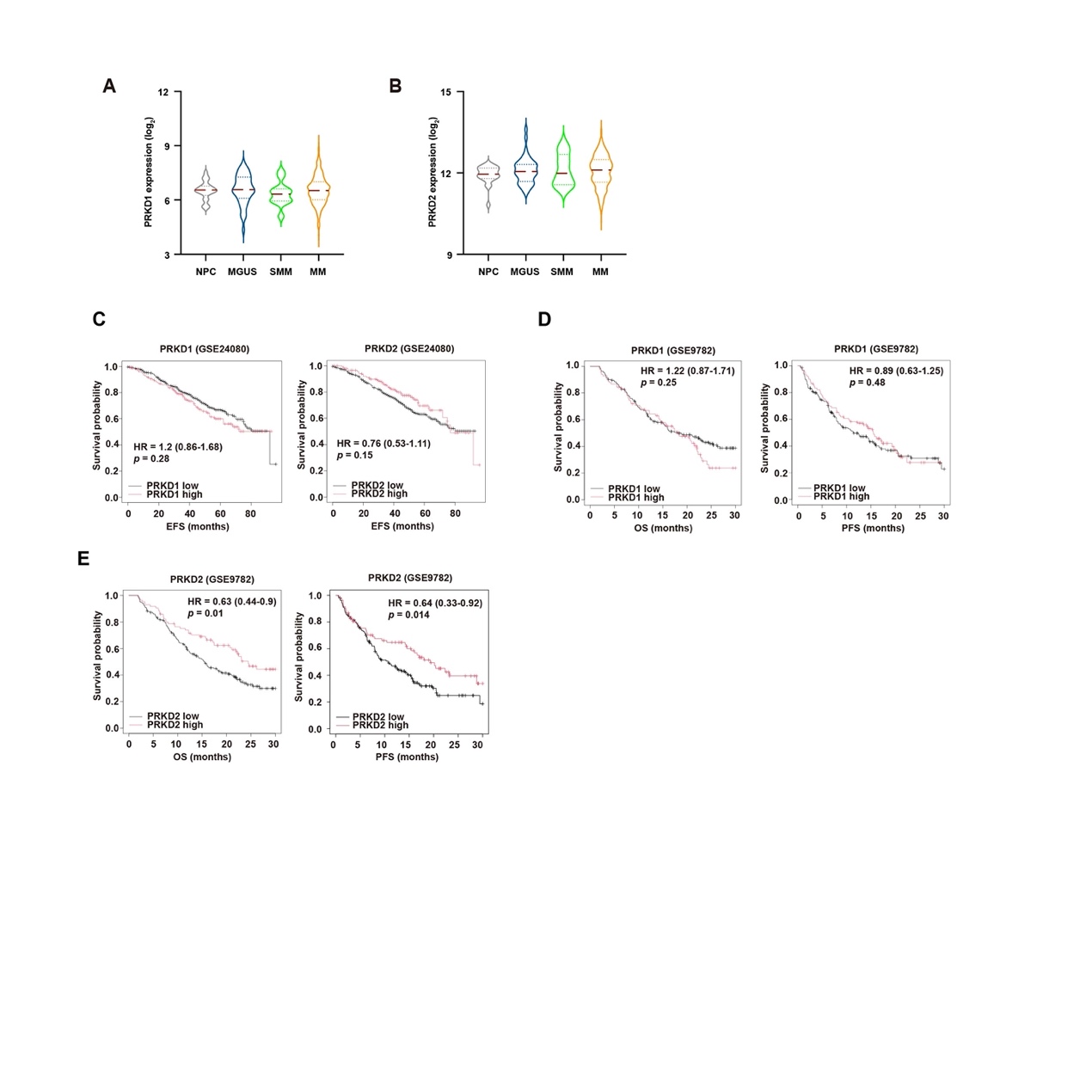
**

**Figure S3. Expression of PRKD1 and PRKD2 in MM patients and correlation between their expression with survival outcome of MM patients.**

(A-B) Violin plots comparing PRKD1 (A) or PRKD2 (B) expression in normal plasma cells (NPC, n = 22), monoclonal gammopathy of undetermined significance (MGUS, n = 44), smoldering multiple myeloma (SMM, n = 12) and MM patient samples (MM, n = 559) using GSE5900 and GSE2658 datasets. Note there is no significant difference in PRKD1 or PRKD2 expression across four groups. The middle line represents the median. Kruskal-Wallis test was performed. (C) Kaplan-Meier survival curves showing the association of PRKD1 and PRKD2 expression with the event free survival (EFS) probability of newly-diagnosed MM patients in GSE24080 dataset. (D-E) Kaplan-Meier survival curves showing the association of PRKD1 and PRKD2 expression with overall survival (OS) and progression-free survival (PFS) of refractory/relapsed MM patients using GSE9782 dataset.


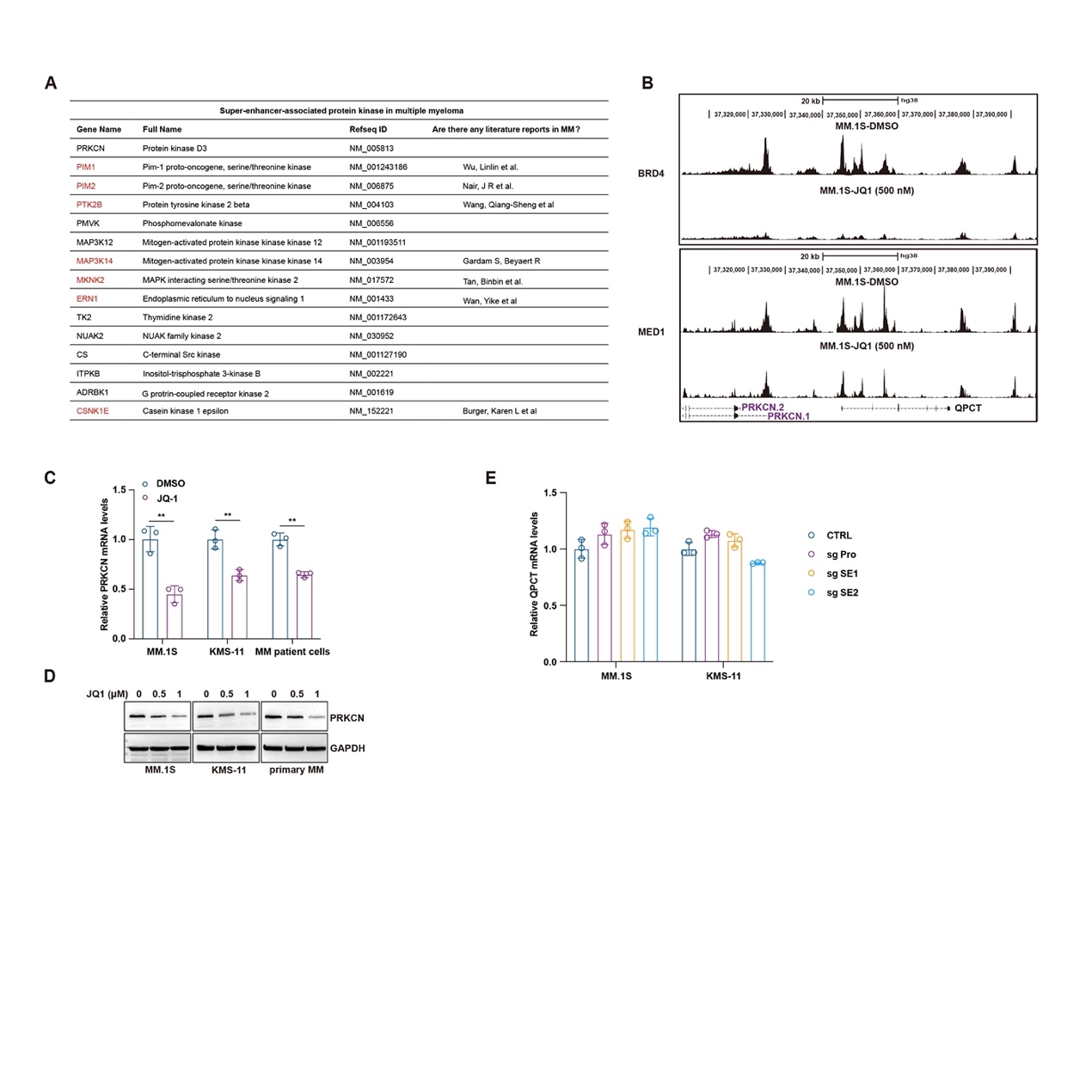


**Figure S4. Repression of SE activity weakens the expression of PRKCN in MM cells.**

(A) The candidate SEs-associated protein kinases in MM.1S cells were displayed in the table according to the publically-available ChIP-seq dataset (GSE44931). The protein kinases marked in red have been reported to contribute to the pathogenesis of MM in the literature.^[1-7]^ (B) The gene track of BRD4 (upper) and MED1 (lower) ChIP-seq occupancy at PRKCN SE in MM.1S cells following JQ1 treatment. (C-D) RT-qPCR and immunoblot analysis of PRKCN expression in MM.1S, KMS-11 or primary MM cells subjected to JQ1 treatment. For RT-qPCR, data are presented as mean ± SD. Unpaired two-tailed *t* test was performed, n = 3. (E) QPCT mRNA expression was not obviously changed in MM.1S and KMS-11 cells upon depression of SE activity by dCas9-CRAB CRISPRi with three individual sgRNAs (sgPRKCN Pro, sgPRKCN SE1 and sgPRKCN SE2). Data are presented as mean ± SD. One-way ANOVA with Dunnett's post hoc test was performed, n = 3.


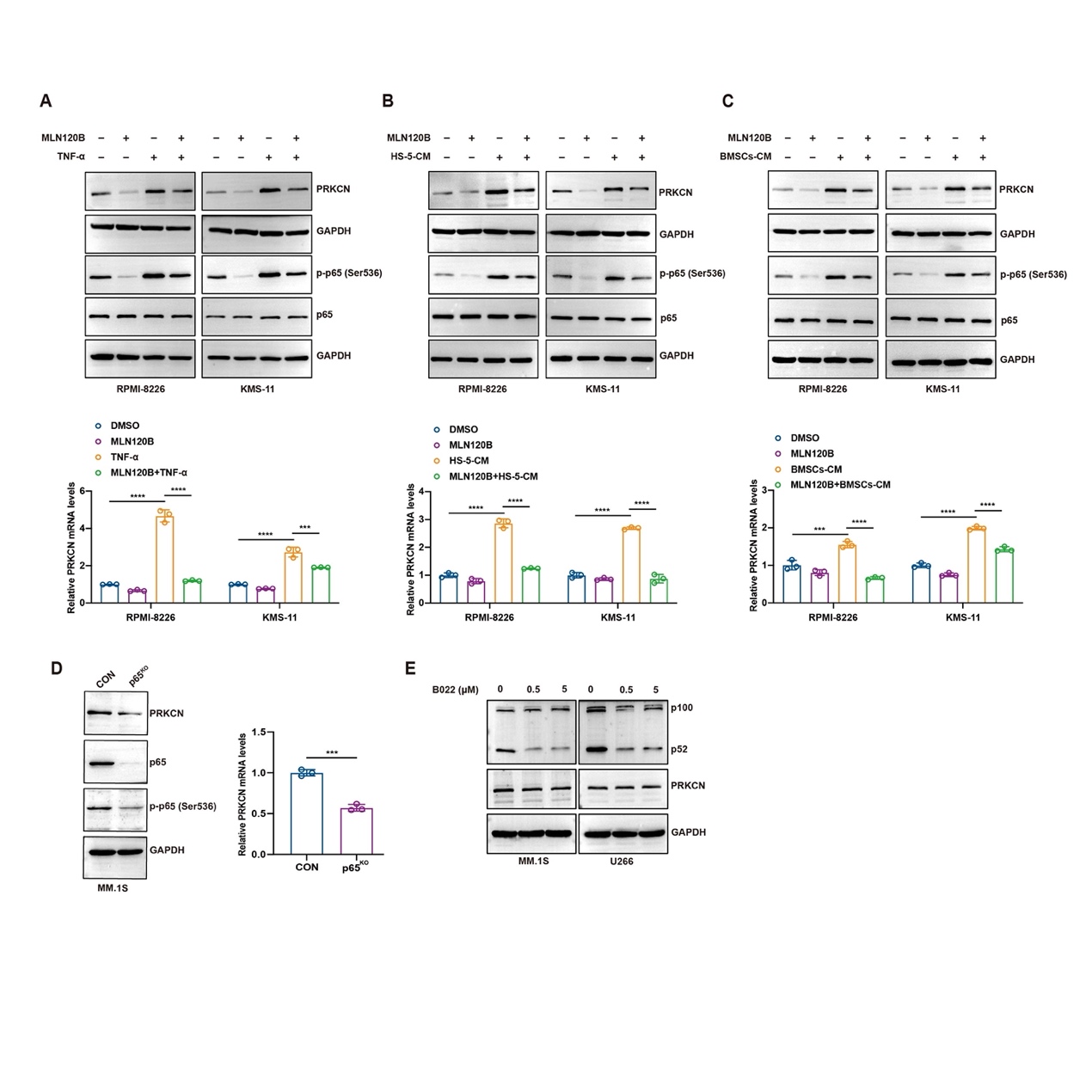


**Figure S5. PRKCN transcription is driven by canonical NF-κB signaling pathway in MM cells.**

(A-C) RPMI-8226 or KMS-11 cells were treated with TNF-α (A), HS-5-CM (B) or BMSCs-CM (C) in the presence or absence of IKKβ inhibitor MLN120B, and analyzed for protein expression of PRKCN, p65 and p-p65 by immunoblot analysis, and for PRKCN mRNA expression by RT-qPCR analysis. For RT-qPCR, data are presented as mean ± SD. One-way ANOVA with Tukey's post hoc test was performed, n = 3. (D) PRKCN, p65 and p-p65 protein expression (left), and PRKCN mRNA expression (right) were measured in MM.1S cells following knockout of p65 by CRISPR/Cas9. For RT-qPCR, data are presented as mean ± SD. Unpaired two-tailed *t* test was performed, n = 3. (E) MM.1S and U266 cells were treated with indicated doses of B022 for 72 h, followed by immunoblot analysis of p100/p52 and PRKCN.

**
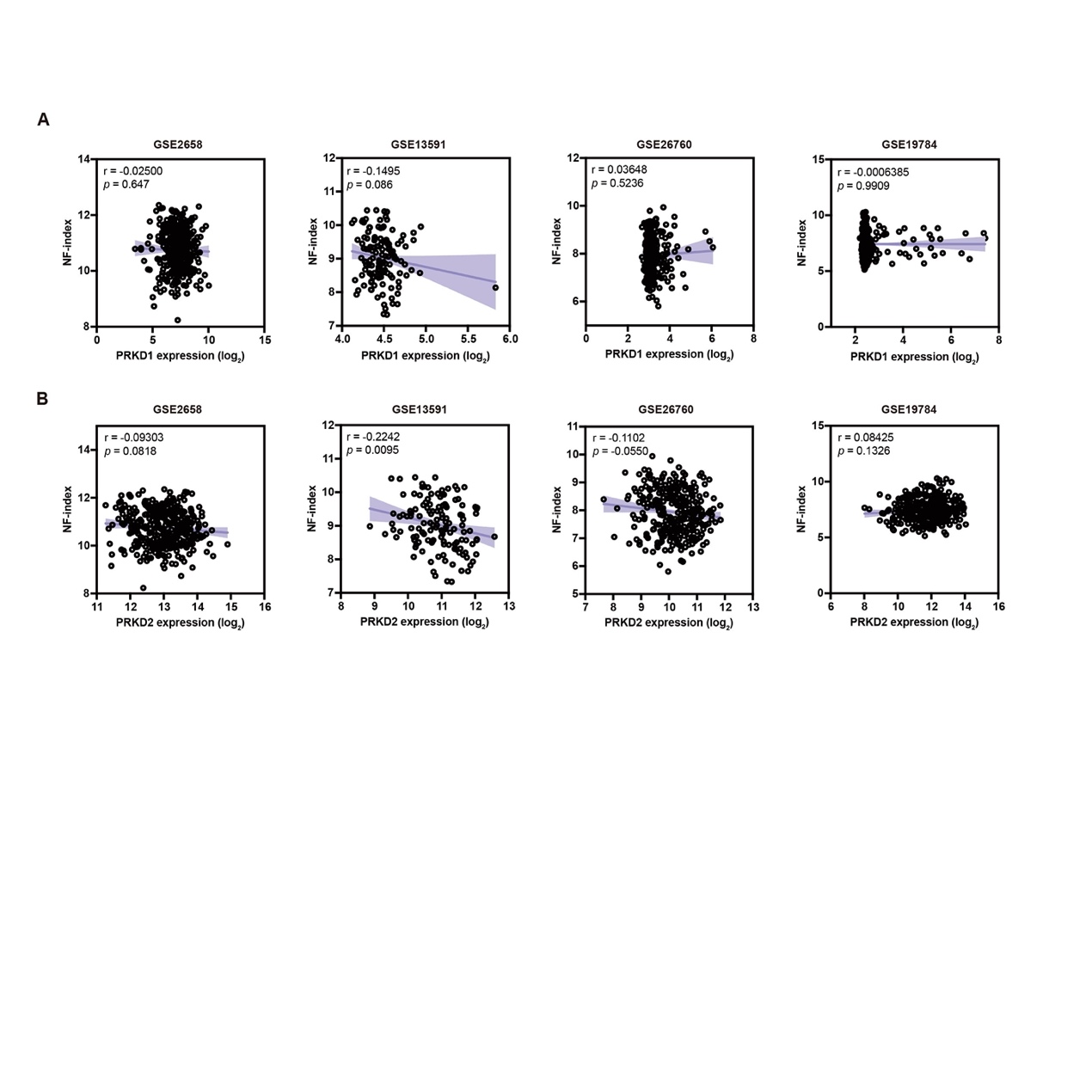
**

**Figure S6. No positive correlation exists between PRKD1 or PRKD2 mRNA expression with NF-κB index in primary MM samples.**

Spearman's correlation analysis of the expression of PRKD1 (A) or PRKD2 (B) mRNA with NF-κB index in MM patients using multiple GEO datasets, including GSE2658, GSE13591, GSE26760 and GSE19784.


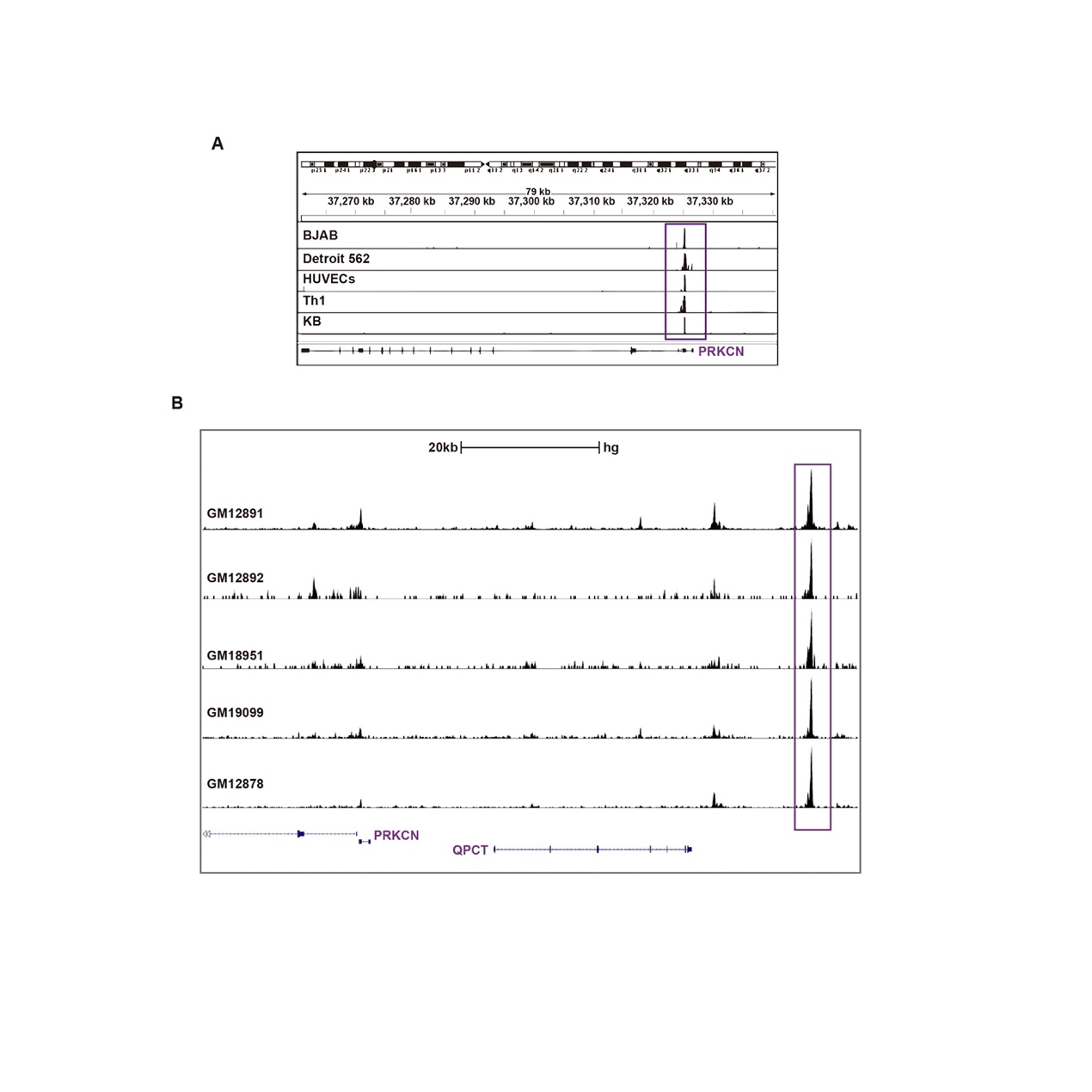


**Figure S7. The map of RelA/p65 subunit binding to the proximal promoter or distant SE region of PRKCN.**

(A) The corresponding ChIP-seq data retrieved from SRA databases (SRX4401064, SRX2404142, SRX4947721, SRX1460847 and SRX378805) were visualized by IGV software to illustrate p65 binding to PRKCN proximal promoter. (B) The corresponding ChIP-seq data from SRA databases (SRX150605, SRX017882, SRX017894, SRX017872 and SRX017906) were visualized by UCSC Brower to illustrate p65 binding to distant enhancer region of PRKCN.

**
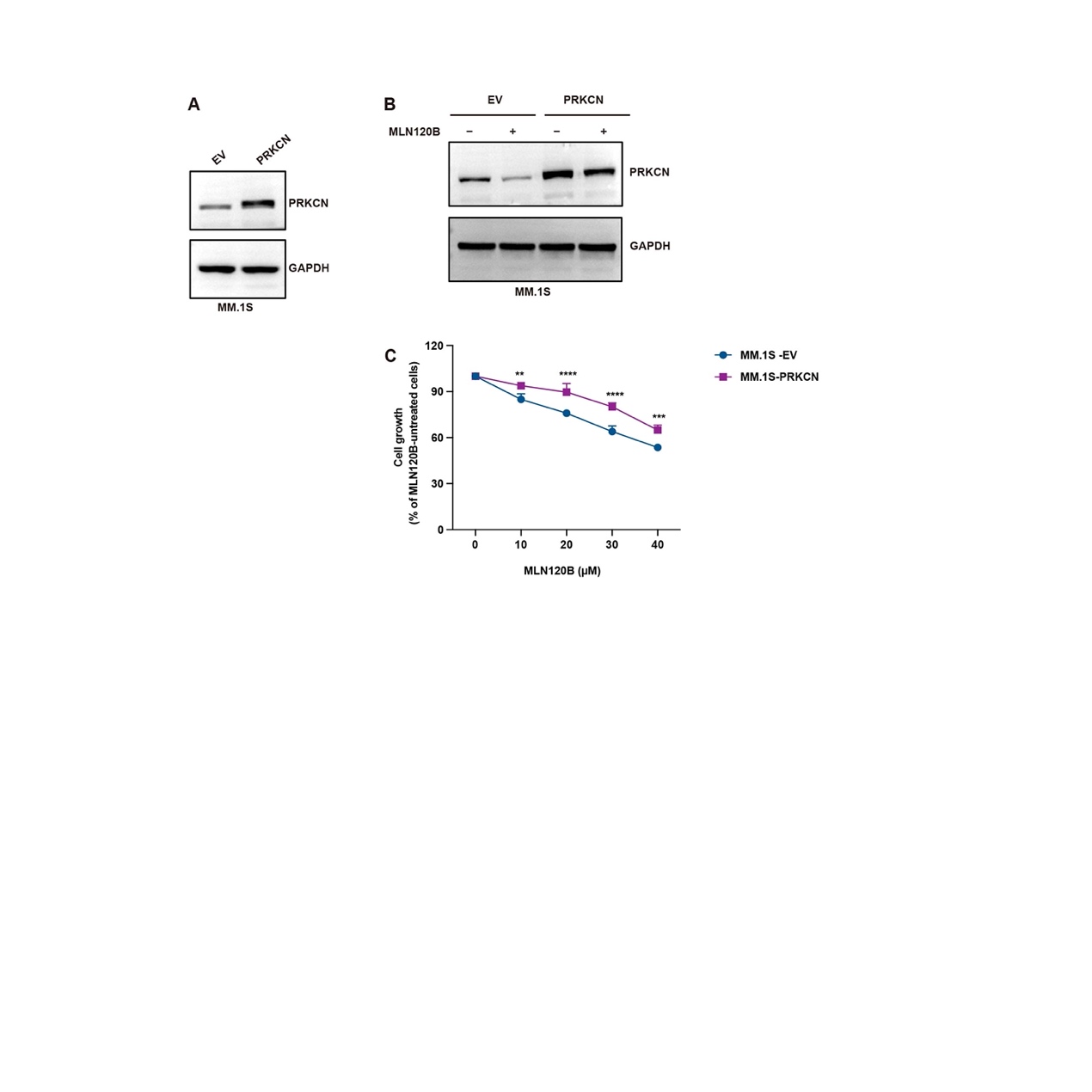
**

**Figure S8. PRKCN overexpression partly rescues MM.1S cells from growth inhibition induced by blockade of NF-κB signaling with MLN120B.**

(A) MM.1S cells were transduced with PRKCN or Venus lentiviruses, and cell lysates were subjected to immunoblot analysis to detect the PRKCN expression. (B) PRKCN protein levels were analyzed in MM.1S-EV or MM.1S-PRKCN cells treated with MLN120B for 72 h. (C) MM.1S-EV or MM.1S-PRKCN cells were exposed to various doses of MLN120B for 96 h, and cell viability was measured by CCK8 assay. Data are presented as mean ± SD. Two-way ANOVA with Bonferroni's post hoc test was performed, n = 3.


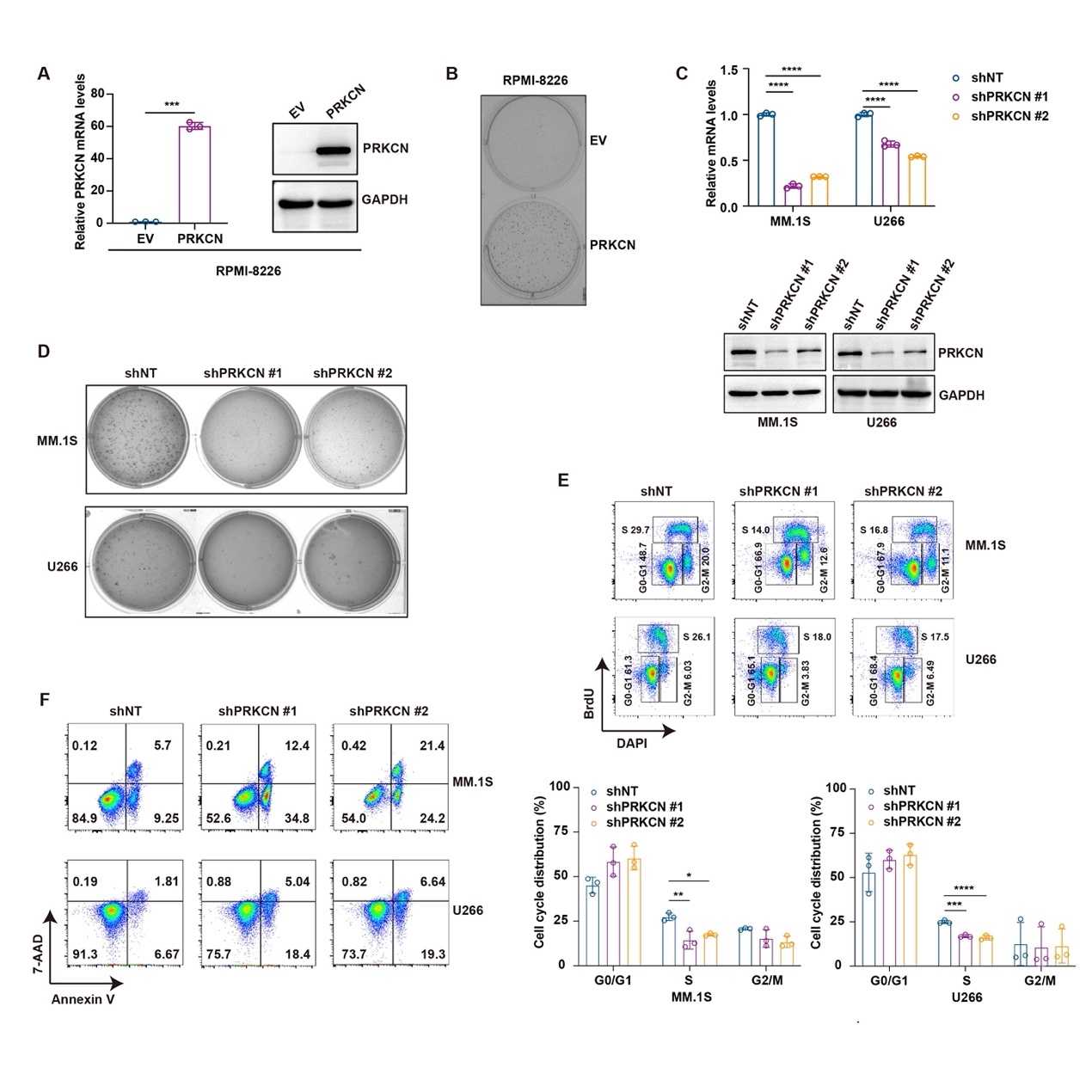


**Figure S9. PRKCN accelerates cell-cycle progression, promotes colony formation and inhibits apoptosis in MM cells.**

(A) PRKCN mRNA and protein expression levels in RPMI-8226-EV cells and RPMI-8226-PRKCN cells were assessed by RT-qPCR (left) and immunoblot (right) analysis, respectively. For RT-qPCR, data are presented as mean ± SD. Two-tailed Welch's *t* test was performed, n = 3. (B) Representative images of colony formation for RPMI-8226-EV and RPMI-8226-PRKCN cells. (C) MM.1S or U266 cells were transduced with shPRKCN (shPRKCN #1 and shPRKCN #2) or shscramble lentivirus (negative control, shNT) for 3 days, and analyzed for PRKCN expression by RT-qPCR (upper) and immunoblot (lower) analysis. (D) Representative images of colony formation in the soft agar for MM.1S or U266 cells subjected to PRKCN knockdown. (E) The results of BrdU/DAPI staining for detecting cell cycle distribution in MM.1S or U266 cells subjected to PRKCN knockdown. (F) The representative images of Annexin V/7-AAD staining for assessing apoptosis in MM.1S or U266 cells undergoing PRKCN knockdown. Data are presented as mean ± SD (C, E). One-way ANOVA with Dunnett's post hoc test was performed, n = 3.


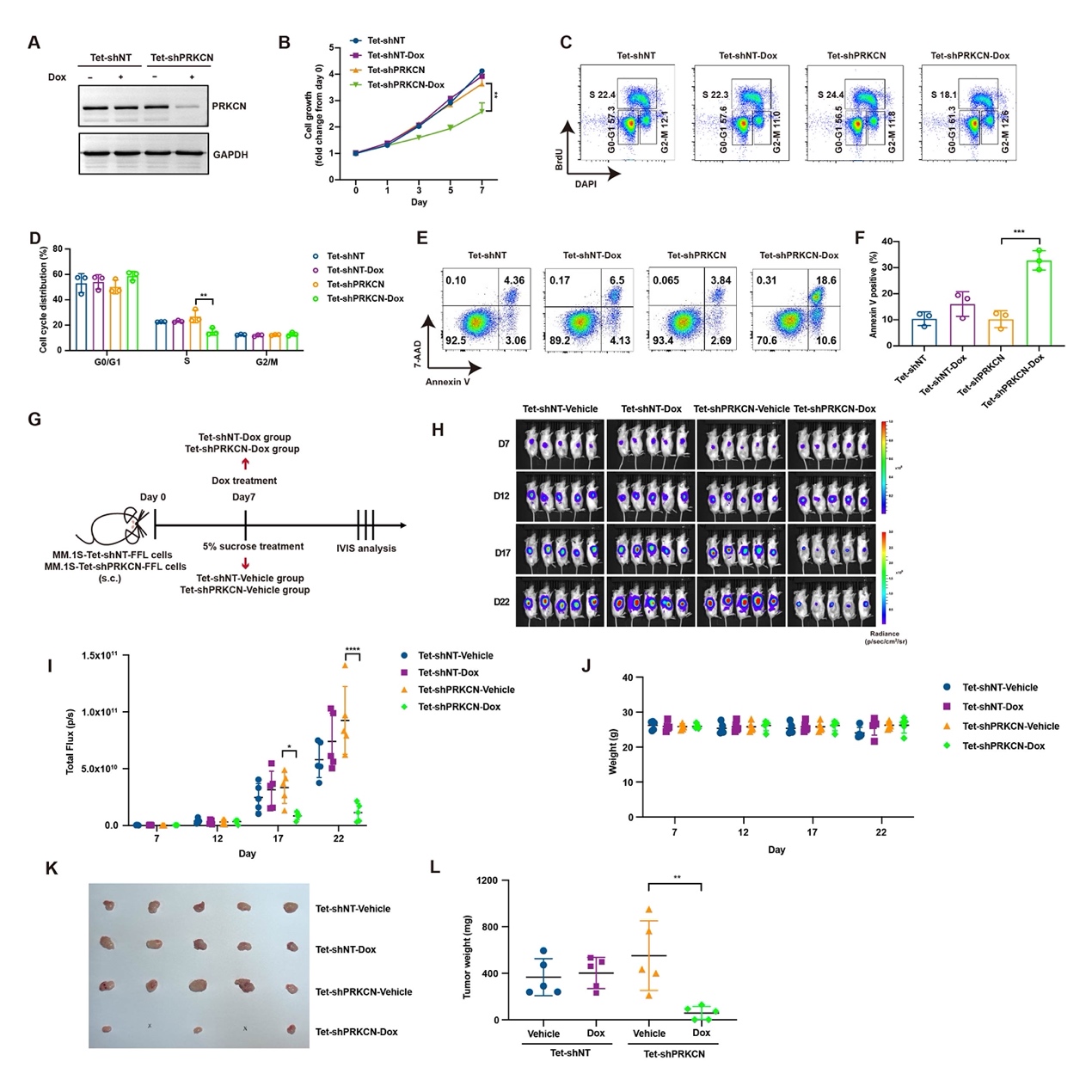


**Figure S10. Successful generation and *in vitro* or *in vivo* evaluation of MM.1S cells with doxycycline (Dox)-inducible PRKCN knockdown.**

(A) MM.1S cells were transduced with Tet-shNT or Tet-shPRKCN lentiviruses, and NGFR-positive cells were sorted for expansion. The resultant cells were treated with Dox (1 μg/mL) for 3 days to induce shRNA expression, and whole-cell lysates were analyzed for PRKCN protein expression. (B) MM.1S-Tet-shNT or MM.1S-Tet-shPRKCN stable cell lines were cultured in the presence Dox (1 μg/mL) for 3 days, which was designated as day 0. Cell proliferation was measured by CCK8 assay on days 0, 1, 3, 5, and 7. Data are presented as mean ± SD. Two-way ANOVA with Tukey's post hoc test was performed, n = 3. (C-F) MM.1S-Tet-shNT or MM.1S-Tet-shPRKCN stable cell lines were incubated with Dox (1 μg/mL) for 4 or 5 days, cell cycle analysis was performed by Brdu and DAPI staining (C-D), and apoptosis was assessed by Annexin V and 7-AAD staining (E-F). Representative results are shown in (C) and (E), and summarized results of 3 replications are shown in (D) and (F). Data are presented as mean ± SD (C, F). One-way ANOVA with Tukey's post hoc test was performed, n = 3. (G) Schematic representation of MM.1S xenograft mouse model with inducible PRKCN knockdown. MM.1S-Tet-shNT-FFL or MM.1S-Tet-shPRKCN-FFL cells were injected subcutaneously into NCG mice. One week after implantation, the mice received Dox (l mg/ml in 5% sucrose) or vehicle (5% sucrose) via drinking water for the duration of study. (H) Representative in vivo bioluminescence images of xenograft tumors in NCG mice at different time points. (I) Quantitative analysis of whole-body bioluminescence intensity at different time points. (J) Analysis of mice weight at different time points. (K-L) Tumors were photographed (K) and tumor weight (L) were quantified at the endpoint. Data are presented as mean ± SD. Data are presented as mean ± SD. One-way ANOVA with Tukey's post hoc test was performed, n = 5**
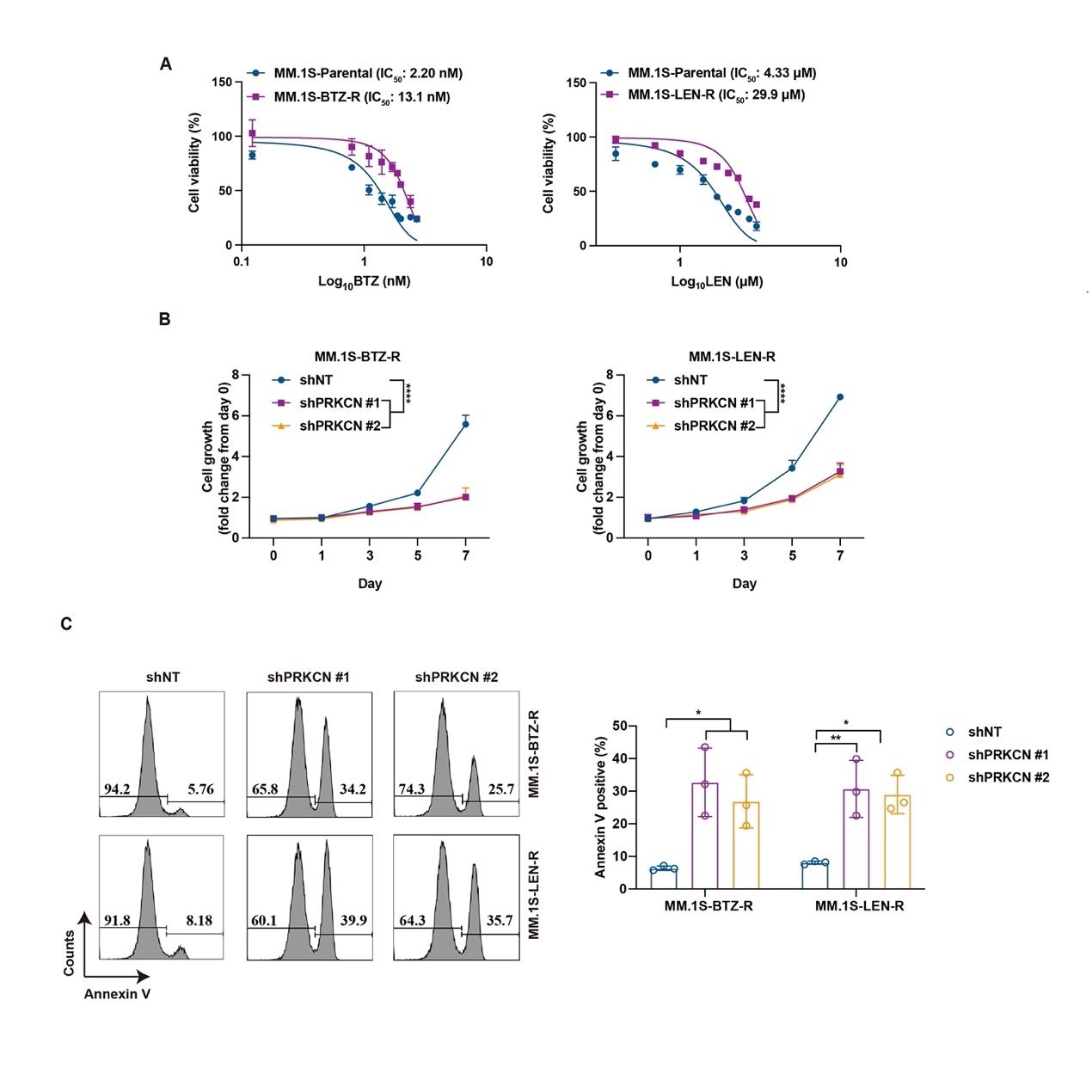
Figure S11. Loss of PRKCN weakens cell growth and induces apparent apoptosis in BTZ- and LEN- resistant MM.1S cells.**

(A) The viability of MM.1S-BTZ-R (left) or MM.1S-LEN-R (right) cells was measured by CCK8 assay. The IC₅₀ for bortezomib increased from 2.20 nM in parental cells to 13.1 nM in MM.1S-BTZ-R cells (resistance index = 5.96). Similarly, the IC₅₀ of lenalidomide from 4.33 μM to 29.9 μM in MM.1S-LEN-R cells (resistance index = 6.91). (B) MM.1S-BTZ-R cells or MM.1S-LEN-R cells were transduced with shPRKCN or shNT lentiviruses. After 3 days of infection, which was designated as day 0, cells were reseeded and cell viability was detected on days 0, 1, 3, 5, and 7 by CCK-8 assay. Data are presented as mean ± SD. Two-way ANOVA with Tukey's post hoc test was performed, n = 3. (C) MM.1S-BTZ-R cells or MM.1S-LEN-R cells were transduced with shPRKCN or shNT lentivirus. After 7 days of infection, cells were analyzed for apoptosis by flow cytometer with Annexin V staining. Representative results are shown on the left, and summarized results of 3 replications are shown on the right. Data are presented as mean ± SD. One-way ANOVA with Dunnett's post hoc test was performed, n = 3.


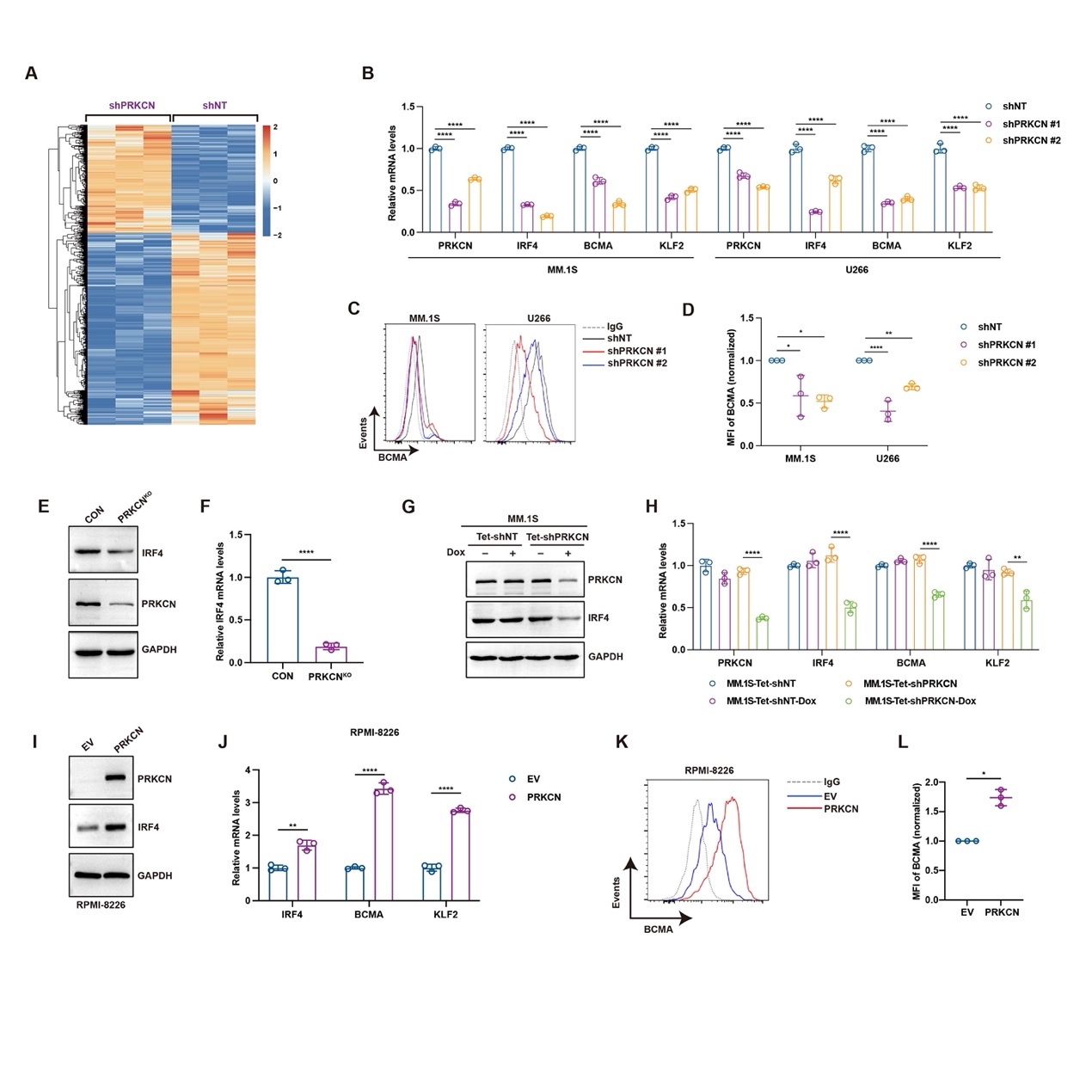


**Figure S12. PRKCN positively regulates expression of transcription factor IRF4 and its downstream targets including BCMA and KLF2 *in vitro*.**

(A) Heatmap of differently expressed genes between shNT group and shPRKCN group was shown according to q-value < 0.05 &| log_2_FC | > 0.58. (B) RT-qPCR analysis of PRKCN, IRF4, BCMA and KLF2 mRNA expression in MM.1S or U266 cells transduced with shPRKCN or shNT lentiviruses for 3 days. (C-D) Quantification of surface BCMA expression by flow cytometry analysis in MM.1S and U266 cells transduced with shPRKCN or shNT lentiviruses for 3 days. Representative results are shown in (C), and summarized results of 3 replications are shown in (D). MFI denoted mean fluorescent intensity. Data are presented as mean ± SD (B, D). One-way ANOVA with Dunnett's post hoc test was performed, n = 3. (E-F) Following knockout of PRKCN by CRISPR/Cas9 technique, MM.1S cells were analyzed for PRKCN and IRF4 protein expression by immunoblot analysis (E), and for IRF4 mRNA expression by RT-qPCR analysis (F). For RT-qPCR, data are presented as mean ± SD. Unpaired two-tailed *t* test was performed, n = 3. (G-H) MM.1S-Tet-shNT and MM.1S-Tet-shPRKCN cells treated with Dox (1 μg/ml) for 3 days, and cells were analyzed for PRKCN and IRF4 protein expression by immunoblot analysis (G), for PRKCN, IRF4, BCMA and KLF2 mRNA expression by RT-qPCR analysis (H). For RT-qPCR, data are presented as mean ± SD. One-way ANOVA with Tukey's post hoc test was performed, n = 3. (I) Immunoblot analysis of PRKCN and IRF4 expression levels in RPMI-8226-EV and RPMI-8226-PRKCN cells. (J) RT-qPCR analysis of IRF4, BCMA and KLF2 mRNA expression levels in RPMI-8226-EV and RPMI-8226-PRKCN cells. Data are presented as mean ± SD. Unpaired two-tailed *t* test was performed, n = 3. (K-L) Quantification of surface BCMA expression by flow cytometry analysis in RPMI-8226-EV and RPMI-8226-PRKCN cells. Data are presented as mean ± SD. Unpaired Welch's *t* test was performed, n = 3.


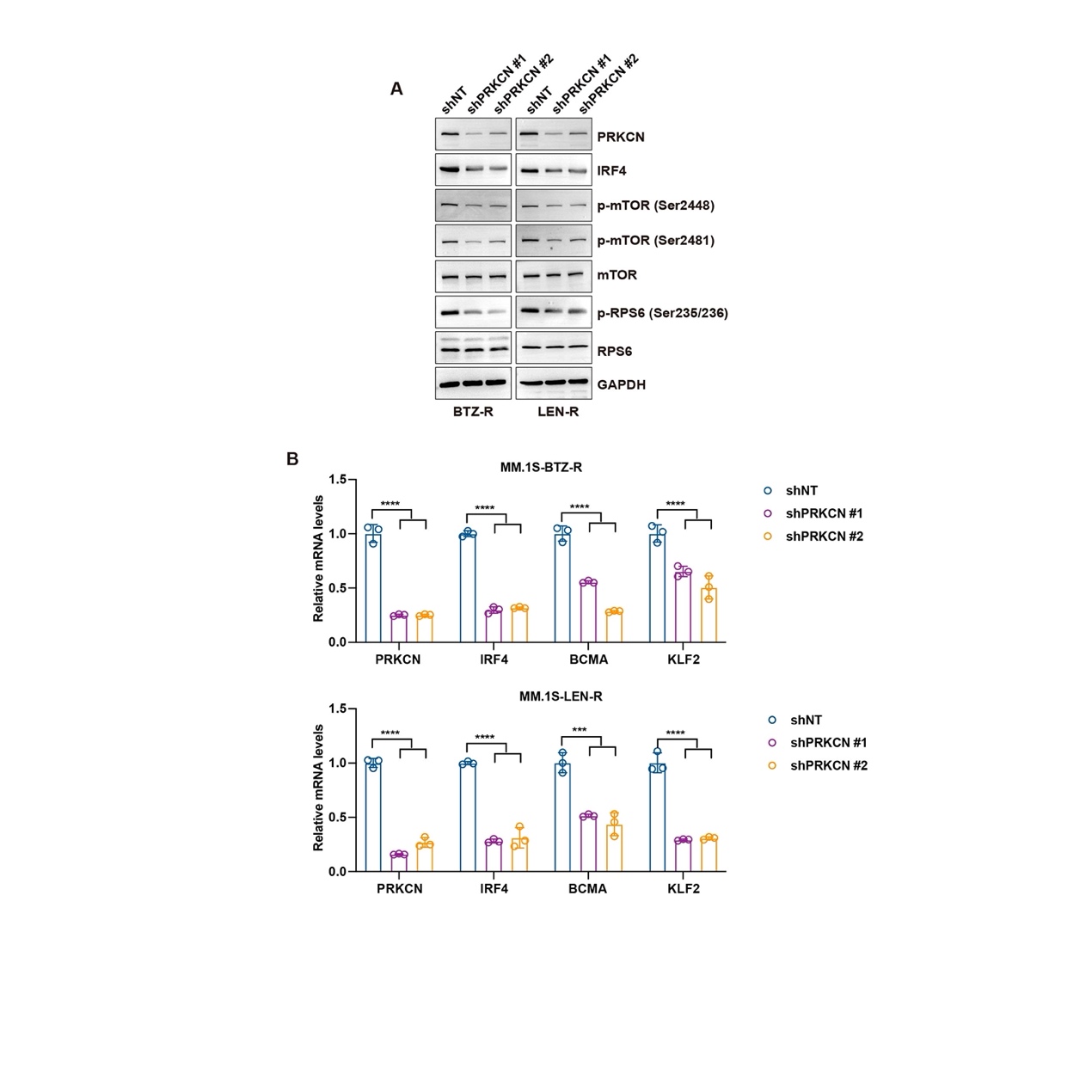


**Figure S13. Depletion of PRKCN** **dampens the mTORC1/C2-IRF4 axis** **in BTZ- or LEN-resistant MM.1S cells.**

(A) Immunoblot analysis of PRKCN, IRF4, mTOR, p-mTOR, RPS6 and p-RPS6 protein expression in MM.1S-BTZ-R and MM.1S-LEN-R cells following PRKCN knockdown. (B) RT-qPCR analysis of PRKCN, IRF4, BCMA and KLF2 mRNA expression in MM.1S-BTZ-R and MM.1S-LEN-R cells subjected to PRKCN knockdown. For RT-qPCR, data are presented as mean ± SD. One-way ANOVA with Dunnett's post hoc test was performed, n = 3.

**
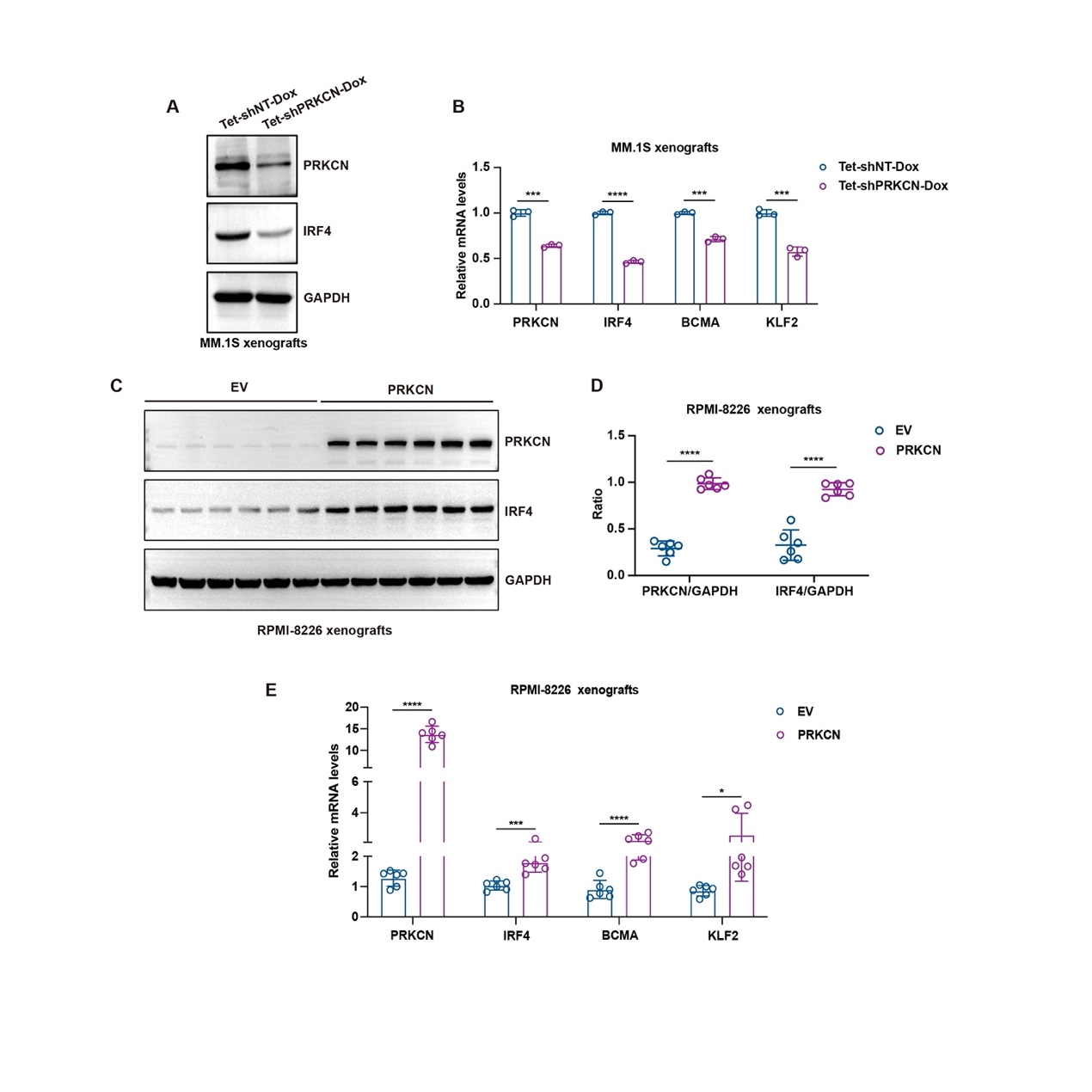
**

**Figure S14. PRKCN positively regulates expression of IRF4 and its downstream targets BCMA and KLF2 in the murine xenografts.**

(A) Immunoblot analysis of PRKCN and IRF4 in the dissected xenografts from MM.1S-Tet-shNT and MM.1S-Tet-shPRKCN cells. (B) RT-qPCR analysis of PRKCN, IRF4, BCMA and KLF2 in the xenografts from MM.1S-Tet-shNT and MM.1S-Tet-shPRKCN cells. Data are presented as mean ± SD. Unpaired two-tailed *t* test was performed, n = 3. (C-D) Immunoblot analysis of PRKCN and IRF4 in the xenografts derived from RPMI-8226-EV and RPMI-8226-PRKCN cells, and the graphs (D) summarize PRKCN and IRF4 band intensities normalized to GAPDH quantified via ImageJ. Data are presented as mean ± SD. Unpaired two-tailed *t* test was performed, n = 6. (E) RT-qPCR analysis of PRKCN, IRF4, BCMA and KLF2 expression in the xenografts from RPMI-8226-EV and RPMI-8226-PRKCN cells. Data are presented as mean ± SD. Unpaired two-tailed *t* test or unpaired Welch's *t* test was performed, n = 6.

**
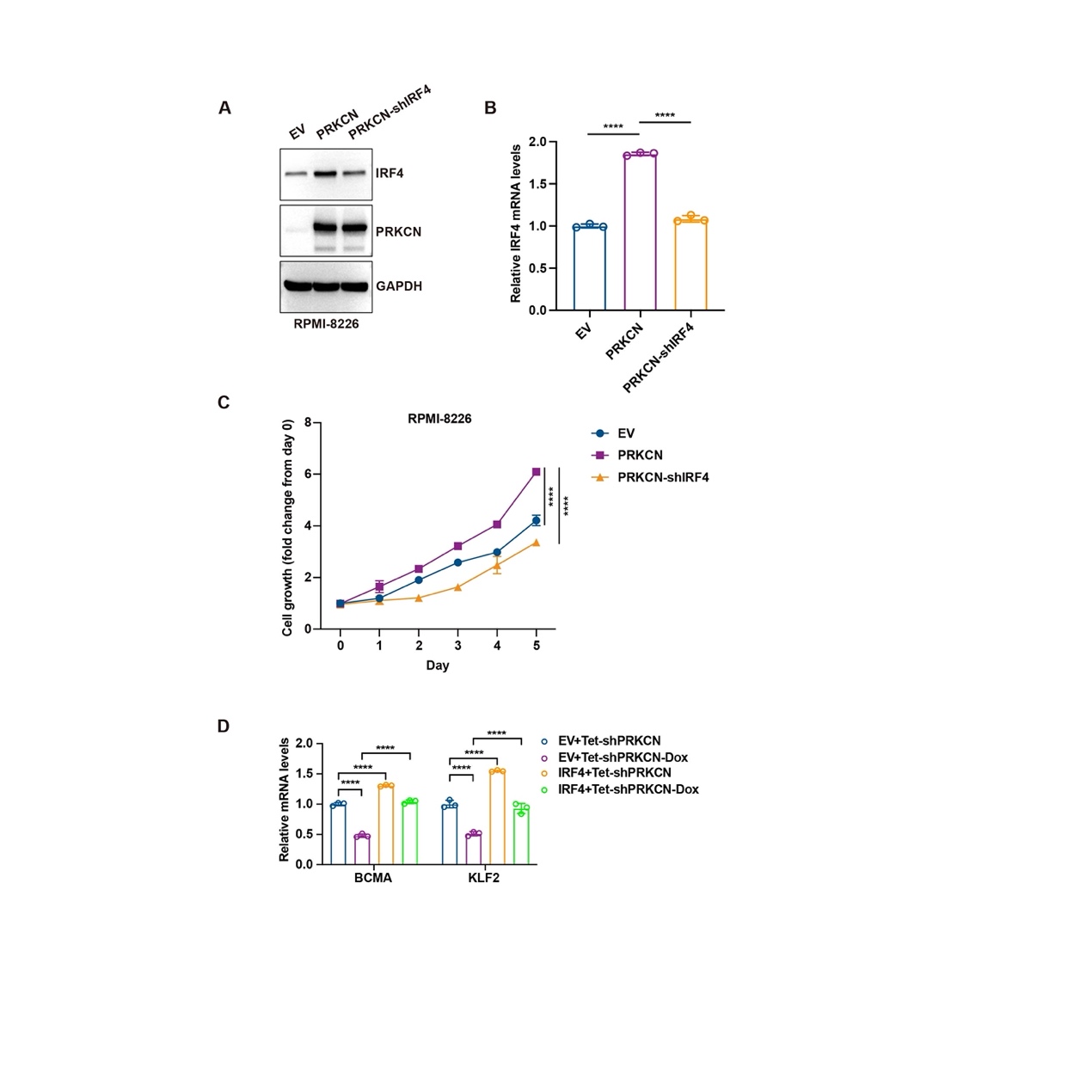
**

**Figure S15. The cell growth-promoting effect of PRKCN was markedly abrogated by IRF4 knockdown in RPMI-8226 cells.**

1. Immunoblot analysis of IRF4 and PRKCN expression in RPMI-8226-PRKCN cells transduced with or without shIRF4 lentiviruses. (B) RT-qPCR analysis of IRF4 mRNA expression in RPMI-8226-PRKCN cells transduced with or without shIRF4 lentiviruses. Data are presented as mean ± SD. One-way ANOVA with Tukey's post hoc test was performed, n = 3. (C) RPMI-8226-PRKCN cells transduced with or without shIRF4 lentiviruses were evaluated for cell viability by CCK8 assay. Data are presented as mean ± SD. Two-way ANOVA with Tukey's post hoc test was performed, n = 3. (D) RT-qPCR analysis of BCMA and KLF2 in MM.1S-Tet-shPRKCN cells transduced with Venus or IRF4-overexpressing lentiviruses in the presence or absence of Dox for 3 days. For RT-qPCR, data are presented as mean ± SD. One-way ANOVA with Tukey's post hoc test was performed, n = 3.


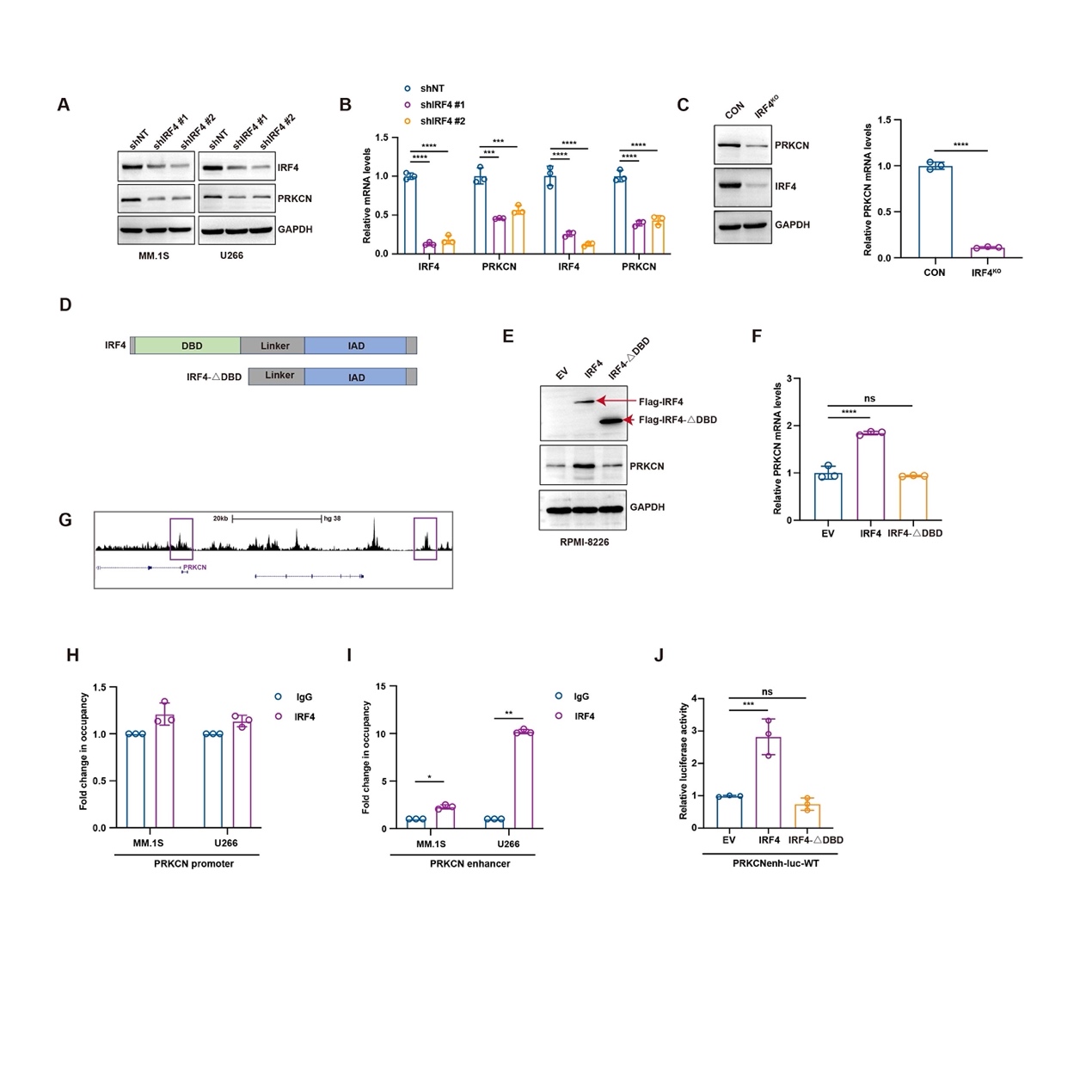


**Figure S16. PRKCN is directly transactivated by IRF4 in MM cells.**

(A-B) MM.1S or U266 cells were transduced with shIRF4 or shNT lentiviruses for 3 days, and analyzed for IRF4 and PRKCN expression by immunoblot analysis (A) and RT-qPCR analysis (B). Data are presented as mean ± SD. One-way ANOVA with Dunnett's post hoc test was performed, n = 3. (C) Immunoblot analysis of IRF4 and PRKCN expression (left) as well as RT-qPCR analysis of PRKCN expression (right) in MM.1S cells subjected to IRF4 knockout via CRISPR/Cas9 technique. For RT-qPCR, data are presented as mean ± SD. Unpaired two-tailed *t* test was performed, n = 3. (D) Schematic diagram of wild-type IRF4 and its truncated mutant lacking DNA-binding domain (IRF4-△DBD). (E-F) RPMI-8226 cells transduced with IRF4 or IRF4-△DBD lentiviruses were analyzed for PRKCN, IRF4 and IRF4-△DBD protein expression by immunoblot analysis, and for PRKCN mRNA expression by RT-qPCR analysis. For RT-qPCR, data are presented as mean ± SD. One-way ANOVA with Dunnett's post hoc test was performed, n = 3. (G) The corresponding ChIP-seq data from SRA database (SRX327760) were visualized by UCSC Genome Browser to illustrate IRF4 occupancy at PRKCN proximal promoter or SE region in MM.1S cells. (H-I) ChIP-qPCR analysis of IRF4 occupancy at the promoter (H) and super-enhancer (I) region of PRKCN in MM.1S and U266 cells. Data are presented as mean ± SD. Unpaired two-tailed Welch's *t* test was performed, n = 3. (J) Venus, Venus-IRF4 or Venus-IRF4-△DBD plasmid was co-transfected with PRKCNenh-luc-WT plasmid into HEK293T cells, and cell lysates were prepared for luciferase activity measurement. Data are presented as mean ± SD. One-way ANOVA with Dunnett's post hoc test was performed, n = 3.


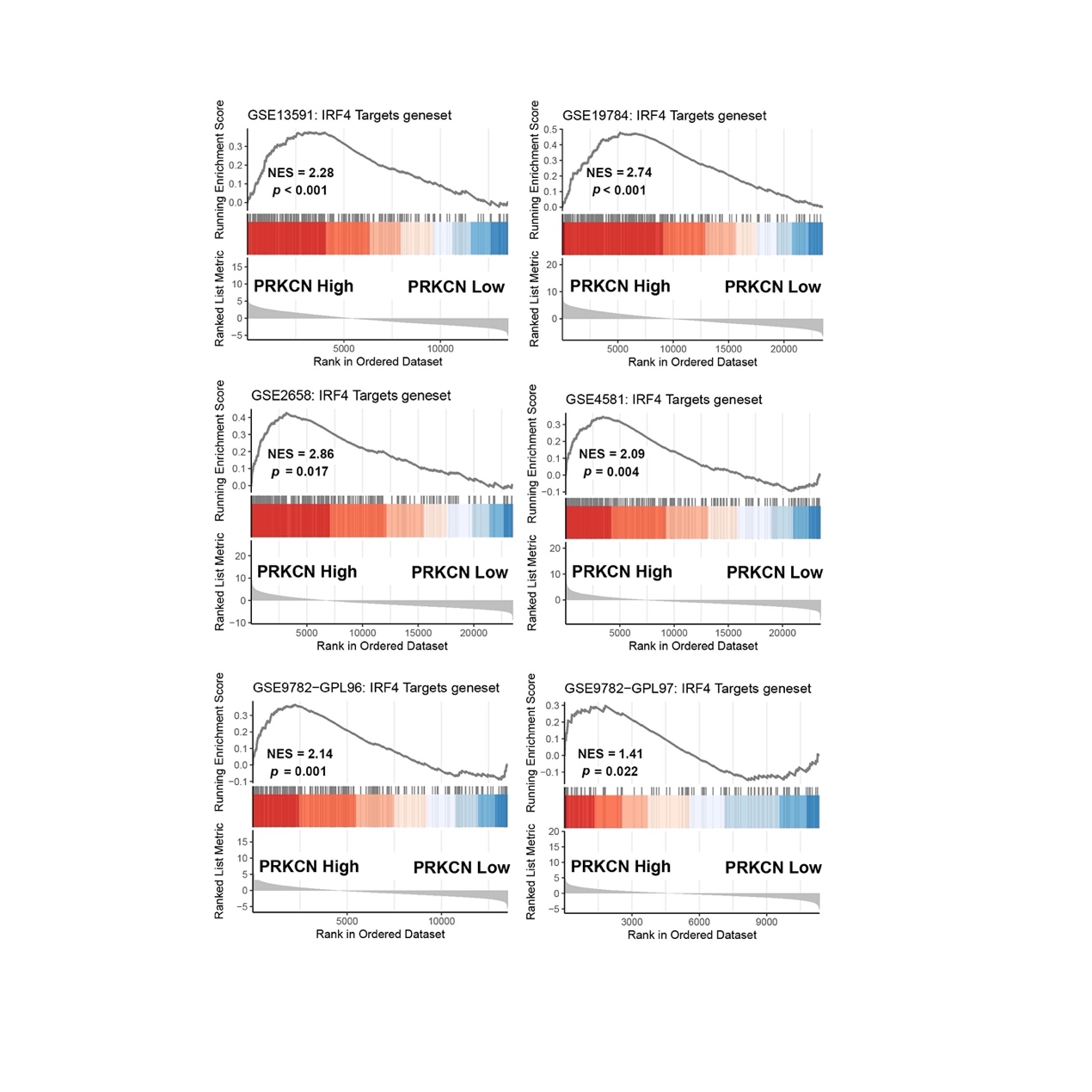


**Figure S17. Correlation between PRKCN expression and IRF4 target gene sets in MM patients.**

GSEA plots depicting consistent enrichment of IRF4 target gene set in MM patients with high PRKCN expression from five independent GEO datasets.


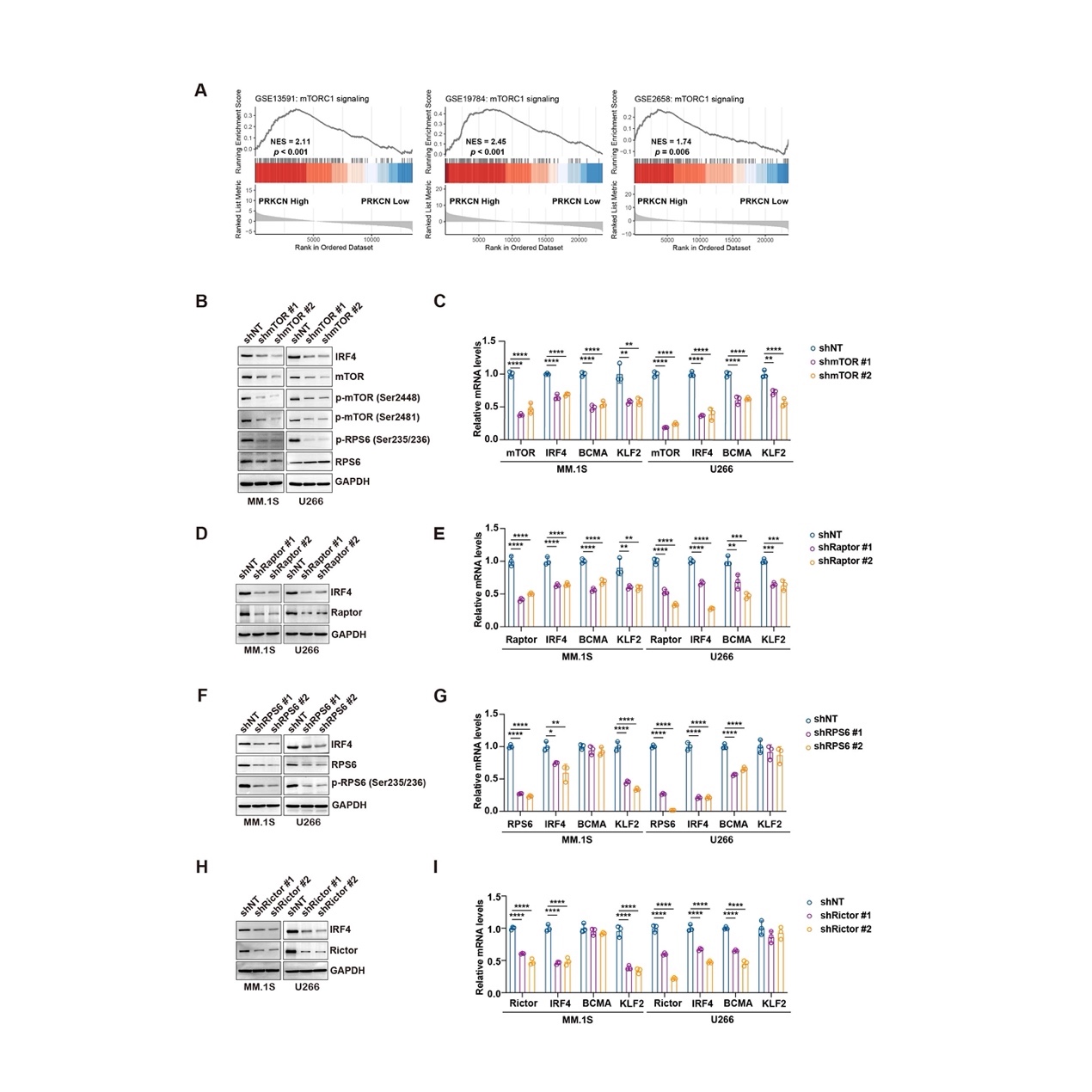


**Figure S18. IRF4 expression level is downregulated upon blockade of mTORC1 or mTORC2 signaling pathway in MM.1S and U266 cells.**

(A) GSEA plots depicting consistent correlation between PRKCN expression and mTORC1 signaling in MM patients across three independent GEO datasets. (B-C) MM.1S or U266 cells were transduced with mTOR specific shRNAs and analyzed for IRF4, mTOR, p-mTOR, RPS6 and p-RPS6 by immunoblot analysis (B), and for mTOR, IRF4, BCMA and KLF2 by RT-qPCR analysis (C). (D-E) MM.1S or U266 cells were transduced with Raptor specific shRNAs and analyzed for IRF4 and Raptor by immunoblot analysis (D), and for Raptor, IRF4, BCMA and KLF2 by RT-qPCR analysis (E). (F-G) MM.1S or U266 cells were transduced with RPS6 specific shRNAs and analyzed for IRF4, RPS6 and p-RPS6 by immunoblot analysis (F), and for RPS6, IRF4, BCMA and KLF2 RT-qPCR analysis (G). (H-I) MM.1S or U266 cells were transduced with Rictor specific shRNAs and analyzed for IRF4 and Rictor by immunoblot analysis (H), and for Rictor, IRF4, BCMA and KLF2 by RT-qPCR analysis (I). For RT-qPCR, data are presented as mean ± SD. One-way ANOVA with Dunnett's post hoc test was performed, n = 3.


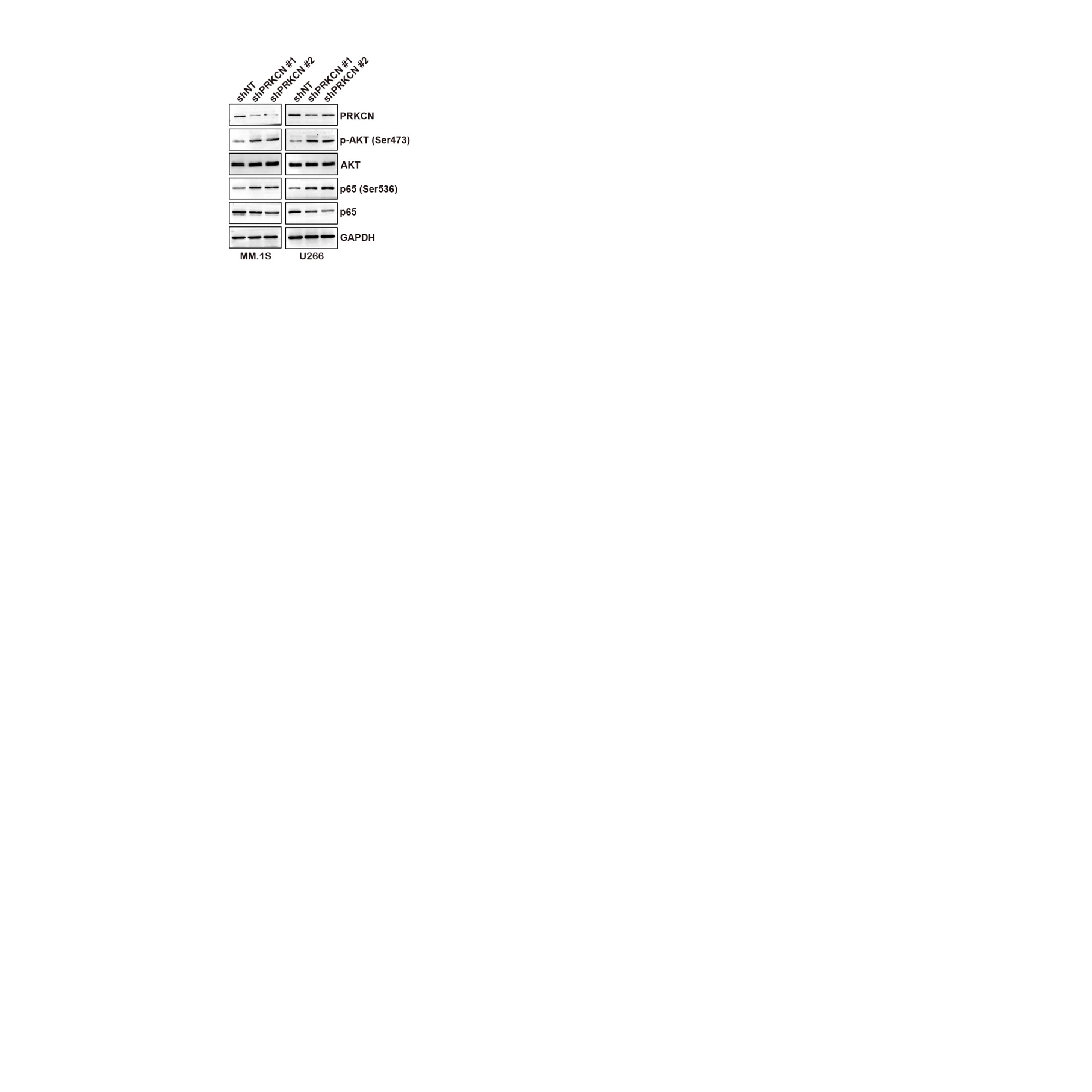


**Figure S19. Knockdown of PRKCN does not impede AKT or p65 activation in MM cells.**

MM.1S or U266 cells were transduced with shPRKCN or shNT lentiviruses for 3 days, and analyzed for PRKCN, p-AKT, AKT, p-p65 and p65 expression by immunoblot analysis.


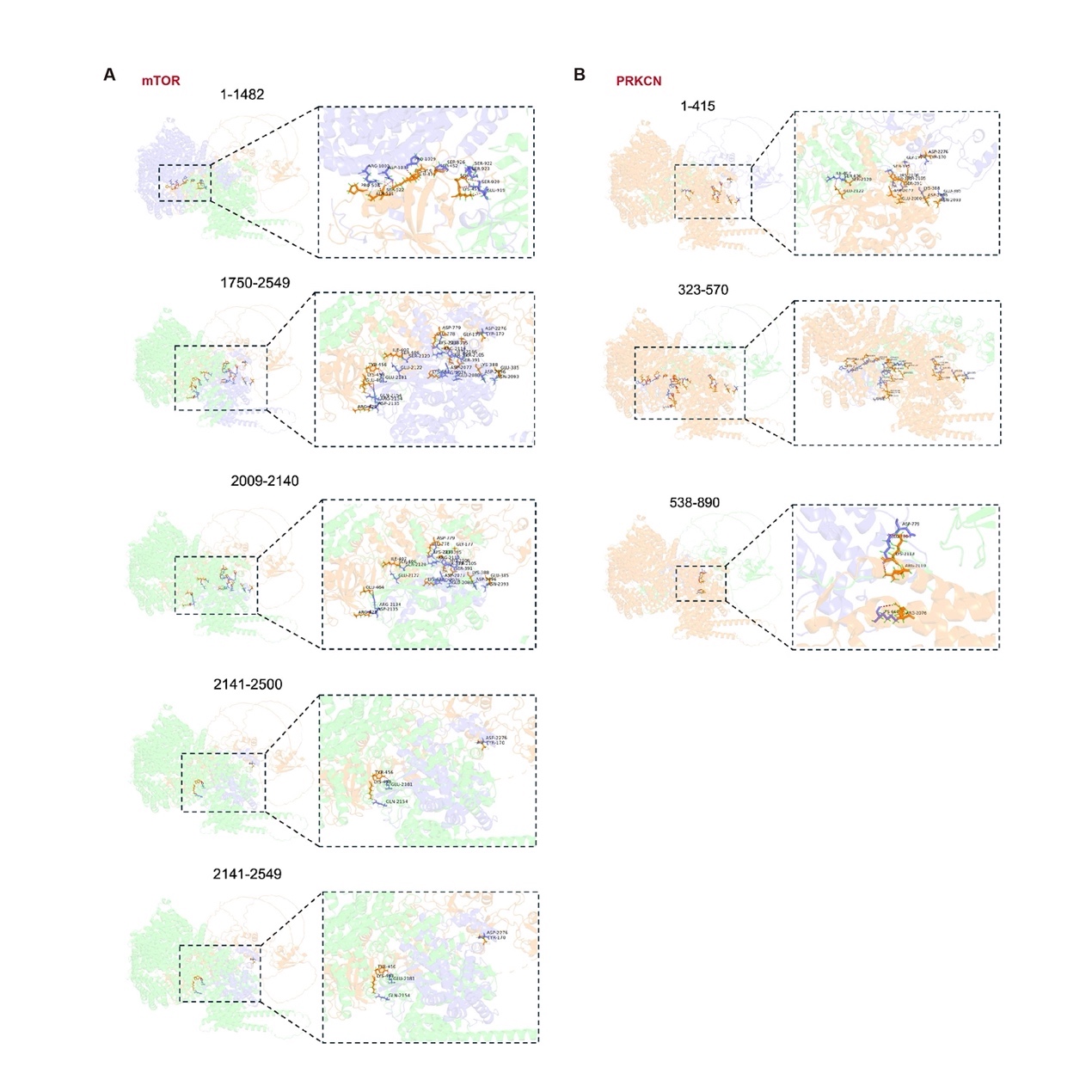


**Figure S20. Molecular docking predicts the potential binding sites within the specified domains of mTOR and PRKCN.**

(A) Cartoon representation of interactions between the main domains of mTOR (purple) with PRKCN (orange), where the interaction hotspot residues are labeled. On the right, from top to bottom, were the enlarged images for the interactions between the specified domains of mTOR and PRKCN. The binding sites of mTOR and PRKCN involved in the interaction were shown as purple stick models and orange stick models respectively. (B) Cartoon representation of interactions between the main domains of PRKCN (purple) with mTOR (orange), where the interaction hotspot residues are labeled. On the right, from top to bottom, were the enlarged images for the interactions between the specified domains of PRKCN and mTOR. The binding sites of PRKCN and mTOR involved in the interaction were shown as purple stick models and orange stick models respectively.

**
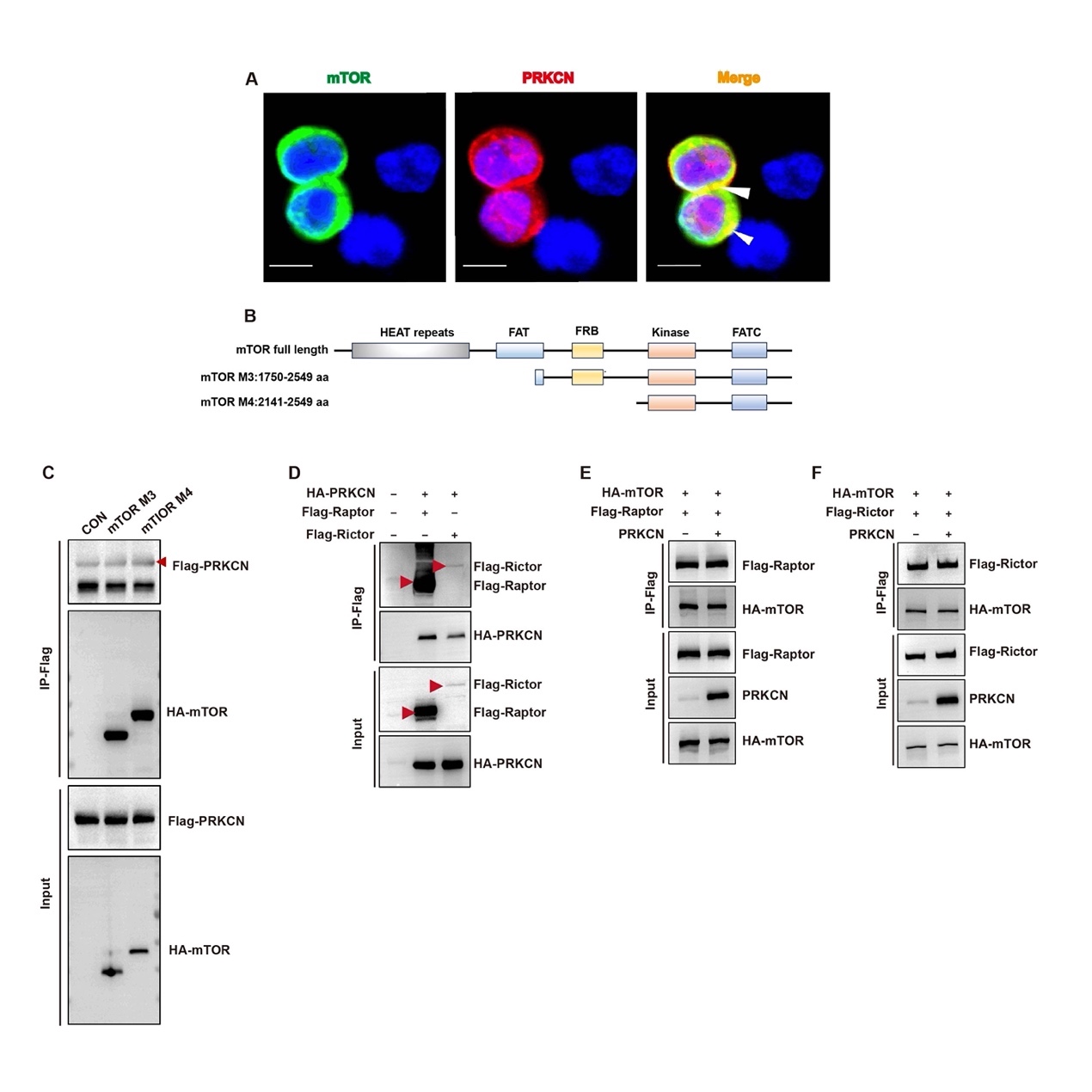
**

**Figure S21. PRKCN interacts with the mTORC1/C2 complex but does not influence mTOR binding to Raptor or Rictor.**

1. Confocal images of HEK293T cells co-transfected with HA-PRKCN and Flag-mTOR. Arrows indicate the co-localization of PRKCN with mTOR (Scale bars indicate 25 μm). (B) Schematic diagram of two additional mTOR truncated mutants. (C) Each indicated HA-mTOR mutant was co-transfected with Flag-PRKCN in HEK293T cells and subjected to co-immunoprecipitation analysis. (D) Immunoblot analysis of HA-PRKCN in anti-Flag-Raptor or anti-Flag-Rictor immunoprecipitates from HEK293T cells co-transfected with HA-PRKCN and Flag-Raptor or Flag-Rictor. (E-F) Immunoblot analysis of HA-mTOR in anti-Flag-Raptor (E) or anti-Flag-Rictor (F) immunoprecipitates from control or PRKCN-overexpressing HEK293T cells.


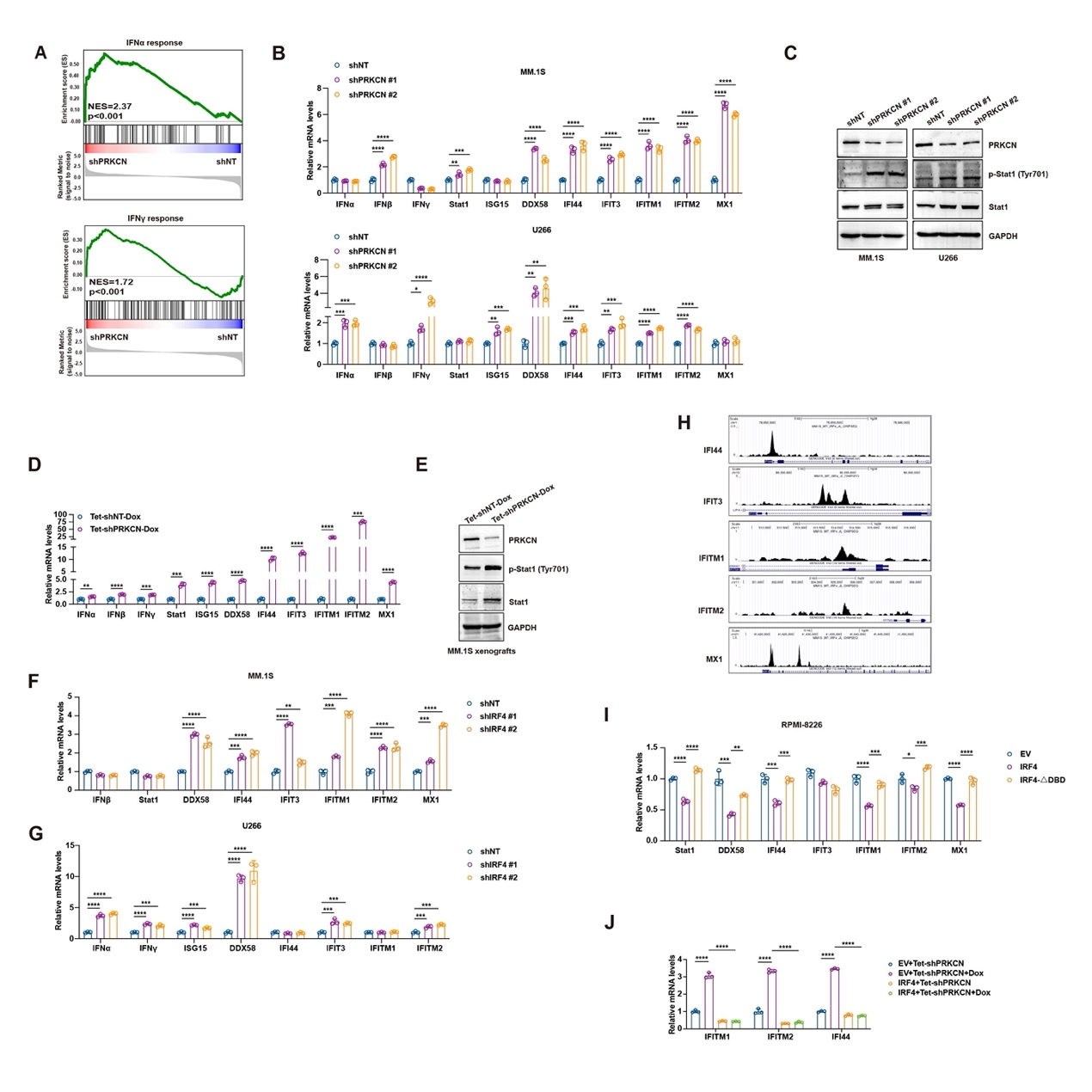


**Figure S22. PRKCN silencing evokes interferon signaling pathway partly though IRF4 downregulation and IRF4 represses transcription of ISGs by promoter binding.**

(A) GSEA plots of hallmark IFNα response and IFNγ response signaling in shPRKCN versus shNT cells. (B) The mRNA levels of IFNα, IFNβ, IFNγ, Stat1, ISG15, DDX58, IFI44, IFIT3, IFITM1, IFITM2 and MX1 by RT-qPCR analysis in MM.1S and U266 cells transduced with shPRKCN lentiviruses. Data are presented as mean ± SD. One-way ANOVA with Dunnett's post hoc test was performed, n = 3. (C) Immunoblot analysis of PRKCN, p-Stat1 and Stat1 in MM.1S and U266 cells transduced with shPRKCN lentiviruses. (D) RT-qPCR analysis of IFNα, IFNβ, IFNγ, Stat1, ISG15, DDX58, IFI44, IFIT3, IFITM1, IFITM2 and MX1 in the xenografts constructed by MM.1S-Tet-shNT and MM.1S-Tet-shPRKCN cells. Data are presented as mean ± SD. Unpaired two-tailed *t* test or unpaired two-tailed was Welch's *t* test performed, n = 3. (E) Immunoblot analysis of PRKCN, p-Stat1, and Stat1 in the dissected xenografts constructed by MM.1S-Tet-shNT and MM.1S-Tet-shPRKCN cells. (F-G) The mRNA levels of IFNβ, Stat1, DDX58, IFI44, IFIT3, IFITM1, IFITM2 and MX1 in MM.1S-shIRF4 cells, and IFNα, IFNγ, ISG15, DDX58, IFI44, IFIT3, IFITM1, and IFITM2 in U266-shIRF4 cells by RT-qPCR analysis. Data are presented as mean ± SD. One-way ANOVA with Dunnett's post hoc test was performed, n = 3. (H) The corresponding ChIP-seq data retrieved from SRA databases (SRX327760) were visualized by IGV software to illustrate IRF4 binding to IFI44, IFIT3, IFITM1, IFITM2 and MX1 promoter in MM.1S cells. (I) RT-qPCR analysis of Stat1, DDX58, IFI44, IFIT3, IFITM1, IFITM2 and MX1 in RPMI-8226 cells transduced with IRF4 or IRF4-△DBD lentiviruses. Data are presented as mean ± SD. One-way ANOVA with Tukey's post hoc test, n = 3. (J) The mRNA levels of IFITM1, IFITM2 and IFI44 in EV+Tet-shPRKCN cells and IRF4+Tet-shPRKCN cells cultured with Dox. Data are presented as mean ± SD. One-way ANOVA with Tukey's post hoc test, n = 3.


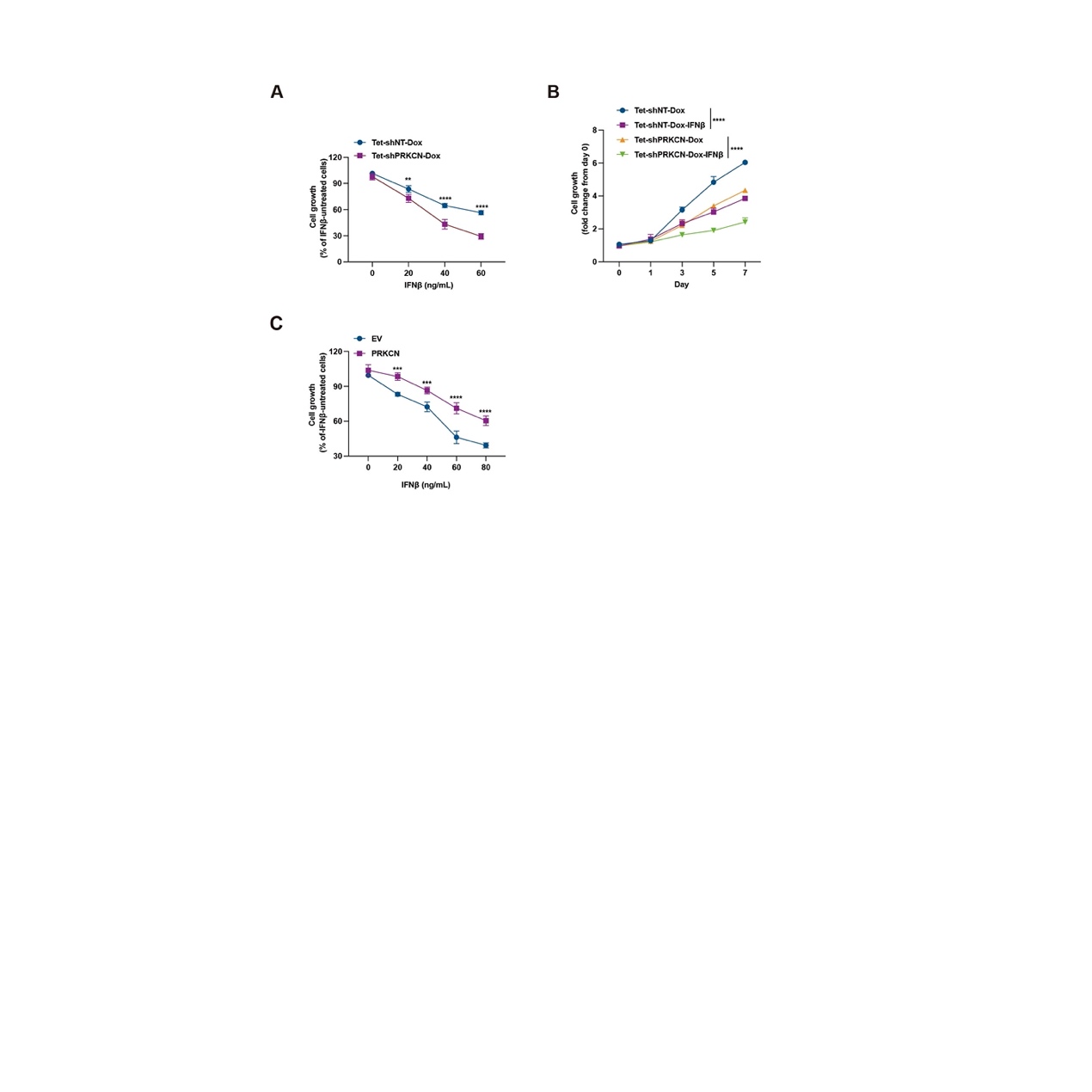


**Figure S23. PRKCN silencing confers increased susceptibility to IFN treatment.**

(A-B) Cell viability was measured by CCK8 assay in MM.1S-Tet-shNT and MM.1S-Tet-shPRKCN cells cultured in the presence of Dox with or without IFNβ. (C) RPMI-8226-EV or RPMI-8226-PRKCN cells were cultured with or without IFNβ and cell viability was assessed by CCK8 assay. Data are presented as mean ± SD (A-C). Two-way ANOVA with Šídák's post hoc test (A, C), and two-way ANOVA with Tukey’s post hoc test (B) were performed, n = 3.


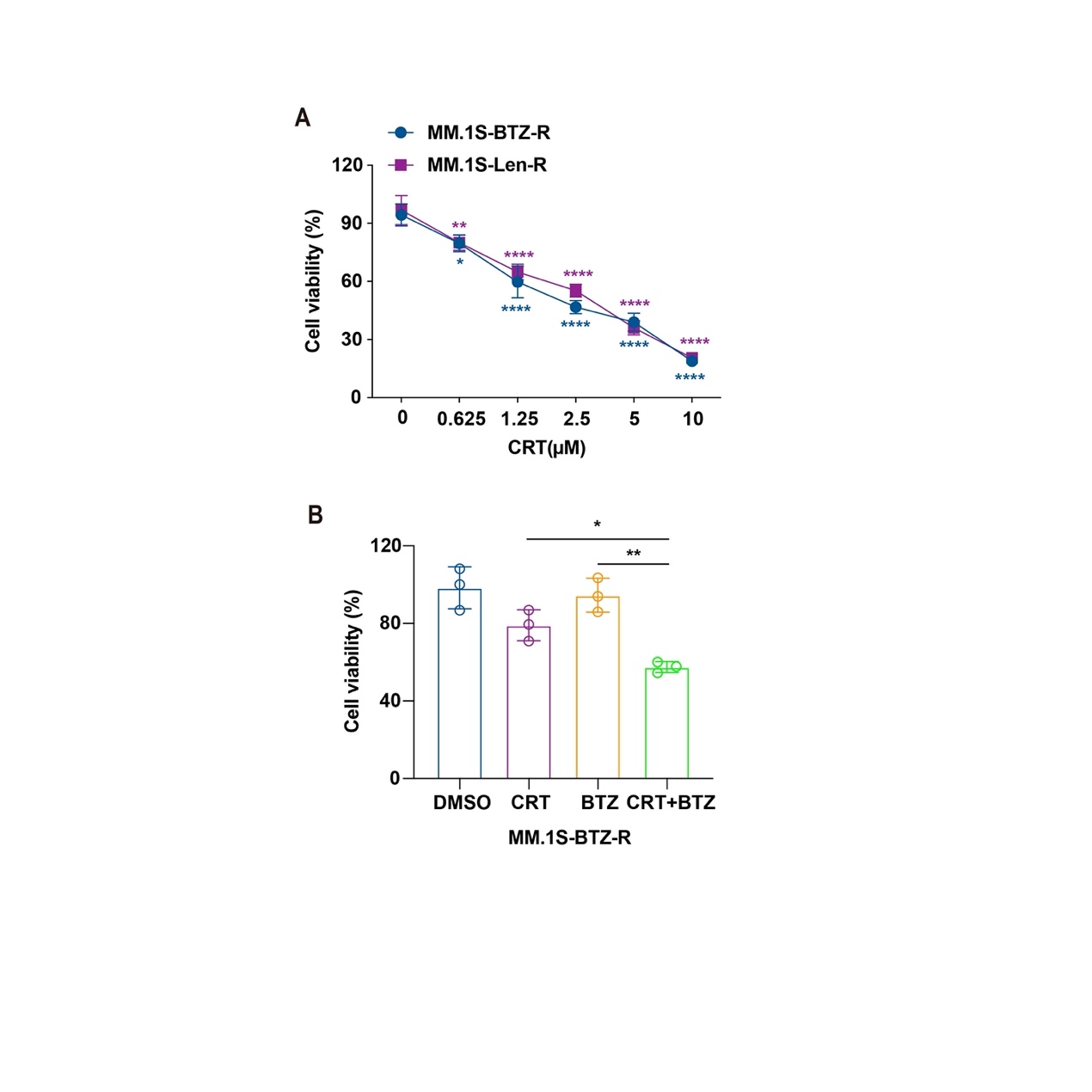


**Figure S24.** **CRT0066101 (CRT) treatment weakens the in vitro viability of BTZ- or LEN-resistant MM.1S cells.**

(A) The sensitivity of BTZ- and LEN-resistant MM.1S cells to different concentrations of CRT was detected by CCK8 assay. (B) Cell viability of MM.1S-BTZ-R cells treated with CRT (0.625 μM) in the presence or absence of BTZ (0.625 nM) was assessed by the CCK-8 assay. Data are presented as mean ± SD. One-way ANOVA with Dunnett's post hoc test or one-way ANOVA with Tukey's post hoc test was performed, n = 3.


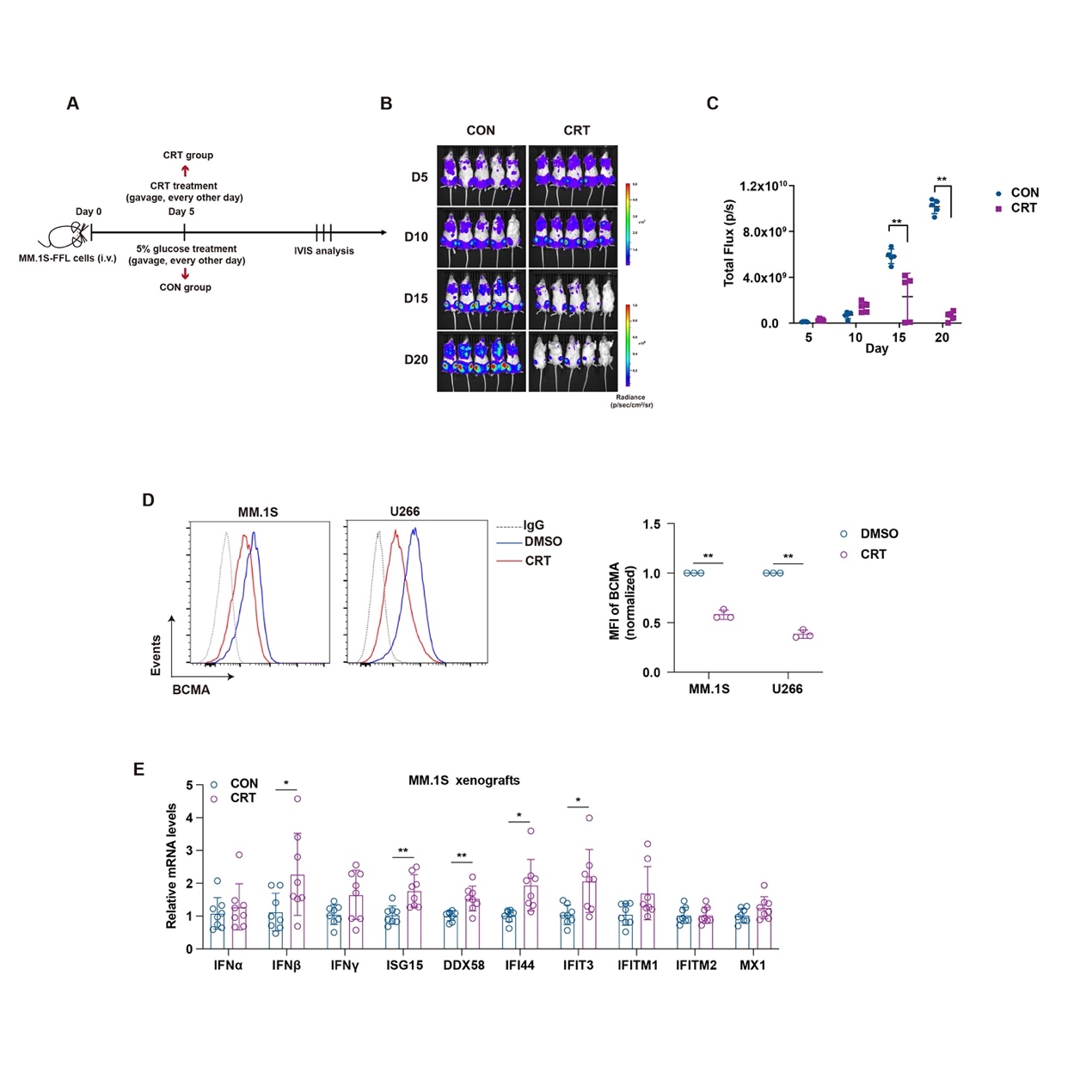


**Figure S25. CRT0066101 (CRT) inhibits tumor growth in vivo, decreases BCMA expression in MM cells and activates the IFN signaling pathway in vivo.**

(A) MM.1S-FFL cells were injected intravenously into NCG mice via the tail vein, starting from 5 days post-inoculation, mice received CRT (80 mg/kg weight) or vehicle (5% glucose in PBS) via gavage every other day. (B) Representative in vivo bioluminescence images of xenograft tumors in NCG mice at different time points. (C) Quantitative analysis of whole-body bioluminescence intensity at different time points. Data are presented as mean ± SD. Unpaired two-tailed *t* test or unpaired two-tailed Welch' *t* test was performed, n = 5. (D) Flow cytometry analysis of BCMA expression in MM.1S or U266 cells following CRT treatment by flow cytometer. Representative results are shown on the left, and summarized results of 3 replications are shown on the right. Data are presented as mean ± SD. Unpaired two-tailed Welch's *t* test was performed, n = 3. (E) RT-qPCR analysis of IFNα, IFNβ, IFNγ, ISG15, DDX58, IFI44, IFIT3, IFITM1, IFITM2 and MX1 in the xenografts from each group. Data are presented as mean ± SD. Unpaired two-tailed t test or unpaired two-tailed Welch' t test was performed, n = 8.


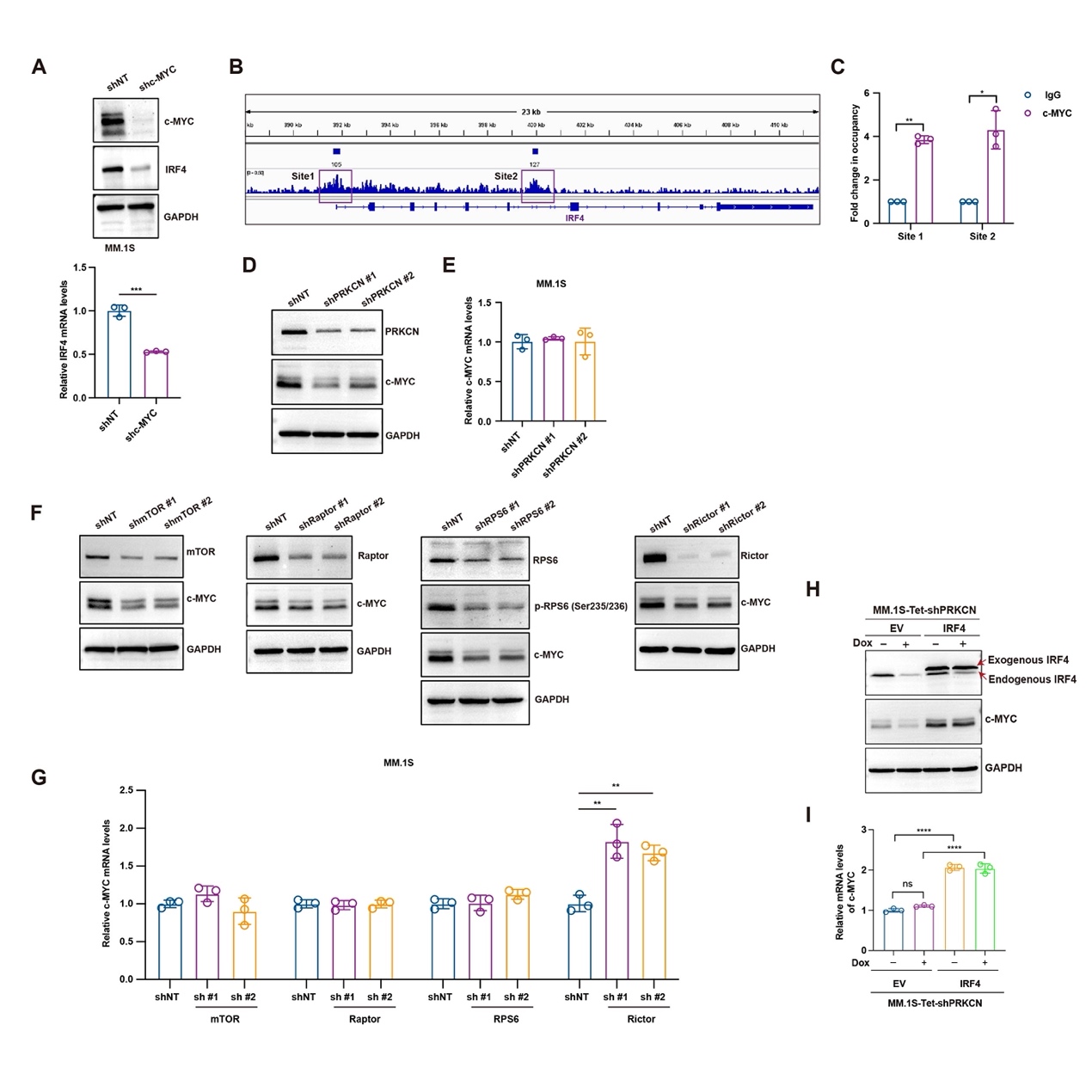


**Figure S26. PRKCN may regulate IRF4 expression by influencing c-MYC expression at post-transcriptional levels.**

(A) Immunoblot analysis of c-MYC and IRF4 expression, and RT-qPCR analysis of IRF4 in MM.1S cells transduced with c-MYC shRNA lentiviruses. (B) The corresponding ChIP-seq data retrieved from SRA databases (SRX129116) were visualized by IGV software to illustrate c-MYC binding to IRF4 promoter in MM.1S cells. (C) ChIP-qPCR analysis showing occupancy of c-MYC at IRF4 proximal promoter in MM.1S cells. Data are presented as mean ± SD. Unpaired two-tailed Welch' *t* test was performed, n = 3. (D-E) Immunoblot analysis of PRKCN and c-MYC, and RT-qPCR analysis of c-MYC in MM.1S transduced with shPRKCN lentiviruses. (F) Immunoblot analysis of c-MYC, mTOR, Raptor, RPS6 or Rictor in MM.1S transduced with shmTOR, shRaptor, shRPS6 or shRictor lentiviruses. (G) RT-qPCR analysis of c-MYC in MM.1S transduced with shmTOR, shRaptor, shRPS6 or shRictor lentiviruses. (H-I) Immunoblot analysis of IRF4 and c-MYC, and RT-qPCR analysis of c-MYC in MM.1S-Tet-shPRKCN cells transduced with Venus or IRF4-overexpressing lentiviruses in the presence or absence of Dox for 3 days. For RT-qPCR, data are presented as mean ± SD. Unpaired two-tailed *t* test or one-way ANOVA with Dunnett's post hoc test was performed, n = 3.


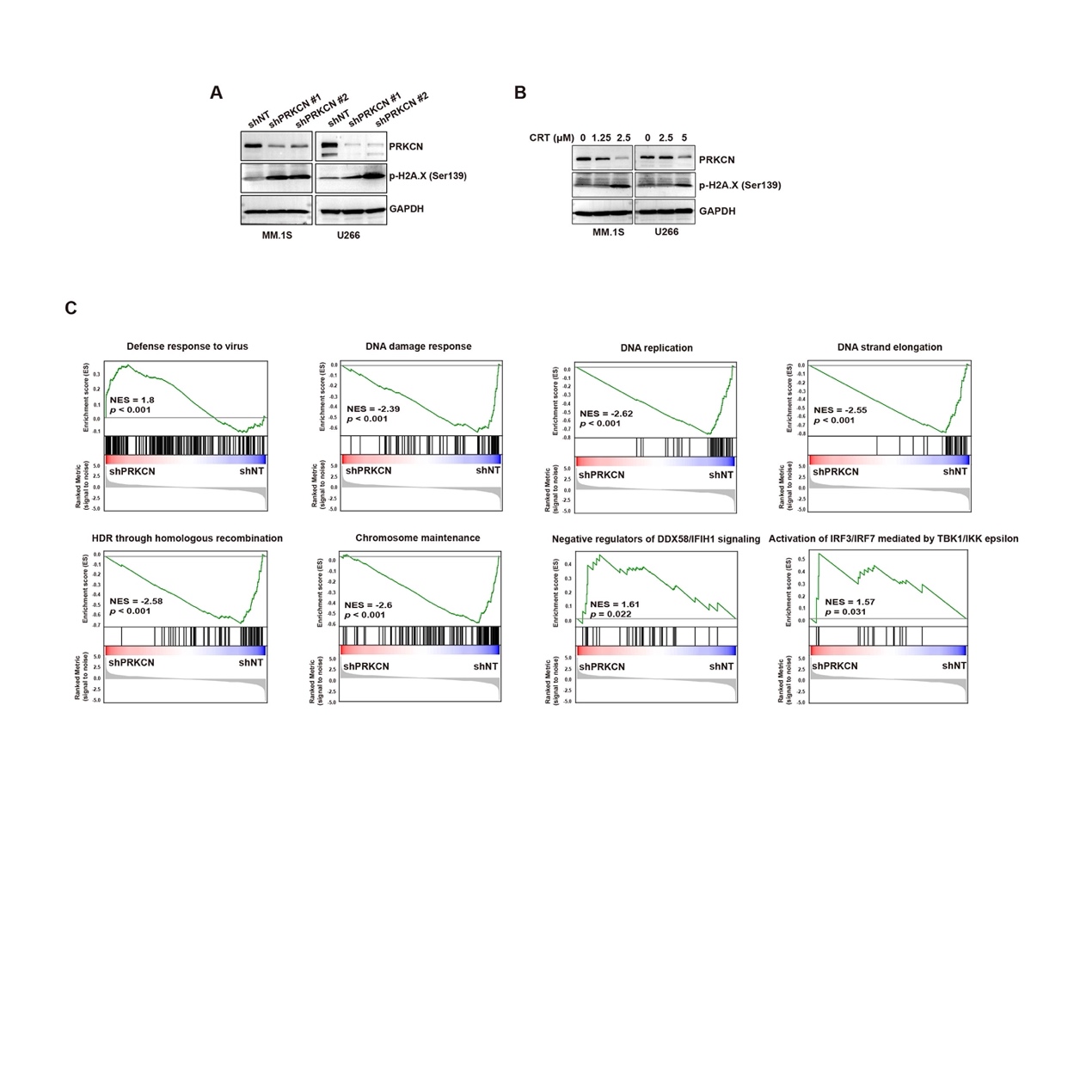


**Figure S27. PRKCN perturbation potentially increases IFNβ expression through evoking DNA sensor pathway and/or dsRNA-sensing pathway in MM cells.**

(A) Immunoblot analysis of PRKCN and p-H2A.X in MM.1S or U266 cells subjected to constitutive PRKCN knockdown. (B) Immunoblot analysis of PRKCN and p-H2A.X in MM.1S or U266 cells subjected to CRT treatment for 24 h. (C) GSEA plots of defense response to virus, DNA damage response, DNA replication, DNA strand elongation, HDR through homologous recombination, chromosome maintenance, negative regulators of DDX58/IFIH1 signaling and activation of IRF3/IRF7 mediated by TBK1/IKK epsilon in shPRKCN versus shNT cells.

**References**

1. Wu L, Xia L, Chen X*, et al.*, “Long non-coding RNA LINC01003 suppresses the development of multiple myeloma by targeting miR-33a-5p/PIM1 axis,” *Leuk Res* (2021): 106565, http://doi.org/10.1016/j.leukres.2021.106565.

2. Nair JR, Caserta J, Belko K*, et al.*, “Novel inhibition of PIM2 kinase has significant anti-tumor efficacy in multiple myeloma,” *Leukemia* (2017): 1715-1726, http://doi.org/10.1038/leu.2016.379.

3. Wang QS, Shi QQ, Meng Y*, et al.*, “Identification of Immune-Related Genes for Risk Stratification in Multiple Myeloma Based on Whole Bone Marrow Gene Expression Profiling,” *Front Genet* (2022): 897886, http://doi.org/10.3389/fgene.2022.897886.

4. Burger KL, Fernandez MR, Meads MB*, et al.*, “CK1δ and CK1ε Signaling Sustains Mitochondrial Metabolism and Cell Survival in Multiple Myeloma,” *Cancer Res* (2023): 3901-3919, http://doi.org/10.1158/0008-5472.Can-22-2350.

5. Tan B, Yang G, Su L*, et al.*, “MiR-125b targeted regulation of MKNK2 inhibits multiple myeloma proliferation and invasion,” *Am J Transl Res* (2024): 3366-3375, http://doi.org/10.62347/qwgs2351.

6. Wan Y, Wang J, Chen M*, et al.*, “Dual roles of IRE1α inhibition in reversing mitochondrial ROS-induced CD8(+) T-cell senescence and exerting direct antitumor effects in multiple myeloma,” *J Immunother Cancer* (2025): http://doi.org/10.1136/jitc-2024-011044.

7. Gardam S, Beyaert R, “The kinase NIK as a therapeutic target in multiple myeloma,” *Expert Opin Ther Targets* (2011): 207-218, http://doi.org/10.1517/14728222.2011.548861.
